# Supplementary material for: Roadmap to the study of gene and protein phylogeny and evolution—A practical guide
Source: PLoS One. 2023 Feb 24;18(2):e0279597. doi: 10.1371/journal.pone.0279597 (PMC9955684; doi:10.1371/journal.pone.0279597)
Supplement: S1 File — These sequences and accession numbers were used for phylogenetic analysis. (PDF) [file pone.0279597.s001.pdf]

SI File 1 (SI F1). Sequences and accession numbers of p53 proteins which were used for phylogenetic analysis.

>XP_001746020.1_OG_p53-like_[Monosiga_brevicollis]
MASQDTLPASQETVPDAETGVFPPMLTRHGSSYKRMSMRDFHEYLSRINKDGDMASGSMVRRDCMMHKDG
WQQVDAEFDVDIDILGGQLPMDTELVDLETPLPGTGLDDTPQASAKPCGALEDSGEPNPAGFRANLADSS
VAAGPGARAIGWTYSPILNTLFTPMDYSCPIRFATNESVPDLSRIVAHLEYTQTNQRNFVVNRCDMHRQG
DSGPFAEHVLRVNNPQANYHQRQERLAVSVPVASTRSGKVEQNELFEWHCLTSCAGGINRRKIRVVFRLI
DPDQNVLGVQHINVRVCACPVRDRRTHEKAHAKREQANDKRNVIASDPTPSKKPKLKDHPISMALRKMPS
VSTLCQIGSGFDHDQDVQYLAICGRANYEVALKVRDQLNQSAGLGRVAASSVHLPRSEVTLIKQRVAEAT
NLDEFLRACNLQSLKRTLENLGYTDLPLIATLDAREIEELTLTKQDQRRLKLALVTLNETDKAQDPYLAR
RRETKLMRVTVRSTPGQAQAGGAGPTSPFAAAS
>XP_002164067.1_OG_p53-like_[Hydra_vulgaris]
MASSSEFETDCSQQTECPLTLDTYNQLKEQFQLDSQELFDLCGSSGEIKTSESFGAVEISESSGAIETSE
SSGEFKPKCLKSVKNKTLSSSSSNTQVNNSYKGRYNFNFNFVVEEEPPTKCCTLTYSKLLNKLFVRTDQF
VILPFSVISLPKKIKLQSFLMYKGKNNIVERCPNHLQKEENLANRHHVLQVNSLTDAVYLKDEMGYKVVS
VPRIYDSTSFSETYKFGCSSQCRWGIDRSPIIIVCQLVFKNSGLYLPVVAVTNGMASSEHIQDMTDVFVQ
FFLSVAKGFLRESFENTVADNVASLKF
>NP_996268.1_p53_[Drosophila_melanogaster]
MYISQPMSWHKESTDSEDDSTEVDIKEDIPKTVEVSGSELTTEPMAFLQGLNSGNLMQFSQQSVLREMML
QDIQIQANTLPKLENHNIGGYCFSMVLDEPPKSLWMYSIPLNKLYIRMNKAFNVDVQFKSKMPIQPLNLR
VFLCFSNDVSAPVVRCQNHLSVEPLTANNAKMRESLLRSENPNSVYCGNAQGKGISERFSVVVPLNMSRS
VTRSGLTRQTLAFKFVCQNSCIGRKETSLVFCLEKACGDIVGQHVIHVKICTCPKRDRIQDERQLNSKKR
KSVPEAAEEDEPSKVRRCIAIKTEDTESNDSRDCDDSAAEWNVSRTPDGDYRLAITCPNKEWLLQSIEGM
IKEAAAEVLRNPNQENLRRHANKLLSLKKRAYELP
>XP_006561045.1_p53_[Apis_mellifera]
MKDEEEEEYFQELDPPIPVREEFPGRHNFQILLVNQDSSKHWVYSQTLKKVFIVMEETLPLRVKWEPPED
GFLLRTAMVFSLDQYASDPVRRCHNHMAPNNPSNRDVDPRVIKHVVRCTHHLTMYEERNEHLSIIIPLNR
PQPGSQYVPVCFKFLCKNSCPSGMNRRPTELIFTLEDRNKRILGRRRLPVRVCSCPKRDKKKEEAEVTDV
QPDIKKRMLCMPVAKKIMPSCDTHVFNVQLSIVGKENYLAVLKYAYDIMAGQAIKTGQLEFFKPYMDDIL
HKTP
>XP_020722577.1_p53_[Bombus_terrestris]
MQSITEEMEEKYDLPQQYLESKKQMKDEEEEQDEEYFQEFDPAIPIKEEFPGRYNFQVSLGNLDSSKNWV
YSQVLKKVFINMEETLPLRFKWEPPEDGLLLRTAMVFSLDQYASDPVRRCHNHMAPNNPSNRDVASRVIK
HVVRCTHHLTMYEERNEHLSIVVPLNRPQPGSHYVPVCFKFLCKNSCPSGMNRRPTELIFTLENCNRRIL
GRQRLHVRVCSCPKRDKKKEEAEVTDAQADIKKRKLCIPVGKKMMPSCDTHVFNVQLNIVGKENYLAVLK
YAYDIMAGQAIKTGQLDFFKPYMDDILRKTP
>XP_021694036.1_p53_[Aedes_aegypti]
MVDSQDTNQTYGQPTQHPIDECDAAQLVQIPTNEFLMDLDGDILDYHEVLKLGSSGSSLMGESGDHYMEQ
KPIVVVDQQTPLMAALLDHMDRTPTLDEIENPVYNFNVDLNGETSGKSSWMFSSRLNKVFVKMGQACTFN
ISYQALTHQELFVRAMMVCSAPEDMHYPVYRCENHRGSDNVNPKLPSEVKAHVMRCFNPSARYVGTENGV
AFKDRLAVIIPLGMTSQEQVALNVSLEFVCQNSCRIINRRTTAIIFTLEDVHGQILGKKSLHLKVCSCPK
RDKMKEEETVAPTKRKSDTQLQAPPGKKIAKVATLSRQIAAQQQQLQQRRLTPSPTSGKLAFIKREISAE
SLNGCNQKARSITPSHSQEMVEANGIPVTVLLPSVEMANKVAEYAFQIVAAELVRSHADQDDSKKLAAYL
TNIRRVQKGLHTGRPGSNTNASTDSD
>NP_001071796.1_p53-like_[Ciona_intestinalis]
MAVADTSELNFPDSQESFSDFWMNTLSENNELPSWQTDLNQEYDQCKETVDVLQLDTTKANDIEFPVSEF
LTSSQASQQSIGDLFAQSLPSTQCSGNSQVTTVSKHEYSDSGYVMTLPNNSVLAELQTSSLQTEVSLLPN
NEYPGIYNFEINFGEKTESAPKSAPFTYSYSLQKLFVKMNENCPIKFRCSPQPPSGCVIRAIPVFEKPNN
VTEIVTRCFNHRNECRTESSDSNTPNSHLIRVESKSNNIQYCLTHEGRECVVVPYEPPHSGSEYMALLYR
FMCLSSCRTETGINRRPLLTIFNLESETGELLGKRVVSTRICACPGRDRTQEEEKKNVTSQNKSRKRLCK
SATNSKSIPVVQKNEENKVDNDDDGDVVYTLNIRGKRKFEKVKEYKEALDLLDYVQPDVKKACCQRNQI
>NP_001258749.1_p53_[Danio rerio]
MAQNDSQEFAELWEKNLISIQPPGGGSCWDIINDEEYLPGSFDPNFFENVLEEQPQPSTLPPTSTVPETS
DYPGDHGFRLRFPQSGTAKSVTCTYSPDLNKLFCQLAKTCPVQMVVDVAPPQGSVVRATAIYKKSEHVAE
VVRRCPHHERTPDGDNLAPAGHLIRVEGNQRANYREDNITLRHSVFVPYEAPQLGAEWTTVLLNYMCNSS

CMGGMNRRPILTIITLETQEGQLLGRRSFEVRVCACPGRDRKTEESNFKKDQETKTMAKTTTGTKRSLVK
ESSSATLRPEGSKKAKGSSSDEEIFTLQVRGRERYEILKKLNDSLELSDVVPASDAEKYRQKFMTKNKKE
NRESSEPKQGKKLMVKDEGRSDSD
RDDWTDFSFDLAPDSRRNKQQRIKEEGE
>XP_005999860.1_p53_[Latimeria_chalumnae]
MLGNSPARVECEPGGSGQGSRGIRVLPEARIGETVSFSCSCGNGVLPEATMTDPMSESGLEPLSQESFAD
LWNLLPTSMSNNVDLLPEETWQAELDDLRLPGEMGAPLQTMTVSSNFGAFLESELEAGLASAAPPPPAPP
APSGESIPVTSSVPSTEDYAGDHNFQLIFQQSSTAKSVTCTYSFTLNKLFCQLAKTCPVQIKVDAAPPPG
ALIRTIAIYKKSEHIAEVVRRCPHHERCADLNDGLAPASHLIRVEGNRLAQYIEDRNTQRQCVIIPYECP
QVGSECSTVLYNFMCNSSCMGGMNRRPILTIITLETKEGRLLGRRCFEVRVCACPGRDRKTEEENSHKQQ
EKANPKTAIKRALREVSCSDSGPYKKRAVNPEEELYSLQVRGRERYEMFKKLNNALELQDSVSAAETEKH
KTKPSKNASRKELEPKKGKKLLVKEEGSGGGGGGGGGGNDSD
>NP_001001903.1_p53_[Xenopus_tropicalis]
MEPSSETGMEPPLSQETFEDLWSLLPDPLQTGTGQMENFAEFSEYPLAPDMTVLQEGLMGNTVPTVTSSA
VPSTEDYAGSYGLKLEFQQNGTAKSVTCTYSTDLNKLFCQLAKTCPLLVRVERPPPLGSILRATAVYKKS
EHVAEVVKRCPHHERSVEPGDDPAPPSHLMRVEGNSKAYYMEDVGTGRHSVCVPYEGPQVGTECTTVLYN
YMCNSSCMGGMNRRPILTIITLESPEGLLLGRRCFEVRVCACPGRDRRTEEDNCTKKRGLKPNGKRELSH
PPSSDPPLPKKRLVEEDDEETFTLLIKGRSRYEMIKKLNDALELQESLDQQKLSIKCRKCRDEIKPKKGK
KLLVKDELQDSE
>XP_008123458.1_p53_[Anolis_carolinensis]
MASAASSRGNGRDLEPGDSPAHGCCAVPCLAPGDFHLEAMQRRGGMEDPSLLDSDFGMMEMDPNEVLDLV
PMPSSPLSQDTFQTLWSSLLDGKEDQSLLNPDLPYPMSYEEGTSPKAAEGDYSTLEPVSSVFVSTPIIPS
TEDYVGDHGFELAFEQSGTAKSVTCTYSPKLNKLFCQLAKTCPVHIKVANMPPLGAVIRTMAVYKKSEHV
AEVVKRCPHHERSQEFSDSSAPAEHLIRVEANQQARYIADPNRRHSVIVPYEPPQVGTDNTTLLLNFMCN
SSCMGGMNRRAILAIITLETLQGDLLGRRCFEVRVCACPGRDRKSEEENALKAAVPKGKSKKVVLPAAPR
SNSSENAKRSAAGSSNHETESGPYILQVRNRKHYRMLKMILEGLELREKQQGEEEEEAEPETRCLPKAPK
RKRLRVKDENPDSD
>NP_990595.1_p53_[Gallus_gallus]
MAEEMEPLLEPTEVFMDLWSMLPYSMQQLPLPEDHSNWQELSPLEPSDPPPPPPPPPLPLAAAAPPPLNP
PTPPRAAPSPVVPSTEDYGGDFDFRVGFVEAGTAKSVTCTYSPVLNKVYCRLAKPCPVQVRVGVAPPPGS
SLRAVAVYKKSEHVAEVVRRCPHHERCGGGTDGLAPAQHLIRVEGNPQARYHDDETTKRHSVVVPYEPPE
VGSDCTTVLYNFMCNSSCMGGMNRRPILTILTLEGPGGQLLGRRCFEVRVCACPGRDRKIEEENFRKRGG
AGGVAKRAMSPPTEAPEPPKKRVLNPDNEIFYLQVRGRRRYEMLKEINEALQLAEGGSAPRPSKGRRVKV
EGPQPSCGKKLLQKGSD
>NP_776626.1_p53_[Bos_taurus]
MEESQAELNVEPPLSQETFSDLWNLLPENNLLSSELSAPVDDLLPYTDVATWLDECPNEAPQMPEPSAPA
APPPATPAPATSWPLSSFVPSQKTYPGNYGFRLGFLQSGTAKSVTCTYSPSLNKLFCQLAKTCPVQLWVD
SPPPPGTRVRAMAIYKKLEHMTEVVRRCPHHERSSDYSDGLAPPQHLIRVEGNLRAEYLDDRNTFRHSVV
VPYESPEIDSECTTIHYNFMCNSSCMGGMNRRPILTIITLEDSCGNLLGRNSFEVRVCACPGRDRRTEEE
NLRKKGQSCPEPPPRSTKRALPTNTSSSPQPKKKPLDGEYFTLQIRGFKRYEMFRELNDALELKDALDGR
EPGESRAHSSHLKSKKRPSPSCHKKPMLKREGPDSD
>NP_000537.3_p53_isoform_a_[Homo_sapiens]
MEEPQSDPSVEPPLSQETFSDLWKLLPENNVLSPLPSQAMDDLMLSPDDIEQWFTEDPGPDEAPRMPEAA
PPVAPAPAAPTPAAPAPAPSWPLSSSVPSQKTYQGSYGFRLGFLHSGTAKSVTCTYSPALNKMFCQLAKT
CPVQLWVDSTPPPGTRVRAMAIYKQSQHMTEVVRRCPHHERCSDSDGLAPPQHLIRVEGNLRVEYLDDRN
TFRHSVVVPYEPPEVGSDCTTIHYNYMCNSSCMGGMNRRPILTIITLEDSSGNLLGRNSFEVRVCACPGR
DRRTEEENLRKKGEPHHELPPGSTKRALPNNTSSSPQPKKKPLDGEYFTLQIRGRERFEMFRELNEALEL
KDAQAGKEPGGSRAHSSHLKSKKGQSTSRHKKLMFKTEGPDSD
>NP_694518_p63_[Danio_rerio]
MLYLETNAPSSYSEPQYTSLGLLNSMDQNGGSTSTSPYNNDHAQNNVTAPSPYAQPSSTF
EALSPSPAIPSNTDYAGPHTFDVSFQQSSTAKSATWTYSTELKKLYCQIAKTCPIQIKVL
TNPPQGAVIRAMPVYKKAEHVTEVVKRCPNHELSREFNDGQIAPPSHLIRVEGNSHAQYV
EDSITGRQSVLVPYEPPQVGTEFTTILYNFMCNSSCVGGMNRRPILIIVTLETRDGQVLG
RRCFEARICACPGRDRKADEDSIRKQHVTDGTKSSEAFRQASSHLSQLNSIKKRRSTDEE
VFCLPIKGREIYEILVKIKESLELMQFLPQQTIESYRQQQQNLLQKQSSLPPQPAFGSSS
PTLGKNKLPSVSQLINPQQRNALTPSGMPGGLTDSLLQSQPFPLPSVTPPMMGGPVPMNT
DLSSLSPNNPLQSQLQMVPSSHCTPPPPYPMDNSISSFLLRLGCSACLDYFTAQGLTNIY

QIENYNLEDLSRLKIPTEFQHIIWKGIMEYRQTMEFSPPPHILRTSSGTSLVSVGSTEAR
SERVIDAVRFTLRQTISFPPRDDWTDFSFDLAPDSRRNKQQRIKEEGE
>XP_002934096.2_p63_[Xenopus_tropicalis]
MSQSSQDCERSSDVFDRLWEFLGQPMHSVQPIDLNFTSDAMEKGPRNKIEISMDCIRMQDCDVSDSMWPQ
YTNLGLLNSMEQQIQNGSSSTSPYANDHAQNSVTAPSPYAQPSSTFDALSPSPAIPSNTDYPGPHSFDVS
FQQSSTAKSATWTYSTDLKKLYCQIAKTCPIQVKVMTPPPQGAVVRAMPVYKKAEHVTEVVKRCPNHELS
REFNEGQIAPPSHLIRVEGNSHAQYVEDPITGRQSVLVPYEPPQVGTEFTTILYNFMCNSSCVGGMNRRP
ILIIVTLETRDGQVLGRRCFEARICACPGRDRKADEDSIRKQQVSDSTKNGEGMLINKELTKMTFRQSTH
GIQMTSIKKRRSPDDEVLYLPVKGREIYEMLLKIKESLELMQFLPQHTIESYRQQQQHLLQKQSSLPGQS
SFGSTSPPLGKMNNMNKLPSVSQLMNPQQRNSLTPNAMSDGMGANIPMMSTHMPMTSDLNGLSPSQTLPP
SLSLPSTSHCTPPPPYPSDCSIASFLARLGCSSCLDYFTTQGLNTIYQIENYSIEDLASLKIPDQFRHAI
WKGLMEHRQMHDFTSPPHLLRTTSSASTVSVGSNEPRGERVIDAVRFTLRQTISFPPRDDWNDFNFDLDT
RRNKQQRIKEEGE
>XP_008117588.1_p63_[Anolis_carolinensis]
RNKAQPGLTFGGLTWGHAEGDGGTPLPTLYSTELKKLYCQIAKTCPIQIKVMTPPPQGAVIRAMPVYKKA
EHVTEVVKRCPNHERSREFNEGQIAPPSHLIRVEGNSHAQYVEDPITGRQSVLVPYEPPQVGTEFTTVLY
NFMCNSSCVGGMNRRPILIIVTLETRDGQVLGRRCFEARICACPGRDRKADEDSIRKQQVSDGTKNGDAF
RQSTHGIQMTSVKKRRTPDDELLYLPVRGRETYEMLLKIKESLELMQYLPQHTIETYRQQQQQQQHQHL
LQKQASLQSPSSYGSNSPPLGKMNSVNKLPSVSQLMNPQQRNALTPTSLPDSMGANIPMMGHMAMAGDMN
GLSPTQALPPPLSMPSTSHCTPPPPYPTDCSIVSFLARLGCSSCVDYFTAQGLTTIYQIEHYSMDDLVSL
KIPEQFRHAIWKGLMDHRQLHDFSSSTPHLLRTPSGASTVSVGSSETRGERVIDAVRFTLRQTISFPPRD
EWNDFNFDLDARRNKQQQRIKEEGE
>NP_001178266.1_p63_[Bos_taurus]
MNFETSRCATLQYCPDPYIQRFVETPAHFSWKESYYRSTMSQSTQTSEFLSPEVFQHIWDFLEQPICSVQ
PIDLNFVDEPSENGATNKIEISMDCIRMQDSDLGDPMWPQYTNLGLLNSMDQQIQNGSSSTSPYNTDHAQ
NSVTAPSPYAQPSSTFDALSPSPAIPSNTDYPGPHSFDVSFQQSSTAKSATWTYSTELKKLYCQIAKTCP
IQIKVMTPPPQGAVIRAMPVYKKAEHVTEVVKRCPNHELSREFNEGQIAPPSHLIRVEGNSHAQYVEDPI
TGRQSVLVPYEPPQVGTEFTTVLYNFMCNSSCVGGMNRRPILIIVTLETRDGQVLGRRCFEARICACPGR
DRKADEDSIRKQQVSDSTKNGDGTKRPFRQNTHGIQMTSIKKRRSPDDELLYLPVRGRETYEMLLKIKES
LELMQYLPQHTIETYRQQQQQQHQHLLQKQTSMQSQSSYGNSSPPLNKMNSMNKLPSVSQLINPQQRNAL
TPTTIPDGMGANIPMMGTHMPMAGDMNGLSPTQALPPPLSMPSTSHCTPPPPYPTDCSLVSFLARLGCSS
CLDYFTTQGLTTIYQIEHYSMDDLASLKIPEQFRHAIWKGILDHRQLHDFSSPPHLLRTPSGASTVSVGS
SETRGERVIDAVRFTLRQTISFPPRDEWNDFNFDMDARRNKQQRIKEEGE
>NP_989682.1_p63_[Gallus_gallus]
MLYLENNAQSQYSEPQYTNLGLLNSMDQQVQNGSSSTSPYNTEHAQNSVTAPSPYAQPSSTFDALSPSPA
IPSNTDYPGPHSFDVSFQQSSTAKSATWTYSTELKKLYCQIAKTCPIQIKVMTPPPQGAVIRAMPVYKKA
GHVTEVVKRCPNHELSREFNEGQIAPPSHLIRVEGNSHAQYVEDPITGRQSVLVPYEPPQVGTEFTTVLY
NFMCNSSCVGGMNRRPILIIVTLETRDGQVLGRRCFEARICACPGRDRKADEDSIRKQQVSDSTKNGDAF
RQGTHGIQMTSIKKRRSPDDELLYLPVRGRETYEMLLKIKESLEPMQYLPQHTIETYRQQQQQHQHLLQ
KQTSIQSQSSYGSNSPPLSKMNSMNKLPSVSQLINPQQRNALTPTTIPDGMGTNIPMMGTHMAMTGDMNV
LSPTQALPPPLSMPSTSHCTPPPPYPTDCSIVSFLARLGCSSCVDYFTTQGLTTIYHIEHYSMDDLVSLK
IPEQFRHAIWKGILDHRQLHDFSSPPHLLRTPSGASTVSVGSSETRGERVIDAVRFTLRQTISFPPRDEW
NDFNFDMDARRNKQQRIKEEGE
>NP_003713.3_p63_[Homo_sapiens]
MNFETSRCATLQYCPDPYIQRFVETPAHFSWKESYYRSTMSQSTQTNEFLSPEVFQHIWDFLEQPICSVQ
PIDLNFVDEPSEDGATNKIEISMDCIRMQDSDLSDPMWPQYTNLGLLNSMDQQIQNGSSSTSPYNTDHAQ
NSVTAPSPYAQPSSTFDALSPSPAIPSNTDYPGPHSFDVSFQQSSTAKSATWTYSTELKKLYCQIAKTCP
IQIKVMTPPPQGAVIRAMPVYKKAEHVTEVVKRCPNHELSREFNEGQIAPPSHLIRVEGNSHAQYVEDPI
TGRQSVLVPYEPPQVGTEFTTVLYNFMCNSSCVGGMNRRPILIIVTLETRDGQVLGRRCFEARICACPGR
DRKADEDSIRKQQVSDSTKNGDGTKRPFRQNTHGIQMTSIKKRRSPDDELLYLPVRGRETYEMLLKIKES
LELMQYLPQHTIETYRQQQQQQHQHLLQKQTSIQSPSSYGNSSPPLNKMNSMNKLPSVSQLINPQQRNAL
TPTTIPDGMGANIPMMGTHMPMAGDMNGLSPTQALPPPLSMPSTSHCTPPPPYPTDCSIVSFLARLGCSS
CLDYFTTQGLTTIYQIEHYSMDDLASLKIPEQFRHAIWKGILDHRQLHEFSSPSHLLRTPSSASTVSVGS
SETRGERVIDAVRFTLRQTISFPPRDEWNDFNFDMDARRNKQQRIKEEGE
>XP_005992519.1_p63_[Latimeria chalumnae]
MNLETSPYSTLQYCHDPRFQRFIETPAHFSWTENYFHSTMSQNSETSNILTPDVSSDVLQHLLDKFQPMC
SELPIELRFTEGPCGSAPTNSIEISMDYFRMHDSDITDPMWPQYTNLGILNSMDQQLPNGSSSTSPYNTE

HAQNTVTAPSPYAQPSSTFDALSPSPAIPSNTDYPGPHSFDVSFQQSSTAKSATWTYSTELKKLYCQIAK
TCPIQIKVMTPPPQGAVIRAMPVYKKAEHVTEVVKRCPNHELSREFNEGQVAPPSHLIRVEGNSHSQYVE
DHITGRQSVLVPYEPPQVGTEFTTILYNFMCNSSCVGGMNRRPILIIVTLESRDGQVLGRRCFEARICAC
PGRDRKADEDSIRKQQVSEDAKNGDGTKRPFRQTPQGIQIKKRRSTDDEVLYIAVRGRENYEVLQKVRES
LELAQFLPQHVIEHYRQQQQHLVQKQPSMQSQPSYGSASPPLNKMANMNKLPSVSQLINPQHRNALTPNT
IQEGMSANIPQLVSTHMPMPSDLNGLTPTQALPPPLTMQSATHCTPPPPYPSDCSISSFLARVGCSSCLE
YFTTQGLTTIYQIEHFSMDDLVGLKIPEQFRNAIWKGLLDHRQVPDYCSPPHLLRTNSGASTVSVGSNET
REERVIDAVRFTLRHTISFPPRDEWNDFNFDIDARRNKQRIKEEGE
>XP_005986096.1_p73_[Latimeria_chalumnae]
MKDWRWIPLQKVISPSLDYLTTKMAQSSPDDEGTTFEHLWSTLEPDSTYFDLPQSNHSGGSEGVTSLSSN
QAEVSMDIFQMRGMNESVMSQFNMLNNTMDQSIGSRAASTSPYSSEHPLNVPTHSPYSQPNSTFDAMSPA
PAIPSNTDYPGPHNFEVTFQQSSTAKSATWTYSPLLKKLYCQIAKTCPIQIKVSTPPPPASVIRAMPVYK
KAEHVTEVVKRCPNHELGRDFNDSQSAPASHLIRVEGNNLAQYVDDPVSGRQSVFVPYEPPQVGTEFTTI
LYNFMCNSSCVGGMNRRPIVIIITLETRDGQVLGRRSFEGRICACPGRDRKADEDHFREQQAMNESAAKN
GNGNKRTFKQNPQGIPGLGVSIKKRKHGEEEIYYVPVRGRENFEILMKIKESLELVELVPQQLVESYRQQ
QQQLLQRQNHWQPPTSYGPVLSPMNKVHGGMNKLPSVNQLVGQPSQHNNSVSNLGPMGPGMLNSHHMQPN
GDINGGHSSQSMVSTSHCTPPPPYNPDPSFVSFLTSLGCPNCIEYFTSQGLQNMYQLQNLSMEDLGALKI
PDQYRMIIWRGLQELKQSQEYTPQQLIRSSSNTSTIAIGASGELQRQRVMEAVHFRVRHTITIPNRTDDW
ADFGFDVPDCKARKQSIKEEFTDSELN
>XP_002933901.3_p73_[Xenopus_tropicalis]
MQATLIKQRAVQGAISTDWHFRVIYPLPESPILKMSQSSGADEGTTFEHLWSTLEPDSTYFELPQSSHSN
NTEASNRTDVNMDVYQMRTMNESIMSQFNLLNNTMDQSIGSRAASTSPYNPDHTSNVPTHSPYSQPSSTF
DAMSPAPVIPSNTDYPGTHNFEVTFQQSSTAKSATWTYSPLLKKLYCQIAKTCPIQIKLSNPPPPGSVIR
AMPVFKKAEHVTEVVKRCPNHELGRDFNDGQAAPASHLIRVEGNNLSQYVDDPVTGRQSVMVPYEPPQVG
TEFTTILYNFMCNSSCVGGMNRRPILIIITLETRDGQVLGRRSFEGRICACPGRDRKADEDHFREQAALN
ETAAKNGNANKRTFKQSPPSVPSMGSNIKKRRHGEDEIFYIPVRGRENFEILMKIKESLELVELVPQQLV
DSYRQQQQQLLQRQTHLQSTSSYGPVLSPMNKLHGGINKLPSVNQLVGQPNQHNSNAGPNMGPMGPSMLN
SHPLQTNGEMNGAHSSQSMVSGSHCTPPPPYNADPSLVSFLTGLGCPNCIEYFTSQGLQNIYHLQNLTME
DLGALKIPEHYKSMIWRGIQELNKSHEYGAQQLVRSSSNASTISIGSSGELQRQRVMEAVHFRVRHTITI
PNRGGADEWADFGFDLPDCKSRKQSIKEEYENSDIN
>NP_899183.1_p73_[Danio_rerio]
MSQSSTADEGPTFEHLWSTLEPDSTYFELPQAGHSGDRASSSLPGNRAEVCMDVYHMRDMRDMNDNVMSQ
YSLLSSSMDQGLGNRAASTSPYSSETTSNVPTPSPYSQPNSTFEAMSPAPAIPSNTDYPGPHNFEVTFQQ
SSTAKSATWTYSPLLKKLYCQIAKTCPIQIKLASSPPNGSVIRAMPIYKKAEHVTEVVKRCPNHKLGRDF
NESQTAPASHLIRVEGNNLCQYVDDPVTGRQSVLVPYESPQVGTEFTTILYNFMCNSSCVGGMNRRPILI
IITLETRDGQVLGRRSFEGRICACPGRDRKADEDHFREQQALNESVAKNGNANKRNFKQTPTNITGPSIN
IKKRRHGEEEMYYIPVRGRENFDILMKIKDSLELVEFVPQQLVDSYRQQQQQLLQRQNHVASPSSYGTLN
NMNKIHGPISKLPSVNQLVTQQTQQSAGPSASLSHMGANMLGGHHMQSNGDVNGAHQSQSIVSTSHCTPP
PPYNPDPSLVSFLTSLGCQNCIDYFTSQGLQSVYHLQTLTMEDLGALKIPEQFRLAIWRGLQEMKQGHDY
GQQLIRSSSNMATMAIGPSGELQRQRVMEAVHFRVRHTITIPNRGPANGPEEWPDFGFDMPDCRLHKHSI
KEEFAEGDVH
>XP_008122218.1_p73_[Anolis_carolinensis]
MSRSSPADEGTTFEHLWSTLEPDSTYFDLPQSGPRTGSETPNRTEVTMDVFQMRSMNTSVMSQFNLLNGS
MDQSTDSRAASSSPYNSEHASNVPTLSPYSQPSSTFDTMSPAPVIPSNTDYPGPHHFEVTFQQSSTAKSA
TWTYSPLLKKLYCQIAKTCPIQIKVSGPPPPGTIIRAMPVYKKAEHVTEVVKRCPNHELGRDFNDGLSAP
ASHLIRVEGNNLSQYVDDPVTGRQSVMVPYEPPQVGTEFTTILYNFMCNSSCVGGMNRRPILIIITLETR
DGQVLGRRSFEGRICACPGRDRKADEDHYREQQALNESAAKNGNPNKRTFKQSPQGMPALGAGMKKRRHG
EEETYYVPVRGRENFEILMKIKESMELVELVPQQLVDSYRQQQQQLLQRQTHLQPPSSYGPVLSPMNKLH
AGGVNKLPSVNQLVGQPQQHGPNTGPNLGPMGPGILNSHPMQSNGDMNGGHSSQSMVSGAHCSPPPPYNP
DPSLVSFLTGLGCSNCIDYFTSQGLQTLYQLQNLTLEDLVLLKIPEQYRMIIWRGLQDLKQGHDYGAQQL
IRSSGNASTISIGASGELQRQRVMEAVHFRVRHTITIPNRGGADEWADFGFDLPDCKSRKHSIKEEFTEG
EIN
>XP_417545.3_p73_[Gallus_gallus]
MSQSSPADESTTFEHLWSTLAPDSTYFDLSPSSHTGSNEVSNRTEVTMDVFQMRGMNDSVMSQFNLLNNS
MDQSIGSRAASTSPYSSEHTSNVPTHSPYSQPSSTFDAMSPAPVIPSNTDYPGPHHFEVTFQQSSTAKSA
TWTYSPLLKKLYCQIAKTCPIQIKVSSPPPPGTIIRAMPVYKKAEHVTEVVKRCPNHELGRDFNDGQSAP
ASHLIRVEGNNLSQYVDDPVTGRQSVMVPYEPPQVGTEFTTILYNFMCNSSCVGGMNRRPILIIITLETR

DGQVLGRRSFEGRICACPGRDRKADEDHYREQQALNENAAKNGNANKRTFKQSPQAIPALGPGVKKRRHG
EEEMYYVPVRGRENFEILMKIKESLELVELVPQQLVDSYRQQQQQLLQRQNQLQTPSSYGPVLSPMNKAH
GGGINKLPSVNQLVGQPAQHSSGSAPSLGPMGPGMLNSHPMQPNGEMNGGHSSQSMVSGSHCTPPPPYNP
DPSLVSFLTGLGCPNCIDYFTSQGLQNIYHLQNLSIEDLGALKIPEQYRMIIWRGLQELKQSHDYGAQQL
IRSSSNASTISIGSSGELQRQRVMEAVHFRVRHTITIPNRGAADEWADFGFDLPDCKSRKQSIKEEFTEG
EIN
>XP_002694165.1_p73_[Bos_taurus]
MSQSTQPAAADEGATFQHLWSSLEPDSTYFDLPQPGQGNEEVAGGAEAGMDVFHLPGMTTSVMSQFNLLS
STMDQMSSRAASASPYTPEHAASVPTHSPYAQPSSTFDTMSPAPVIPSNTDYPGPHHFEVTFQQSSTAKS
ATWTYSPLLKKLYCQIAKTCPIQIKVSAPPPPGTAIRAMPVYKKAEHVTEVVKRCPNHELGRDFNEGQSA
PASHLIRVEGNNLSQYVDDPVTGRQSVMVPYEPPQVGTEFTTILYNFMCNSSCVGGMNRRPILIIITLET
RDGQVLGRRSFEGRICACPGRDRKADEDHYREQQALNESAAKSGAASKRAFKQSPPTAPALGTNVKKRRH
GDDDVYYIHVRGRENFEILMKVKESLELMELVPQQLVDSYRQQQQQLLQRPSHLQPPSYGPVLSPMSKAH
GAVNKLPSVNQLVGQPPPHGSAAGPNLGPMGPGILNNHGHTLPANGEMNGGPSSQSMVSGSHCTPPPPYH
ADSSLVSFLTGLGCPNCIEYFTSQGLQNIYHLQNLTIEDLGALKIPDQYRMTIWRGLQDLKQSHDYSAQQ
LIRSSSNAATIAIGGSGELQRQRVMEAVHFRVRHTITIPNRGGPAGGAGPDEWADFGFDLPDCKSRKQSI
KEEFTESEAN
>NP_005418.1_p73_[Homo_sapiens]
MAQSTATSPDGGTTFEHLWSSLEPDSTYFDLPQSSRGNNEVVGGTDSSMDVFHLEGMTTSVMAQFNLLSS
TMDQMSSRAASASPYTPEHAASVPTHSPYAQPSSTFDTMSPAPVIPSNTDYPGPHHFEVTFQQSSTAKSA
TWTYSPLLKKLYCQIAKTCPIQIKVSTPPPPGTAIRAMPVYKKAEHVTDVVKRCPNHELGRDFNEGQSAP
ASHLIRVEGNNLSQYVDDPVTGRQSVVVPYEPPQVGTEFTTILYNFMCNSSCVGGMNRRPILIIITLEMR
DGQVLGRRSFEGRICACPGRDRKADEDHYREQQALNESSAKNGAASKRAFKQSPPAVPALGAGVKKRRHG
DEDTYYLQVRGRENFEILMKLKESLELMELVPQPLVDSYRQQQQLLQRPSHLQPPSYGPVLSPMNKVHGG
MNKLPSVNQLVGQPPPHSSAATPNLGPVGPGMLNNHGHAVPANGEMSSSHSAQSMVSGSHCTPPPPYHAD
PSLVSFLTGLGCPNCIEYFTSQGLQSIYHLQNLTIEDLGALKIPEQYRMTIWRGLQDLKQGHDYSTAQQL
LRSSNAATISIGGSGELQRQRVMEAVHFRVRHTITIPNRGGPGGGPDEWADFGFDLPDCKARKQPIKEEF
TEAEIH
