# Supplementary material for: Roadmap to the study of gene and protein phylogeny and evolution—A practical guide
Source: PLoS One. 2023 Feb 24;18(2):e0279597. doi: 10.1371/journal.pone.0279597 (PMC9955684; doi:10.1371/journal.pone.0279597)
Supplement: S2 File — These sequences and accession numbers were used for phylogenetic analysis. (PDF) [file pone.0279597.s002.pdf]

SI File 4 (SI F4). Nexus coding to reconstruct the cophylogeny of human CDKs and Cyclins.

```
#NEXUS

BEGIN HOST;

	tree host =
((((((HsaCyclinB2,HsaCyclinB1),HsaCyclinB3),(HsaCyclinA1,HsaCyclinA2)),(HsaCyclinE1,HsaCyclinE2)),(HsaCyclinD1,(HsaCyclinD3,HsaCyclinD2))),(HsaCyclinC,(HsaCyclinH,((HsaCyclinT2,HsaCyclinT1),(HsaCyclinL2,HsaCyclinL1))))),HsaCyclinY);

	ENDBLOCK;

BEGIN PARASITE;

	tree parasite =
((((((HsaCDK3,HsaCDK2),HsaCDK1),(HsaCDK5,(HsaCDK16,HsaCDK14))),(HsaCDK6,HsaCDK4)),(HsaCDK11,(HsaCDK9,(HsaCDK13,HsaCDK12)))),HsaCDK7,HsaCDK8);

	ENDBLOCK;

BEGIN DISTRIBUTION;

	RANGE

		HsaCDK1: HsaCyclinA1,

		HsaCDK1: HsaCyclinA2,

		HsaCDK1: HsaCyclinB1,

		HsaCDK1: HsaCyclinB2,

		HsaCDK1: HsaCyclinE1,

		HsaCDK1: HsaCyclinE2,

		HsaCDK2: HsaCyclinA1,

		HsaCDK2: HsaCyclinA2,

		HsaCDK2: HsaCyclinE1,

		HsaCDK2: HsaCyclinE2,

		HsaCDK2: HsaCyclinD1,

		HsaCDK2: HsaCyclinD2,

		HsaCDK2: HsaCyclinD3,

		HsaCDK2: HsaCyclinB3,

		HsaCDK3: HsaCyclinC,
```

HsaCDK3: HsaCyclinE1,

HsaCDK3: HsaCyclinE2,

HsaCDK3: HsaCyclinA1,

HsaCDK3: HsaCyclinA2,

HsaCDK4: HsaCyclinD1,

HsaCDK4: HsaCyclinD2,

HsaCDK4: HsaCyclinD3,

HsaCDK5: HsaCyclinE1,

HsaCDK5: HsaCyclinE2,

HsaCDK5: HsaCyclinD1,

HsaCDK5: HsaCyclinD2,

HsaCDK5: HsaCyclinD3,

HsaCDK6: HsaCyclinD1,

HsaCDK6: HsaCyclinD2,

HsaCDK6: HsaCyclinD3,

HsaCDK7: HsaCyclinH,

HsaCDK8: HsaCyclinC,

HsaCDK9: HsaCyclinT1,

HsaCDK9: HsaCyclinT2,

HsaCDK11: HsaCyclinL1,

HsaCDK11: HsaCyclinL2,

HsaCDK12: HsaCyclinL1,

HsaCDK12: HsaCyclinL2,

HsaCDK13: HsaCyclinL1,

HsaCDK13: HsaCyclinL2,

HsaCDK14: HsaCyclinY,

HsaCDK16: HsaCyclinY,

;

ENDBLOCK;
