# Supplementary material for: Roadmap to the study of gene and protein phylogeny and evolution—A practical guide
Source: PLoS One. 2023 Feb 24;18(2):e0279597. doi: 10.1371/journal.pone.0279597 (PMC9955684; doi:10.1371/journal.pone.0279597)
Supplement: S3 File — These sequences and accession numbers were used for phylogenetic analysis. (PDF) [file pone.0279597.s003.pdf]

SI File 2 (SI F2). Sequences of human CDKs which were used for phylogenetic analysis.

>Hsa-CDK7
MALDVKSRAKRYEKLDFLGEGQFATVYKARDKNTNQIVAIKKIKLGHRSEAKDGINRTALREIKLLQELSHPNIIGLLDA
FGHKSNISLVFDFMETDLEVIIKDNSLVLTPSHIKAYMLMTLQGLEYLHQHWILHRDLKPNNLLLDENGVLKLADFGLAK
SFGSPNRAYTHQVVTRWYRAPELLFGARMYGVGVDMWAVGCILAELLLRVPFLPGDSDLDQLTRIFETLGTPTEEQWPDM
CSLPDYVTFKSFPGIPLHHIFSAAGDDLLDLIQGLFLFNPCARITATQALKMKYFSNRPGPTPGCQLPRPNCPVETLKEQ
SNPALAIKRKRTEALEQGGLPKKLIF
>Hsa-CDK3
MDMFQKVEKIGEGTYGVVYKAKNRETGQLVALKKIRLDLEMEGVPSTAIREISLLKELKHPNIVRLLDVVHNERKLYLVF
EFLSQDLKKYMDSTPGSELPLHLIKSYLFQLLQGVSFCHSHRVIHRDLKPQNLLINELGAIKLADFGLARAFGVPLRTYT
HEVVTLWYRAPEILLGSKFYTTAVDIWSIGCIFAEMVTRKALFPGDSEIDQLFRIFRMLGTPSEDTWPGVTQLPDYKGSF
PKWTRKGLEEIVPNLEPEGRDLLMQLLQYDPSQRITAKTALAHPYFSSPEPSPAARQYVLQRFRH
>Hsa-CDK2
MENFQKVEKIGEGTYGVVYKARNKLTGEVVALKKIRLDTETEGVPSTAIREISLLKELNHPNIVKLLDVIHTENKLYLVF
EFLHQDLKKFMDASALTGIPLPLIKSYLFQLLQGLAFCHSHRVLHRDLKPQNLLINTEGAIKLADFGLARAFGVPVRTYT
HEVVTLWYRAPEILLGCKYYSTAVDIWSLGCIFAEMVTRRALFPGDSEIDQLFRIFRTLGTPDEVVWPGVTSMPDYKPSF
PKWARQDFSKVVPPLDEDGRSLLSQMLHYDPNKRISAKAALAHPFFQDVTKPVPHLRL
>Hsa-CDK5
MQKYEKLEKIGEGTYGTVFKAKNRETHEIVALKRVRLDDDDEGVPSSALREICLLKELKHKNIVRLHDVLHSDKKLTLVF
EFCDQDLKKYFDSCNGDLDPEIVKSFLFQLLKGLGFCHSRNVLHRDLKPQNLLINRNGELKLADFGLARAFGIPVRCYSA
EVVTLWYRPPDVLFGAKLYSTSIDMWSAGCIFAELANAGRPLFPGNDVDDQLKRIFRLLGTPTEEQWPSMTKLPDYKPYP
MYPATTSLVNVVPKLNATGRDLLQNLLKCNPVQRISAEEALQHPYFSDFCPP
>Hsa-CDK1
MEDYTKIEKIGEGTYGVVYKGRHKTTGQVVAMKKIRLESEEEGVPSTAIREISLLKELRHPNIVSLQDVLMQDSRLYLIF
EFLSMDLKKYLDSIPPGQYMDSSLVKSYLYQILQGIVFCHSRRVLHRDLKPQNLLIDDKGTIKLADFGLARAFGIPIRVY
THEVVTLWYRSPEVLLGSARYSTPVDIWSIGTIFAELATKKPLFHGDSEIDQLFRIFRALGTPNNEVWPEVESLQDYKNT
FPKWKPGSLASHVKNLDENGLDLLSKMLIYDPAKRISGKMALNHPYFNDLDNQIKKM
>Hsa-CDK11a
MGDEKDSWKVKTLDEILQEKKRRKEQEEKAEIKRLKNSDDRDSKRDSLEEGELRDHCMEITIRNSPYRREDSMEDRGEED
DSLAIKPPQQMSRKEKVHHRKDEKRKEKCRHHSHSAEGGKHARVKEREHERRKRHREEQDKARREWERQKRREMAREHSR
RERDRLEQLERKRERERKMREQQKEQREQKERERRAEERRKEREARREVSAHHRTMREDYSDKVKASHWSRSPPRPPRER
FELGDGRKPVKEEKMEERDLLSDLQDISDSERKTSSAESSSAESGSGSEEEEEEEEEEEEEGSTSEESEEEEEEEEEEEE
ETGSNSEEASEQSAEEVSEEEMSEDEERENENHLLVVPESRFDRDSGESEEAEEEVGEGTPQSSALTEGDYVPDSPALLP
IELKQELPKYLPALQGCRSVEEFQCLNRIEEGTYGVVYRAKDKKTDEIVALKRLKMEKEKEGFPITSLREINTILKAQHP
NIVTVREIVVGSNMDKIYIVMNYVEHDLKSLMETMKQPFLPGEVKTLMIQLLRGVKHLHDNWILHRDLKTSNLLLSHAGI
LKVGDFGLAREYGSPLKAYTPVVVTQWYRAPELLLGAKEYSTAVDMWSVGCIFGELLTQKPLFPGNSEIDQINKVFKELG
TPSEKIWPGYSELPVVKKMTFSEHPYNNLRKRFGALLSDQGFDLMNKFLTYFPGRRISAEDGLKHEYFRETPLPIDPSMF
PTWPAKSEQQRVKRGTSPRPPEGGLGYSQLGDDDLKETGFHLTTTNQGASAAGPGFSLKF
>Hsa-CDK20
MDQYCILGRIGEGAHGIVFKAKHVETGEIVALKKVALRRLEDGFPNQALREIKALQEMEDNQYVVQLKAVFPHGGGFVLA
FEFMLSDLAEVVRHAQRPLAQAQVKSYLQMLLKGVAFCHANNIVHRDLKPANLLISASGQLKIADFGLARVFSPDGSRLY
THQVATRWYRAPELLYGARQYDQGVDLWSVGCIMGELLNGSPLFPGKNDIEQLCYVLRILGTPNPQVWPELTELPDYNKI
SFKEQVPMPLEEVLPDVSPQALDLLGQFLLYPPHQRIAASKALLHQYFFTAPLPAHPSELPIPQRLGGPAPKAHPGPPHI
HDFHVDRPLEESLLNPELIRPFILEG
>Hsa-CDK11b
MGDEKDSWKVKTLDEILQEKKRRKEQEEKAEIKRLKNSDDRDSKRDSLEEGELRDHRMEITIRNSPYRREDSMEDRGEED
DSLAIKPPQQMSRKEKVHHRKDEKRKEKRRHRSHSAEGGKHARVKEKEREHERRKRHREEQDKARREWERQKRREMAREH
SRRERDRLEQLERKRERERKMREQQKEQREQKERERRAEERRKEREARREVSAHHRTMREDYSDKVKASHWSRSPPRPPR
ERFELGDGRKPVKEEKMEERDLLSDLQDISDSERKTSSAESSSAESGSGSEEEEEEEEEEEEEGSTSEESEEEEEEEEEE
EEETGSNSEEASEQSAEEVSEEEMSEDEERENENHLLVVPESRFDRDSGESEEAEEEVGEGTPQSSALTEGDYVPDSPAL
SPIELKQELPKYLPALQGCRSVEEFQCLNRIEEGTYGVVYRAKDKKTDEIVALKRLKMEKEKEGFPITSLREINTILKAQ
HPNIVTVREIVVGSNMDKIYIVMNYVEHDLKSLMETMKQPFLPGEVKTLMIQLLRGVKHLHDNWILHRDLKTSNLLLSHA
GILKVGDFGLAREYGSPLKAYTPVVVTLWYRAPELLLGAKEYSTAVDMWSVGCIFGELLTQKPLFPGKSEIDQINKVFKD
LGTPSEKIWPGYSELPAVKKMTFSEHPYNNLRKRFGALLSDQGFDLMNKFLTYFPGRRISAEDGLKHEYFRETPLPIDPS

MFPTWPAKSEQQRVKRGTSPRPPEGGLGYSQLGDDDLKETGFHLTTTNQGASAAGPGFSLKF
>Hsa-CDK10
MAEPDLECEQIRLKCIRKEGFFTVPPEHRLGRCRSVKEFEKLNRIGEGTYGIVYRARDTQTDEIVALKKVRMDKEKDGIP
ISSLREITLLLRLRHPNIVELKEVVVGNHLESIFLVMGYCEQDLASLLENMPTPFSEAQVKCIVLQVLRGLQYLHRNFII
HRDLKVSNLLMTDKGCVKTADFGLARAYGVPVKPMTPKVVTLWYRAPELLLGTTTQTTSIDMWAVGCILAELLAHRPLLP
GTSEIHQIDLIVQLLGTPSENIWPGFSKLPLVGQYSLRKQPYNNLKHKFPWLSEAGLRLLHFLFMYDPKKRATAGDCLES
SYFKEKPLPCEPELMPTFPHHRNKRAAPATSEGQSKRCKP
>Hsa-CDK16
MDRMKKIKRQLSMTLRGGRGIDKTNGAPEQIGLDESGGGGGSDPGEAPTRAAPGELRSARGPLSSAPEIVHEDLKMGSDG
ESDQASATSSDEVQSPVRVRMRNHPPRKISTEDINKRLSLPADIRLPEGYLEKLTLNSPIFDKPLSRRLRRVSLSEIGFG
KLETYIKLDKLGEGTYATVYKGKSKLTDNLVALKEIRLEHEEGAPCTAIREVSLLKDLKHANIVTLHDIIHTEKSLTLVF
EYLDKDLKQYLDDCGNIINMHNVKLFLFQLLRGLAYCHRQKVLHRDLKPQNLLINERGELKLADFGLARAKSIPTKTYSN
EVVTLWYRPPDILLGSTDYSTQIDMWGVGCIFYEMATGRPLFPGSTVEEQLHFIFRILGTPTEETWPGILSNEEFKTYNY
PKYRAEALLSHAPRLDSDGADLLTKLLQFEGRNRISAEDAMKHPFFLSLGERIHKLPDTTSIFALKEIQLQKEASLRSSS
MPDSGRPAFRVVDTEF
>Hsa-CDK14
MHGYFGCNAAAEPGYSAFVGTPQICVTKMSTRNCQGMDSVIKPLDTIPEDKKVRVQRTQSTFDPFEKPANQVKRVHSENN
ACINFKTSSTGKESPKVRRHSSPSSPTSPKFGKADSYEKLEKLGEGSYATVYKGKSKVNGKLVALKVIRLQEEEGTPFTA
IREASLLKGLKHANIVLLHDIIHTKETLTLVFEYVHTDLCQYMDKHPGGLHPDNVKLFLFQLLRGLSYIHQRYILHRDLK
PQNLLISDTGELKLADFGLARAKSVPSHTYSNEVVTLWYRPPDVLLGSTEYSTCLDMWGVGCIFVEMIQGVAAFPGMKDI
QDQLERIFLVLGTPNEDTWPGVHSLPHFKPERFTLYSSKNLRQAWNKLSYVNHAEDLASKLLQCSPKNRLSAQAALSHEY
FSDLPPRLWELTDMSSIFTVPNVRLQPEAGESMRAFGKNNSYGKSLSNSKH
>Hsa-CDK17
MKKFKRRLSLTLRGSQTIDESLSELAEQMTIEENSSKDNEPIVKNGRPPTSHSMHSFLHQYTGSFKKPPLRRPHSVIGGS
LGSFMAMPRNGSRLDIVHENLKMGSDGESDQASGTSSDEVQSPTGVCLRNRIHRRISMEDLNKRLSLPADIRIPDGYLEK
LQINSPPFDQPMSRRSRRASLSEIGFGKMETYIKLEKLGEGTYATVYKGRSKLTENLVALKEIRLEHEEGAPCTAIREVS
LLKDLKHANIVTLHDIVHTDKSLTLVFEYLDKDLKQYMDDCGNIMSMHNVKLFLYQILRGLAYCHRRKVLHRDLKPQNLL
INEKGELKLADFGLARAKSVPTKTYSNEVVTLWYRPPDVLLGSSEYSTQIDMWGVGCIFFEMASGRPLFPGSTVEDELHL
IFRLLGTPSQETWPGISSNEEFKNYNFPKYKPQPLINHAPRLDSEGIELITKFLQYESKKRVSAEEAMKHVYFRSLGPRI
HALPESVSIFSLKEIQLQKDPGFRNSSYPETGHGKNRRQSMLF
>Hsa-CDK18
MIMNKMKNFKRRFSLSVPRTETIEESLAEFTEQFNQLHNRRNENLQLGPLGRDPPQECSTFSPTDSGEEPGQLSPGVQFQ
RRQNQRRFSMEVRASGALPRQVAGCTHKGVHRRAAALQPDFDVSKRLSLPMDIRLPQEFLQKLQMESPDLPKPLSRMSRR
ASLSDIGFGKLETYVKLDKLGEGTYATVFKGRSKLTENLVALKEIRLEHEEGAPCTAIREVSLLKNLKHANIVTLHDLIH
TDRSLTLVFEYLDSDLKQYLDHCGNLMSMHNVKIFMFQLLRGLAYCHHRKILHRDLKPQNLLINERGELKLADFGLARAK
SVPTKTYSNEVVTLWYRPPDVLLGSTEYSTPIDMWGVGCIHYEMATGRPLFPGSTVKEELHLIFRLLGTPTEETWPGVTA
FSEFRTYSFPCYLPQPLINHAPRLDTDGIHLLSSLLLYESKSRMSAEAALSHSYFRSLGERVHQLEDTASIFSLKEIQLQ
KDPGYRGLAFQQPGRGKNRRQSIF
>Hsa-CDK15
MTSFHPRGLQAARAQKFKSKRPRSNSDCFQEEDLRQGFQWRKSLPFGAASSYLNLEKLGEGSYATVYKGISRINGQLVAL
KVISMNAEEGVPFTAIREASLLKGLKHANIVLLHDIIHTKETLTFVFEYMHTDLAQYMSQHPGGLHPHNVRLFMFQLLRG
LAYIHHQHVLHRDLKPQNLLISHLGELKLADFGLARAKSIPSQTYSSEVVTLWYRPPDALLGATEYSSELDIWGAGCIFI
EMFQGQPLFPGVSNILEQLEKIWEVLGVPTEDTWPGVSKLPNYNPEWFPLPTPRSLHVVWNRLGRVPEAEDLASQMLKGF
PRDRVSAQEALVHDYFSALPSQLYQLPDEESLFTVSGVRLKPEMCDLLASYQKGHHPAQFSKCW
>Hsa-CDK9
MAKQYDSVECPFCDEVSKYEKLAKIGQGTFGEVFKARHRKTGQKVALKKVLMENEKEGFPITALREIKILQLLKHENVVN
LIEICRTKASPYNRCKGSIYLVFDFCEHDLAGLLSNVLVKFTLSEIKRVMQMLLNGLYYIHRNKILHRDMKAANVLITRD
GVLKLADFGLARAFSLAKNSQPNRYTNRVVTLWYRPPELLLGERDYGPPIDLWGAGCIMAEMWTRSPIMQGNTEQHQLAL
ISQLCGSITPEVWPNVDNYELYEKLELVKGQKRKVKDRLKAYVRDPYALDLIDKLLVLDPAQRIDSDDALNHDFFWSDPM
PSDLKGMLSTHLTSMFEYLAPPRRKGSQITQQSTNQSRNPATTNQTEFERVF
>Hsa-CDK13
MPSSSDTALGGGGGLSWAEKKLEERRKRRRFLSPQQPPLLLPLLQPQLLQPPPPPPPLLFLAAPGTAAAAAAAAAASSSC
FSPGPPLEVKRLARGKRRAGGRQKRRRGPRAGQEAEKRRVFSLPQPQQDGGGGASSGGGVTPLVEYEDVSSQSEQGLLLG
GASAATAATAAGGTGGSGGSPASSSGTQRRGEGSERRPRRDRRSSSGRSKERHREHRRRDGQRGGSEASKSRSRHSHSGE
ERAEVAKSGSSSSSGGRRKSASATSSSSSSRKDRDSKAHRSRTKSSKEPPSAYKEPPKAYREDKTEPKAYRRRRSLSPLG
GRDDSPVSHRASQSLRSRKSPSPAGGGSSPYSRRLPRSPSPYSRRRSPSYSRHSSYERGGDVSPSPYSSSSWRRSRSPYS
PVLRRSGKSRSRSPYSSRHSRSRSRHRLSRSRSRHSSISPSTLTLKSSLAAELNKNKKARAAEAARAAEAAKAAEATKAA

EAAAKAAKASNTSTPTKGNTETSASASQTNHVKDVKKIKIEHAPSPSSGGTLKNDKAKTKPPLQVTKVENNLIVDKATKK
AVIVGKESKSAATKEESVSLKEKTKPLTPSIGAKEKEQHVALVTSTLPPLPLPPMLPEDKEADSLRGNISVKAVKKEVEK
KLRCLLADLPLPPELPGGDDLSKSPEEKKTATQLHSKRRPKICGPRYGETKEKDIDWGKRCVDKFDIIGIIGEGTYGQVY
KARDKDTGEMVALKKVRLDNEKEGFPITAIREIKILRQLTHQSIINMKEIVTDKEDALDFKKDKGAFYLVFEYMDHDLMG
LLESGLVHFNENHIKSFMRQLMEGLDYCHKKNFLHRDIKCSNILLNNRGQIKLADFGLARLYSSEESRPYTNKVITLWYR
PPELLLGEERYTPAIDVWSCGCILGELFTKKPIFQANQELAQLELISRICGSPCPAVWPDVIKLPYFNTMKPKKQYRRKL
REEFVFIPAAALDLFDYMLALDPSKRCTAEQALQCEFLRDVEPSKMPPPDLPLWQDCHELWSKKRRRQKQMGMTDDVSTI
KAPRKDLSLGLDDSRTNTPQGVLPSSQLKSQGSSNVAPVKTGPGQHLNHSELAILLNLLQSKTSVNMADFVQVLNIKVNS
ETQQQLNKINLPAGILATGEKQTDPSTPQQESSKPLGGIQPSSQTIQPKVETDAAQAAVQSAFAVLLTQLIKAQQSKQKD
VLLEERENGSGHEASLQLRPPPEPSTPVSGQDDLIQHQDMRILELTPEPDRPRILPPDQRPPEPPEPPPVTEEDLDYRTE
NQHVPTTSSSLTDPHAGVKAALLQLLAQHQPQDDPKREGGIDYQAGDTYVSTSDYKDNFGSSSFSSAPYVSNDGLGSSSA
PPLERRSFIGNSDIQSLDNYSTASSHSGGPPQPSAFSESFPSSVAGYGDIYLNAGPMLFSGDKDHRFEYSHGPIAVLANS
SDPSTGPESTHPLPAKMHNYNYGGNLQENPSGPSLMHGQTWTSPAQGPGYSQGYRGHISTSTGRGRGRGLPY
>Hsa-CDK12
MPNSERHGGKKDGSGGASGTLQPSSGGGSSNSRERHRLVSKHKRHKSKHSKDMGLVTPEAASLGTVIKPLVEYDDISSDS
DTFSDDMAFKLDRRENDERRGSDRSDRLHKHRHHQHRRSRDLLKAKQTEKEKSQEVSSKSGSMKDRISGSSKRSNEETDD
YGKAQVAKSSSKESRSSKLHKEKTRKERELKSGHKDRSKSHRKRETPKSYKTVDSPKRRSRSPHRKWSDSSKQDDSPSGA
SYGQDYDLSPSRSHTSSNYDSYKKSPGSTSRRQSVSPPYKEPSAYQSSTRSPSPYSRRQRSVSPYSRRRSSSYERSGSYS
GRSPSPYGRRRSSSPFLSKRSLSRSPLPSRKSMKSRSRSPAYSRHSSSHSKKKRSSSRSRHSSISPVRLPLNSSLGAELS
RKKKERAAAAAAAKMDGKESKGSPVFLPRKENSSVEAKDSGLESKKLPRSVKLEKSAPDTELVNVTHLNTEVKNSSDTGK
VKLDENSEKHLVKDLKAQGTRDSKPIALKEEIVTPKETETSEKETPPPLPTIASPPPPLPTTTPPPQTPPLPPLPPIPAL
PQQPPLPPSQPAFSQVPASSTSTLPPSTHSKTSAVSSQANSQPPVQVSVKTQVSVTAAIPHLKTSTLPPLPLPPLLPGDD
DMDSPKETLPSKPVKKEKEQRTRHLLTDLPLPPELPGGDLSPPDSPEPKAITPPQQPYKKRPKICCPRYGERRQTESDWG
KRCVDKFDIIGIIGEGTYGQVYKAKDKDTGELVALKKVRLDNEKEGFPITAIREIKILRQLIHRSVVNMKEIVTDKQDAL
DFKKDKGAFYLVFEYMDHDLMGLLESGLVHFSEDHIKSFMKQLMEGLEYCHKKNFLHRDIKCSNILLNNSGQIKLADFGL
ARLYNSEESRPYTNKVITLWYRPPELLLGEERYTPAIDVWSCGCILGELFTKKPIFQANLELAQLELISRLCGSPCPAVW
PDVIKLPYFNTMKPKKQYRRRLREEFSFIPSAALDLLDHMLTLDPSKRCTAEQTLQSDFLKDVELSKMAPPDLPHWQDCH
ELWSKKRRRQRQSGVVVEEPPPSKTSRKETTSGTSTEPVKNSSPAPPQPAPGKVESGAGDAIGLADITQQLNQSELAVLL
NLLQSQTDLSIPQMAQLLNIHSNPEMQQQLEALNQSISALTEATSQQQDSETMAPEESLKEAPSAPVILPSAEQTTLEAS
STPADMQNILAVLLSQLMKTQEPAGSLEENNSDKNSGPQGPRRTPTMPQEEAAACPPHILPPEKRPPEPPGPPPPPPPPP
LVEGDLSSAPQELNPAVTAALLQLLSQPEAEPPGHLPHEHQALRPMEYSTRPRPNRTYGNTDGPETGFSAIDTDERNSGP
ALTESLVQTLVKNRTFSGSLSHLGESSSYQGTGSVQFPGDQDLRFARVPLALHPVVGQPFLKAEGSSNSVVHAETKLQNY
GELGPGTTGASSSGAGLHWGGPTQSSAYGKLYRGPTRVPPRGGRGRGVPY
>Hsa-CDK6
MEKDGLCRADQQYECVAEIGEGAYGKVFKARDLKNGGRFVALKRVRVQTGEEGMPLSTIREVAVLRHLETFEHPNVVRLF
DVCTVSRTDRETKLTLVFEHVDQDLTTYLDKVPEPGVPTETIKDMMFQLLRGLDFLHSHRVVHRDLKPQNILVTSSGQIK
LADFGLARIYSFQMALTSVVVTLWYRAPEVLLQSSYATPVDLWSVGCIFAEMFRRKPLFRGSSDVDQLGKILDVIGLPGE
EDWPRDVALPRQAFHSKSAQPIEKFVTDIDELGKDLLLKCLTFNPAKRISAYSALSHPYFQDLERCKENLDSHLPPSQNT
SELNTA
>Hsa-CDK19
MDYDFKAKLAAERERVEDLFEYEGCKVGRGTYGHVYKARRKDGKDEKEYALKQIEGTGISMSACREIALLRELKHPNVIA
LQKVFLSHSDRKVWLLFDYAEHDLWHIIKFHRASKANKKPMQLPRSMVKSLLYQILDGIHYLHANWVLHRDLKPANILVM
GEGPERGRVKIADMGFARLFNSPLKPLADLDPVVVTFWYRAPELLLGARHYTKAIDIWAIGCIFAELLTSEPIFHCRQED
IKTSNPFHHDQLDRIFSVMGFPADKDWEDIRKMPEYPTLQKDFRRTTYANSSLIKYMEKHKVKPDSKVFLLLQKLLTMDP
TKRITSEQALQDPYFQEDPLPTLDVFAGCQIPYPKREFLNEDDPEEKGDKNQQQQQNQHQQPTAPPQQAAAPPQAPPPQQ
NSTQTNGTAGGAGAGVGGTGAGLQHSQDSSLNQVPPNKKPRLGPSGANSGGPVMPSDYQHSSSRLNYQSSVQGSSQSQST
LGYSSSSQQSSQYHPSHQAHRY
>Hsa-CDK8
MDYDFKVKLSSERERVEDLFEYEGCKVGRGTYGHVYKAKRKDGKDDKDYALKQIEGTGIS
MSACREIALLRELKHPNVISLQKVFLSHADRKVWLLFDYAEHDLWHIIKFHRASKANKKP
VQLPRGMVKSLLYQILDGIHYLHANWVLHRDLKPANILVMGEGPERGRVKIADMGFARLF
NSPLKPLADLDPVVVTFWYRAPELLLGARHYTKAIDIWAIGCIFAELLTSEPIFHCRQED
IKTSNPYHHDQLDRIFNVMGFPADKDWEDIKKMPEHSTLMKDFRRNTYTNCSLIKYMEKH
KVKPDSKAFHLLQKLLTMDPIKRITSEQAMQDPYFLEDPLPTSDVFAGCQIPYPKREFLT
EEEPDDKGDKKNQQQQQGNNHTNGTGHPGNQDSSHTQGPPLKKVRVVPPTTTSGGLIMTS
DYQRSNPHAAYPNPGPSTSQPQSSMGYSATSQQPPQYSHQTHRY
>Hsa-CDK4

MATSRYEPVAEIGVGAYGTVYKARDPHSGHFVALKSVRVPNGGGGGGGLPISTVREVALLRRLEAFEHPNVVRLMDVCAT
SRTDREIKVTLVFEHVDQDLRTYLDKAPPPGLPAETIKDLMRQFLRGLDFLHANCIVHRDLKPENILVTSGGTVKLADFG
LARIYSYQMALTPVVVTLWYRAPEVLLQSTYATPVDMWSVGCIFAEMFRRKPLFCGNSEADQLGKIFDLIGLPPEDDWPR
DVSLPRGAFPPRGPRPVQSVVPEMEESGAQLLLEMLTFNPHKRISAFRALQHSYLHKDEGNPE
>Hsa-CDKL1
MMEKYEKIGKIGEGSYGVVFKCRNRDTGQIVAIKKFLESEDDPVIKKIALREIRMLKQLKHPNLVNLLEVFRRKRRLHLV
FEYCDHTVLHELDRYQRGVPEHLVKSITWQTLQAVNFCHKHNCIHRDVKPENILITKHSVIKLCDFGFARLLTGPSDYYT
DYVATRWYRSPELLVGDTQYGPPVDVWAIGCVFAELLSGVPLWPGKSDVDQLYLIRKTLGDLIPRHQQVFSTNQYFSGVK
IPDPEDMEPLELKFPNISYPALGLLKGCLHMDPTQRLTCEQLLHHPYFENIREIEDLAKEHNKPTRKTLRKSRKHHCFTE
TSKLQYLPQLTGSSILPALDNKKYYCDTKKLNYRFPNI
>Hsa-GSK3alpha
MSGGGPSGGGPGGSGRARTSSFAEPGGGGGGGGGGPGGSASGPGGTGGGKASVGAMGGGVGASSSGGGPGGSGGGGS
GGP
GAGTSFPPPGVKLGRDSGKVTTVVATLGQGPERSQEVAYTDIKVIGNGSFGVVYQARLAETRELVAIKKVLQDKRFKNRE
LQIMRKLDHCNIVRLRYFFYSSGEKKDELYLNLVLEYVPETVYRVARHFTKAKLTIPILYVKVYMYQLFRSLAYIHSQGV
CHRDIKPQNLLVDPDTAVLKLCDFGSAKQLVRGEPNVSYICSRYYRAPELIFGATDYTSSIDVWSAGCVLAELLLGQPIF
PGDSGVDQLVEIIKVLGTPTREQIREMNPNYTEFKFPQIKAHPWTKVFKSRTPPEAIALCSSLLEYTPSSRLSPLEACAH
SFFDELRCLGTQLPNNRPLPPLFNFSAGELSIQPSLNAILIPPHLRSPAGTTTLTPSSQALTETPTSSDWQSTDATPTLT
NSS
>Hsa-MAK
MNRYTTMRQLGDGTYGSVLMGKSNESGELVAIKRMKRKFYSWDECMNLREVKSLKKLNHANVIKLKEVIRENDHLYFIFE
YMKENLYQLMKDRNKLFPESVIRNIMYQILQGLAFIHKHGFFHRDMKPENLLCMGPELVKIADFGLARELRSQPPYTDYV
STRWYRAPEVLLRSSVYSSPIDVWAVGSIMAELYMLRPLFPGTSEVDEIFKICQVLGTPKKSDWPEGYQLASSMNFRFPQ
CVPINLKTLIPNASNEAIQLMTEMLNWDPKKRPTASQALKHPYFQVGQVLGPSSNHLESKQSLNKQLQPLESKPSLVEVE
PKPLPDIIDQVVGQPQPKTSQQPLQPIQPPQNLSVQQPPKQQSQEKPPQTLFPSIVKNMPTKPNGTLSHKSGRRRWGQTI
FKSGDSWEELEDYDFGASHSKKPSMGVFKEKRKKDSPFRLPEPVPSGSNHSTGENKSLPAVTSLKSDSELSTAPTSKQYY
LKQSRYLPGVNPKKVSLIASGKEINPHTWSNQLFPKSLGPVGAELAFKRSNAGNLGSYATYNQSGYIPSFLKKEVQSAGQ
RIHLAPLNATASEYTWNTKTGRGQFSGRTYNPTAKNLNIVNRAQPIPSVHGRTDWVAKYGGHR
