# Supplementary material for: Roadmap to the study of gene and protein phylogeny and evolution—A practical guide
Source: PLoS One. 2023 Feb 24;18(2):e0279597. doi: 10.1371/journal.pone.0279597 (PMC9955684; doi:10.1371/journal.pone.0279597)
Supplement: S4 File — The coding was used to reconstruct the co-phylogeny of human CDKs and Cyclins. (PDF) [file pone.0279597.s004.pdf]

SI File 3 (SI F3). Sequences of human cyclins which were used for phylogenetic analysis.

>Hsa-CyclinA1
METGFPAIMYPGSFIGGWGEEYLSWEGPGLPDFVFQQQPVESEAMHCSNPKSGVVLATVARGPDACQILT
RAPLGQDPPQRTVLGLLTANGQYRRTCGQGITRIRCYSGSENAFPPAGKKALPDCGVQEPPKQGFDIYMD
ELEQGDRDSCSVREGMAFEDVYEVDTGTLKSDLHFLLDFNTVSPMLVDSSLLSQSEDISSLGTDVINVTE
YAEEIYQYLREAEIRHRPKAHYMKKQPDITEGMRTILVDWLVEVGEEYKLRAETLYLAVNFLDRFLSCMS
VLRGKLQLVGTAAMLLASKYEEIYPPEVDEFVYITDDTYTKRQLLKMEHLLLKVLAFDLTVPTTNQFLLQ
YLRRQGVCVRTENLAKYVAELSLLEADPFLKYLPSLIAAAAFCLANYTVNKHFWPETLAAFTGYSLSEIV
PCLSELHKAYLDIPHRPQQAIREKYKASKYLCVSLMEPPAVLLLQ
>Hsa-CyclinA2
MLGNSAPGPATREAGSALLALQQTALQEDQENINPEKAAPVQQPRTRAALAVLKSGNPRGLAQQQRPKTR
RVAPLKDLPVNDEHVTVPPWKANSKQPAFTIHVDEAEKEAQKKPAESQKIEREDALAFNSAISLPGPRKP
LVPLDYPMDGSFESPHTMDMSIVLEDEKPVSVNEVPDYHEDIHTYLREMEVKCKPKVGYMKKQPDITNSM
RAILVDWLVEVGEEYKLQNETLHLAVNYIDRFLSSMSVLRGKLQLVGTAAMLLASKFEEIYPPEVAEFVY
ITDDTYTKKQVLRMEHLVLKVLTFDLAAPTVNQFLTQYFLHQQPANCKVESLAMFLGELSLIDADPYLKY
LPSVIAGAAFHLALYTVTGQSWPESLIRKTGYTLESLKPCLMDLHQTYLKAPQHAQQSIREKYKNSKYHG
VSLLNPPETLNL
>Hsa-CyclinB2
MALLRRPTVSSDLENIDTGVNSKVKSHVTIRRTVLEEIGNRVTTRAAQVAKKAQNTKVPVQPTKTTNVNK
QLKPTASVKPVQMEKLAPKGPSPTPEDVSMKEENLCQAFSDALLCKIEDIDNEDWENPQLCSDYVKDIYQ
YLRQLEVLQSINPHFLDGRDINGRMRAILVDWLVQVHSKFRLLQETLYMCVGIMDRFLQVQPVSRKKLQL
VGITALLLASKYEEMFSPNIEDFVYITDNAYTSSQIREMETLILKELKFELGRPLPLHFLRRASKAGEVD
VEQHTLAKYLMELTLIDYDMVHYHPSKVAAAASCLSQKVLGQGKWNLKQQYYTGYTENEVLEVMQHMAKN
VVKVNENLTKFIAIKNKYASSKLLKISMIPQLNSKAVKDLASPLIGRS
>Hsa-CyclinB1
MALRVTRNSKINAENKAKINMAGAKRVPTAPAATSKPGLRPRTALGDIGNKVSEQLQAKMPMKKEAKPSA
TGKVIDKKLPKPLEKVPMLVPVPVSEPVPEPEPEPEPEPVKEEKLSPEPILVDTASPSPMETSGCAPAEE
DLCQAFSDVILAVNDVDAEDGADPNLCSEYVKDIYAYLRQLEEEQAVRPKYLLGREVTGNMRAILIDWLV
QVQMKFRLLQETMYMTVSIIDRFMQNNCVPKKMLQLVGVTAMFIASKYEEMYPPEIGDFAFVTDNTYTKH
QIRQMEMKILRALNFGLGRPLPLHFLRRASKIGEVDVEQHTLAKYLMELTMLDYDMVHFPPSQIAAGAFC
LALKILDNGEWTPTLQHYLSYTEESLLPVMQHLAKNVVMVNQGLTKHMTVKNKYATSKHAKISTLPQLNS
ALVQDLAKAVAKV
>Hsa-CyclinD3
MELLCCEGTRHAPRAGPDPRLLGDQRVLQSLLRLEERYVPRASYFQCVQREIKPHMRKMLAYWMLEVCEE
QRCEEEVFPLAMNYLDRYLSCVPTRKAQLQLLGAVCMLLASKLRETTPLTIEKLCIYTDHAVSPRQLRDW
EVLVLGKLKWDLAAVIAHDFLAFILHRLSLPRDRQALVKKHAQTFLALCATDYTFAMYPPSMIATGSIGA
AVQGLGACSMSGDELTELLAGITGTEVDCLRACQEQIEAALRESLREASQTSSSPAPKAPRGSSSQGPSQ
TSTPTDVTAIHL
>Hsa-CyclinB3
MLLPLPPQSSKPVPKKSQSSKIVPSHHDPSEKTGENCQTKISPSSLQESPSSLQGALKKRSAFEDLTNAS
QCQPVQPKKEANKEFVKVVSKKINRNTHALGLAKKNKRNLKWHKLEVTPVVASTTVVPNIMEKPLILDIS
TTSKTPNTEEASLFRKPLVLKEEPTIEDETLINKSLSLKKCSNHEEVSLLEKLQPLQEESDSDDAFVIEP
MTFKKTHKTEEAAITKKTLSLKKKMCASQRKQSCQEESLAVQDVNMEEDSFFMESMSFKKKPKTEESIPT
HKLSSLKKKCTIYGKICHFRKPPVLQTTICGAMSSIKKPTTEKETLFQELSVLQEKHTTEHEMSILKKSL
ALQKTNFKEDSLVKESLAFKKKPSTEEAIMMPVILKEQCMTEGKRSRLKPLVLQEITSGEKSLIMKPLSI
KEKPSTEKESFSQEPSALQKKHTTQEEVSILKEPSSLLKSPTEESPFDEALAFTKKCTIEEAPPTKKPLI
LKRKHATQGTMSHLKKPLILQTTSGEKSLIKEPLPFKEEKVSLKKKCTTQEMMSICPELLDFQDMIGEDK
NSFFMEPMSFRKNPTTEETVLTKTSLSLQEKKITQGKMSHLKKPLVLQKITSEEESFYKKLLPFKMKSTT
EEKFLSQEPSALKEKHTTLQEVSLSKESLAIQEKATTEEEFSQELFSLHVKHTNKSGSLFQEALVLQEKT
DAEEDSLKNLLALQEKSTMEEESLINKLLALKEELSAEAATNIQTQLSLKKKSTSHGKVFFLKKQLALNE
TINEEEFLNKQPLALEGYPSIAEGETLFKKLLAMQEEPSIEKEAVLKEPTIDTEAHFKEPLALQEEPSTE
KEAVLKEPSVDTEAHFKETLALQEKPSIEQEALFKRHSALWEKPSTEKETIFKESLDLQEKPSIKKETLL
KKPLALKMSTINEAVLFEDMIALNEKPTTGKELSFKEPLALQESPTYKEDTFLKTLLVPQVGTSPNVSST
APESITSKSSIATMTSVGKSGTINEAFLFEDMITLNEKPTTGKELSFKEPLALQESPTCKEDTFLETFLI

PQIGTSPYVFSTTPESITEKSSIATMTSVGKSRTTTESSACESASDKPVSPQAKGTPKEITPREDIDEDS
SDPSFNPMYAKEIFSYMKEREEQFILTDYMNRQIEITSDMRAILVDWLVEVQVSFEMTHETLYLAVKLVD
LYLMKAVCKKDKLQLLGATAFMIAAKFEEHNSPRVDDFVYICDDNYQRSEVLSMEINILNVLKCDINIPI
AYHFLRRYARCIHTNMKTLTLSRYICEMTLQEYHYVQEKASKLAAASLLLALYMKKLGYWVPFLEHYSGY
SISELHPLVRQLNKLLTFSSYDSLKAVYYKYSHPVFFEVAKIPALDMLKLEEILNCDCEAQGLVL
>Hsa-cyclinD2
MELLCHEVDPVRRAVRDRNLLRDDRVLQNLLTIEERYLPQCSYFKCVQKDIQPYMRRMVA
TWMLEVCEEQKCEEEVFPLAMNYLDRFLAGVPTPKSHLQLLGAVCMFLASKLKETSPLTA
EKLCIYTDNSIKPQELLEWELVVLGKLKWNLAAVTPHDFIEHILRKLPQQREKLSLIRKH
AQTFIALCATDFKFAMYPPSMIATGSVGAAICGLQQDEEVSSLTCDALTELLAKITNTDV
DCLKACQEQIEAVLLNSLQQYRQDQRDGSKSEDELDQASTPTDVRDIDL
>Hsa-CyclinE1
MPRERRERDAKERDTMKEDGGAEFSARSRKRKANVTVFLQDPDEEMAKIDRTARDQCGSQPWDNNAVCAD
PCSLIPTPDKEDDDRVYPNSTCKPRIIAPSRGSPLPVLSWANREEVWKIMLNKEKTYLRDQHFLEQHPLL
QPKMRAILLDWLMEVCEVYKLHRETFYLAQDFFDRYMATQENVVKTLLQLIGISSLFIAAKLEEIYPPKL
HQFAYVTDGACSGDEILTMELMIMKALKWRLSPLTIVSWLNVYMQVAYLNDLHEVLLPQYPQQIFIQIAE
LLDLCVLDVDCLEFPYGILAASALYHFSSSELMQKVSGYQWCDIENCVKWMVPFAMVIRETGSSKLKHFR
GVADEDAHNIQTHRDSLDLLDKARAKKAMLSEQNRASPLPSGLLTPPQSGKKQSSGPEMA
>Hsa-CyclinE2
MSRRSSRLQAKQQPQPSQTESPQEAQIIQAKKRKTTQDVKKRREEVTKKHQYEIRNCWPPVLSGGISPCI
IIETPHKEIGTSDFSRFTNYRFKNLFINPSPLPDLSWGCSKEVWLNMLKKESRYVHDKHFEVLHSDLEPQ
MRSILLDWLLEVCEVYTLHRETFYLAQDFFDRFMLTQKDINKNMLQLIGITSLFIASKLEEIYAPKLQEF
AYVTDGACSEEDILRMELIILKALKWELCPVTIISWLNLFLQVDALKDAPKVLLPQYSQETFIQIAQLLD
LCILAIDSLEFQYRILTAAALCHFTSIEVVKKASGLEWDSISECVDWMVPFVNVVKSTSPVKLKTFKKIP
MEDRHNIQTHTNYLAMLEEVNYINTFRKGGQLSPVCNGGIMTPPKSTEKPPGKH
>Hsa-CyclinK
MKENKENSSPSVTSANLDHTKPCWYWDKKDLAHTPSQLEGLDPATEARYRREGARFIFDVGTRLGLHYDT
LATGIIYFHRFYMFHSFKQFPRYVTGACCLFLAGKVEETPKKCKDIIKTARSLLNDVQFGQFGDDPKEEV
MVLERILLQTIKFDLQVEHPYQFLLKYAKQLKGDKNKIQKLVQMAWTFVNDSLCTTLSLQWEPEIIAVAV
MYLAGRLCKFEIQEWTSKPMYRRWWEQFVQDVPVDVLEDICHQILDLYSQGKQQMPHHTPHQLQQPPSLQ
PTPQVPQVQQSQPSQSSEPSQPQQKDPQQPAQQQQPAQQPKKPSPQPSSPRQVKRAVVVSPKEENKAAEP
PPPKIPKIETTHPPLPPAHPPPDRKPPLAAALGEAEPPGPVDATDLPKVQIPPPAHPAPVHQPPPLPHRP
PPPPPSSYMTGMSTTSSYMSGEGYQSLQSMMKTEGPSYGALPPAYGPPAHLPYHPHVYPPNPPPPPVPPP
PASFPPPAIPPPTPGYPPPPPTYNPNFPPPPPRLPPTHAVPPHPPPGLGLPPASYPPPAVPPGGQPPVPP
PIPPPGMPPVGGLGRAAWMR
>Hsa-CyclinJ
MELEGQWWRGQLAADIHQALRYKELKLPSYKGQSPQLSLRRYFADLIAIVSNRFTLCPSARHLAVYLLDL
FMDRYDISIQQLHLVALSCLLLASKFEEKEDSVPKLEQLNSLGCMTNMNLVLTKQNLLHMELLLLETFQW
NLCLPTAAHFIEYYLSEAVHETDLHDGWPMICLEKTKLYMAKYADYFLEVSLQDYAFLNYAPSLVAAACV
ASSRIILRLSPTWPTRLHRLTAYSWDFLVQCIERLLIAHDNDVKEANKQRGQAGPQSAQLSVFQTASQPS
RPVHFQQPQYLHQTHQTSLQYRHPTSEQPSCQQIVSTTHTSSYTLQTCPAGFQTSVQGLGHMQTGVGMSL
AIPVEVKPCLSVSYNRSYQINEHYPCITPCFER
>Hsa-CyclinF
MGSGGVVHCRCAKCFCYPTKRRIRRRPRNLTILSLPEDVLFHILKWLSVEDILAVRAVHSQLKDLVDNHA
SVWACASFQELWPSPGNLKLFERAAEKGNFEAAVKLGIAYLYNEGLSVSDEARAEVNGLKASRFFSLAER
LNVGAAPFIWLFIRPPWSVSGSCCKAVVHESLRAECQLQRTHKASILHCLGRVLSLFEDEEKQQQAHDLF
EEAAHQGCLTSSYLLWESDRRTDVSDPGRCLHSFRKLRDYAAKGCWEAQLSLAKACANANQLGLEVRASS
EIVCQLFQASQAVSKQQVFSVQKGLNDTMRYILIDWLVEVATMKDFTSLCLHLTVECVDRYLRRRLVPRY
RLQLLGIACMVICTRFISKEILTIREAVWLTDNTYKYEDLVRMMGEIVSALEGKIRVPTVVDYKEVLLTL
VPVELRTQHLCSFLCELSLLHTSLSAYAPARLAAAALLLARLTHGQTQPWTTQLWDLTGFSYEDLIPCVL
SLHKKCFHDDAPKDYRQVSLTAVKQRFEDKRYGEISQEEVLSYSQLCAALGVTQDSPDPPTFLSTGEIHA
FLSSPSGRRTKRKRENSLQEDRGSFVTTPTAELSSQEETLLGSFLDWSLDCCSGYEGDQESEGEKEGDVT
APSGILDVTVVYLNPEQHCCQESSDEEACPEDKGPQDPQALALDTQIPATPGPKPLVRTSREPGKDVTTS
GYSSVSTASPTSSVDGGLGALPQPTSVLSLDSDSHTQPCHHQARKSCLQCRPPSPPESSVPQQQVKRINL
CIHSEEEDMNLGLVRL
>Hsa-CyclinO
MVTPCPTSPSSPAARAGRRDNDQNLRAPVKKSRRPRLRRKQPLHPLNPCPLPGDSGICDLFESPSSGSDG

AESPSAARGGSPLPGPAQPVAQLDLQTFRDYGQSCYAFRKAQESHFHPREALARQPQVTAESRCKLLSWL
IPVHRQFGLSFESLCLTVNTLDRFLTTTPVAADCFQLLGVTSLLIACKQVEVHPPRVKQLLALCCGAFSR
QQLCNLECIVLHKLHFTLGAPTISFFLEHFTHARVEAGQAEASEALEAQALARGVAELSLADYAFTSYSP
SLLAICCLALADRMLRVSRPVDLRLGDHPEAALEDCMGKLQLLVAINSTSLTHMLPVQICEKCSLPPSSK
>Hsa-CyclinD1
MEHQLLCCEVETIRRAYPDANLLNDRVLRAMLKAEETCAPSVSYFKCVQKEVLPSMRKIVATWMLEVCEE
QKCEEEVFPLAMNYLDRFLSLEPVKKSRLQLLGATCMFVASKMKETIPLTAEKLCIYTDNSIRPEELLQM
ELLLVNKLKWNLAAMTPHDFIEHFLSKMPEAEENKQIIRKHAQTFVALCATDVKFISNPPSMVAAGSVVA
AVQGLNLRSPNNFLSYYRLTRFLSRVIKCDPDCLRACQEQIEALLESSLRQAQQNMDPKAAEEEEEEEEE
VDLACTPTDVRDVDI
>Hsa-CyclinT2
MASGRGASSRWFFTREQLENTPSRRCGVEADKELSCRQQAANLIQEMGQRLNVSQLTINTAIVYMHRFYM
HHSFTKFNKNIISSTALFLAAKVEEQARKLEHVIKVAHACLHPLEPLLDTKCDAYLQQTQELVILETIML
QTLGFEITIEHPHTDVVKCTQLVRASKDLAQTSYFMATNSLHLTTFCLQYKPTVIACVCIHLACKWSNWE
IPVSTDGKHWWEYVDPTVTLELLDELTHEFLQILEKTPNRLKKIRNWRANQAARKPKVDGQVSETPLLGS
SLVQNSILVDSVTGVPTNPSFQKPSTSAFPAPVPLNSGNISVQDSHTSDNLSMLATGMPSTSYGLSSHQE
WPQHQDSARTEQLYSQKQETSLSGSQYNINFQQGPSISLHSGLHHRPDKISDHSSVKQEYTHKAGSSKHH
GPISTTPGIIPQKMSLDKYREKRKLETLDLDVRDHYIAAQVEQQHKQGQSQAASSSSVTSPIKMKIPIAN
TEKYMADKKEKSGSLKLRIPIPPTDKSASKEELKMKIKVSSSERHSSSDEGSGKSKHSSPHISRDHKEKH
KEHPSSRHHTSSHKHSHSHSGSSSGGSKHSADGIPPTVLRSPVGLSSDGISSSSSSSRKRLHVNDASHNH
HSKMSKSSKSSGGLRTSQHPRETGQEASGDQRS
>Hsa-CyclinL2
MAAAAAAAGAAGSAAPAAAAGAPGSGGAPSGSQGVLIGDRLYSGVLITLENCLLPDDKLRFTPSMSSGLD
TDTETDLRVVGCELIQAAGILLRLPQVAMATGQVLFQRFFYTKSFVKHSMEHVSMACVHLASKIEEAPRR
IRDVINVFHRLRQLRDKKKPVPLLLDQDYVNLKNQIIKAERRVLKELGFCVHVKHPHKIIVMYLQVLECE
RNQHLVQTSWNYMNDSLRTDVFVRFQPESIACACIYLAARTLEIPLPNRPHWFLLFGATEEEIQEICLKI
LQLYARKKVDLTHLEGEVEKRKHAIEEAKAQARGLLPGGTQVLDGTSGFSPAPKLVESPKEGKGSKPSPL
SVKNTKRRLEGAKKAKADSPVNGLPKGRESRSRSRSREQSYSRSPSRSASPKRRKSDSGSTSGGSKSQSR
SRSRSDSPPRQAPRSAPYKGSEIRGSRKSKDCKYPQKPHKSRSRSSSRSRSRSRERADNPGKYKKKSHYY
RDQRRERSRSYERTGRRYERDHPGHSRHRR
>Hsa-CyclinG2
MKDLGAEHLAGHEGVQLLGLLNVYLEQEERFQPREKGLSLIEATPENDNTLCPGLRNAKVEDLRSLANFF
GSCTETFVLAVNILDRFLALMKVKPKHLSCIGVCSFLLAARIVEEDCNIPSTHDVIRISQCKCTASDIKR
MEKIISEKLHYELEATTALNFLHLYHTIILCHTSERKEILSLDKLEAQLKACNCRLIFSKAKPSVLALCL
LNLEVETLKSVELLEILLLVKKHSKINDTEFFYWRELVSKCLAEYSSPECCKPDLKKLVWIVSRRTAQNL
HNSYYSVPELPTIPEGGCFDESESEDSCEDMSCGEESLSSSPPSDQECTFFFNFKVAQTLCFPS
>Hsa-CyclinT1
MEGERKNNNKRWYFTREQLENSPSRRFGVDPDKELSYRQQAANLLQDMGQRLNVSQLTINTAIVYMHRFY
MIQSFTQFPGNSVAPAALFLAAKVEEQPKKLEHVIKVAHTCLHPQESLPDTRSEAYLQQVQDLVILESII
LQTLGFELTIDHPHTHVVKCTQLVRASKDLAQTSYFMATNSLHLTTFSLQYTPPVVACVCIHLACKWSNW
EIPVSTDGKHWWEYVDATVTLELLDELTHEFLQILEKTPNRLKRIWNWRACEAAKKTKADDRGTDEKTSE
QTILNMISQSSSDTTIAGLMSMSTSTTSAVPSLPVSEESSSNLTSVEMLPGKRWLSSQPSFKLEPTQGHR
TSENLALTGVDHSLPQDGSNAFISQKQNSKSVPSAKVSLKEYRAKHAEELAAQKRQLENMEANVKSQYAY
AAQNLLSHHDSHSSVILKMPIEGSENPERPFLEKADKTALKMRIPVAGGDKAASSKPEEIKMRIKVHAAA
DKHNSVEDSVTKSREHKEKHKTHPSNHHHHHNHHSHKHSHSQLPVGTGNKRPGDPKHSSQTSNLAHKTYS
LSSSFSSSSSTRKRGPSEETGGAVFDHPAKIAKSTKSSSLNFSFPSLPTMGQMPGHSSDTSGLSFSQPSC
KTRVPHSKLDKGPTGANGHNTTQTIDYQDTVNMLHSLLSAQGVQPTQPTAFEFVRPYSDYLNPRSGGISS
RSGNTDKPRPPPLPSEPPPPLPPLPK
>Hsa-CyclinL1
MASGPHSTATAAAAASSAAPSAGGSSSGTTTTTTTTTGGILIGDRLYSEVSLTIDHSLIPEERLSPTPSM
QDGLDLPSETDLRILGCELIQAAGILLRLPQVAMATGQVLFHRFFYSKSFVKHSFEIVAMACINLASKIE
EAPRRIRDVINVFHHLRQLRGKRTPSPLILDQNYINTKNQVIKAERRVLKELGFCVHVKHPHKIIVMYLQ
VLECERNQTLVQTAWNYMNDSLRTNVFVRFQPETIACACIYLAARALQIPLPTRPHWFLLFGTTEEEIQE
ICIETLRLYTRKKPNYELLEKEVEKRKVALQEAKLKAKGLNPDGTPALSTLGGFSPASKPSSPREVKAEE
KSPISINVKTVKKEPEDRQQASKSPYNGVRKDSKRSRNSRSASRSRSRTRSRSRSHTPRRHYNNRRSRSG
TYSSRSRSRSRSHSESPRRHHNHGSPHLKAKHTRDDLKSSNRHGHKRKKSRSRSQSKSRDHSDAAKKHRH
ERGHHRDRRERSRSFERSHKSKHHGGSRSGHGRHRR

>Hsa-CABLES1
MAAAAAAATTAACSSGSAGTDAAGASGLQQPPPQPQPQPAAAAPAQPPPEPPRKPRMDPRRRQAALSFLT
NISLDGRLPPQDAEWGGGEEGGAAKPGAGGACGARTRFSLLAAAERGGCIALAAPGTPAAGLAAGSGPCL
PQPSSLPPLIPGGHATVSGPGVARGFASPLGAGRASGEQWQPPRPAPLAACAQLQLLDGSGAAGQEELEE
DDAFISVQVPAAAFLGSGTPGSGSGSRGRLNSFTQGILPIAFSRPTSQNYCSLEQPGQGGSTSAFEQLQR
SRRRLISQRSSLETLEDIEENAPLRRCRTLSGSPRPKNFKKIHFIKNMRQHDTRNGRIVLISGRRSFCSI
FSVLPYRDSTQVGDLKLDGGRQSTGAVSLKEIIGLEGVELGADGKTVSYTQFLLPTNAFGARRNTIDSTS
SFSQFRNLSHRSLSIGRASGTQGSLDTGSDLGDFMDYDPNLLDDPQWPCGKHKRVLIFPSYMTTVIDYVK
PSDLKKDMNETFKEKFPHIKLTLSKIRSLKREMRKLAQEDCGLEEPTVAMAFVYFEKLALKGKLNKQNRK
LCAGACVLLAAKIGSDLKKHEVKHLIDKLEEKFRLNRRELIAFEFPVLVALEFALHLPEHEVMPHYRRLV
QSS
>Hsa-CyclinC
MAGNFWQSSHYLQWILDKQDLLKERQKDLKFLSEEEYWKLQIFFTNVIQALGEHLKLRQQVIATATVYFK
RFYARYSLKSIDPVLMAPTCVFLASKVEEFGVVSNTRLIAAATSVLKTRFSYAFPKEFPYRMNHILECEF
YLLELMDCCLIVYHPYRPLLQYVQDMGQEDMLLPLAWRIVNDTYRTDLCLLYPPFMIALACLHVACVVQQ
KDARQWFAELSVDMEKILEIIRVILKLYEQWKNFDERKEMATILSKMPKPKPPPNSEGEQGPNGSQNSSY
SQS
>Hsa-CyclinG1
MIEVLTTTDSQKLLHQLNALLEQESRCQPKVCGLRLIESAHDNGLRMTARLRDFEVKDLLSLTQFFGFDT
ETFSLAVNLLDRFLSKMKVQPKHLGCVGLSCFYLAVKSIEEERNVPLATDLIRISQYRFTVSDLMRMEKI
VLEKVCWKVKATTAFQFLQLYYSLLQENLPLERRNSINFERLEAQLKACHCRIIFSKAKPSVLALSIIAL
EIQAQKCVELTEGIECLQKHSKINGRDLTFWQELVSKCLTEYSSNKCSKPNVQKLKWIVSGRTARQLKHS
YYRITHLPTIPEMVP
>Hsa-CyclinH
MYHNSSQKRHWTFSSEEQLARLRADANRKFRCKAVANGKVLPNDPVFLEPHEEMTLCKYYEKRLLEFCSV
FKPAMPRSVVGTACMYFKRFYLNNSVMEYHPRIIMLTCAFLACKVDEFNVSSPQFVGNLRESPLGQEKAL
EQILEYELLLIQQLNFHLIVHNPYRPFEGFLIDLKTRYPILENPEILRKTADDFLNRIALTDAYLLYTPS
QIALTAILSSASRAGITMESYLSESLMLKENRTCLSQLLDIMKSMRNLVKKYEPPRSEEVAVLKQKLERC
HSAELALNVITKKRKGYEDDDYVSKKSKHEEEEWTDDDLVESL
>Hsa-CyclinI
MKFPGPLENQRLSFLLEKAITREAQMWKVNVRKMPSNQNVSPSQRDEVIQWLAKLKYQFNLYPETFALAS
SLLDRFLATVKAHPKYLSCIAISCFFLAAKTVEEDERIPVLKVLARDSFCGCSSSEILRMERIILDKLNW
DLHTATPLDFLHIFHAIAVSTRPQLLFSLPKLSPSQHLAVLTKQLLHCMACNQLLQFRGSMLALAMVSLE
MEKLIPDWLSLTIELLQKAQMDSSQLIHCRELVAHHLSTLQSSLPLNSVYVYRPLKHTLVTCDKGVFRLH
PSSVPGPDFSKDNSKPEVPVRGTAAFYHHLPAASGCKQTSTKRKVEEMEVDDFYDGIKRLYNEDNVSENV
GSVCGTDLSRQEGHASPCPPLQPVSVM
>Hsa-Fam58A
MEAPEGGGGGPAARGPEGQPAPEARVHFRVARFIMEAGVKLGMRSIPIATACTIYHKFFCETNLDAYDPY
LIAMSSIYLAGKVEEQHLRTRDIINVSNRYFNPSGEPLELDSRFWELRDSIVQCELLMLRVLRFQVSFQH
PHKYLLHYLVSLQNWLNRHSWQRTPVAVTAWALLRDSYHGALCLRFQAQHIAVAVLYLALQVYGVEVPAE
VEAEKPWWQVFNDDLTKPIIDNIVSDLIQIYTMDTEIP
>Hsa-CABLES2
MAAAAAGGAPGPAPGPAGPPPPAAPTSAARAPPQALRRRGDSRRRQAALFFLNNISLDGRPPSLGPGGEK
PPPPPAEAREPPAPPPPEPPTGLPARTPAPQGLLSPTQVPTGLGLDGQRQRKRVTSQRCSLEFLEDAVGC
APAQRTKHTSGSPRHKGLKKTHFIKNMRQYDTRNSRIVLICAKRSLCAAFSVLPYGEGLRISDLRVDSQK
QRHPSGGVSVSSEMVFELEGVELGADGKVVSYAKFLYPTNALVTHKSDSHGLLPTPRPSVPRTLPGSRHK
PAPTKSAPASTELGSDVGDTLEYNPNLLDDPQWPCGKHKRVLIFASYMTTVIEYVKPSDLKKDMNETFRE
KFPHVKLTLSKIRSLKREMRSLSEECSLEPVTVAMAYVYFEKLVLQGKLSKQNRKLCAGACVLLAAKISS
DLRKSGVTQLIDKLEERFRFNRRDLIGFEFTVLVALELALYLPENQVLPHYRRLTQQF
>Hsa-Fam58B
MEGMEDAGEEAGEDAGEDAREGAAAPAARVHFRVARFIMEAGVKLGMQSIPIATACTIYPKFFCETILDA
FDPYLIAMSSIYLAGKVEEQPLWAHDIISVSNRYFNPSSEPLGLDSRLWELRDSIVQRELLMLRVLRFQV
SFQHPHKYLLYYLVSLKNWLNCHSWQRTPVAVTAWALLRDSYHGGLCLRFQAQHIAVVVLYLALQVYGVE
VPAEVEAEKLWWQAFSDDLTKPIIDTIVSDLIQIYTIDTEIP
>Hsa-CyclinI2
MASGAQLPPQPSSSEVSAVQSPGGRPGAGLEETALGVPLPPSPGEAPLPRSNRSRCPGTRQPGAASLHAA
SAAVPVRPRRGTAPAGKTADAVPAAAPEQAPRPAPQSRKPRNLEGDLDERRLLCHLQLAQDREARLWRGG

KPQDEICDAFEEVVLWLLRLQNTFYFSQSTFNLALTIFGRLLISVKVKEKYLHCATITSLRLAAKVNEEE
EFIPQVKDFTKHYGSDYSPNELLRMELAILDRLHWDLYIGTPLDFLTIFHALVVLSWPHVLELLPQRNPS
LHVASLTRQLQHCMAGHQLLQFKGSTLALVIITLELERLMPGWCAPISDLLKKAQVGDMQYSCCKELVMQ
QLRSLQSSSCTDNFVSPAN
>Hsa-CyclinJL
MMDEPWWEGRVASDVHCTLREKELKLPTFRAHSPLLKSRRFFVDILTLLSSHCQLCPAARHLAVYLLDHF
MDRYNVTTSKQLYTVAVSCLLLANGVSLLSPRLKCSGMISAHCNLHLPGSSNSPASAPHPPPTPPQVAET
TGKFEDREDHVPKLEQINSTRILSSQNFTLTKKELLSTELLLLEAFSWNLCLPTPAHFLDYYLLASVSQK
DHHCHTWPTTCPRKTKECLKEYAHYFLEVTLQDHIFYKFQPSVVAAACVGASRICLQLSPYWTRDLQRIS
SYSLEHLSTCIEILLVVYDNVLKDAVAVKSQALAMVPGTPPTPTQVLFQPPAYPALGQPATTLAQFQTPV
QDLCLAYRDSLQAHRSGSLLSGSTGSSLHTPYQPLQPLDMCPVPVPASLSMHMAIAAEPRHCLATTYGSS
YFSGSHMFPTGCFDR
>Hsa-CyclinYL
MGNTLTCCVSPNASPKLGRRAGSAELYCASDIYEAVSGDAVAVAPAVVEPAELDFGEGEGHHLQHISDRE
MPEDLALESNPSDHPRASTIFLSKSQTDVREKRKSNHLNHCDLSNILPHKEQREKVPEEYFKHDPEHKFI
YRFVRTLFSAAQLTAECAIVTLVYLERLLTYAEIDICPTNWKRIVLGAILLASKVWDDQAVWNVDYCQIL
KDITVEDMNEMERHFLELLQFNINVPASVYAKYYFDLRSLADDNNLNFLFAPLSKERAQNLEAISRLCED
KDLCRAAMRRSFSADNFIGIQRSKAILS
>Hsa-CyclinY
MGNTTSCCVSSSPKLRRNAHSRLESYRPDTDLSREDTGCNLQHISDRENIDDLNMEFNPSDHPRASTIFL
SKSQTDVREKRKSLFINHHPPGQIARKYSSCSTIFLDDSTVSQPNLKYTIKCVALAIYYHIKNRDPDGRM
LLDIFDENLHPLSKSEVPPDYDKHNPEQKQIYRFVRTLFSAAQLTAECAIVTLVYLERLLTYAEIDICPA
NWKRIVLGAILLASKVWDDQAVWNVDYCQILKDITVEDMNELERQFLELLQFNINVPSSVYAKYYFDLRS
LAEANNLSFPLEPLSRERAHKLEAISRLCEDKYKDLRRSARKRSASADNLTLPRWSPAIIS
