# Supplementary material for: Roadmap to the study of gene and protein phylogeny and evolution—A practical guide
Source: PLoS One. 2023 Feb 24;18(2):e0279597. doi: 10.1371/journal.pone.0279597 (PMC9955684; doi:10.1371/journal.pone.0279597)
Supplement: S5 File — The protocol as also available on protocols.io. (PDF) [file pone.0279597.s005.pdf]

CKNKUVCW

Protocol Info: Florian G Jacques, Kristian Pietras, Paulina Bolivar, emma.hammarlund . Roadmap to the study of gene and protein phylogeny and evolution - a practical guide. **protocols.io** https://protocols.io/view/roadmap-to-the-study-of-gene-and-protein-phylogeny-cknkuvcw

Created: Dec 17, 2022

Last Modified: Dec 17, 2022

PROTOCOL integer ID: 74156

Keywords: Evolution, bioinformatics, phylogenetic analysis, evolutionary studies, molecular evolution, Phylogenetic inference

Roadmap to the study of gene and protein phylogeny and evolution - a practical guide V.(cknkuvcw)

Florian G Jacques[1], Kristian Pietras[1], Paulina Bolivar[1], emma.hammarlund[1]

[1]Lund University

Florian G Jacques

ABSTRACT

Developments in sequencing technologies and the sequencing of an ever-increasing number of genomes have revolutionisedstudies into biodiversity and organismal evolution. This accumulation of data has been paralleled by the creation of numerous public biological databases through which the scientific community can mine the sequences and annotations of genomes, transcriptomes, and proteomes of multiple species. However, to find the appropriate databases and bioinformatic tools for respective inquiries and aims can be challenging. Here, we present a compilation of DNA and protein databases, as well as bioinformatic tools for phylogenetic reconstruction and a wide range of studies on molecular evolution. We provide a protocol for information extraction from biological databases and simple phylogenetic reconstruction using probabilistic and distance methods, facilitating the study of biodiversity and evolution at the molecular level for the broad scientific community.

Introduction

1 We provide a protocol for non-bioinformatic users to reconstruct the phylogeny and evolutionary history of genes or proteins.

We present a compilation of DNA and protein databases, as well as bioinformatic tools for molecular phylogenetic reconstruction, and a wide range of studies on molecular evolution. We describe step by step the protocol from sequence harvesting from databases to phylogenetic tree building and diverse evolutionary studies, and we illustrate our protocol with two test-case studies on the evolution of P53 and Cyclins/CDKs protein families.

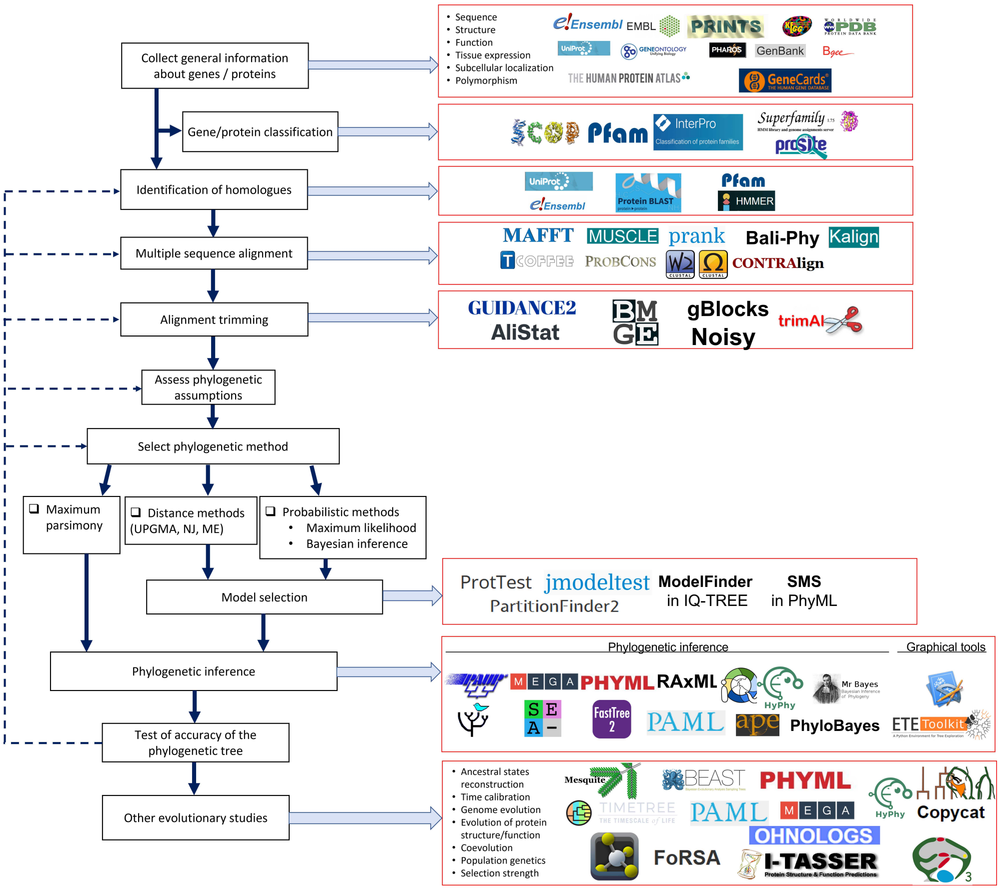

Protocol for the bioinformatic study of gene and protein phylogeny and evolution

Sequence collection and comparison

2 Collecting sequence data and bibliography on genes and proteins

Evolutionary analyses on molecular data (genes, genomes, proteins, mRNA, transposable elements, ribosomal RNA or other parts of the genome), require retrieving sequences and other information from public databases. The sequences are generally stored in the Fasta format. Other information from specific databases, including structure, activity, biological function, tissue expression, sub-cellular localiztion and polymorphism can also prove relevant for evolutionary studies.

>Search for your gene or protein of interest (e.g. Human P53) in molecular databases (e.g. ***NCBI*** *or* ***Uniprot****).*

>In Uniprot, HsTP53 is labelled as P04637 ([*https://www.uniprot.org/uniprot/P04637*](https://www.uniprot.org/uniprot/P04637)*). click on "*Sequence and Isoforms" *to display the sequence.*

*>In NCBI, select "*Nucleotide" *in the "*Database*" panel on the left, and type the name or GI number of the gene (*[*https://www.ncbi.nlm.nih.gov/nuccore/?term=Homo+sapiens+P53*](https://www.ncbi.nlm.nih.gov/nuccore/?term=Homo+sapiens+P53)*). Click on* FASTA *to display the sequence.*

>To create a Fasta file with the sequences, paste them in the Fasta format, including the headlines, in Notepad*. Save the document using* fasta *as filename extention.*

The Fasta format includes a headline starting with ">", and the nucleic acid or amino-acid sequence. For example, in the case of the human P53 protein:

>sp|P04637|P53_HUMAN Cellular tumor antigen p53 OS=Homo sapiens OX=9606 GN=TP53 PE=1 SV=4
MEEPQSDPSVEPPLSQETFSDLWKLLPENNVLSPLPSQAMDDLMLSPDDIEQWFTEDPGP
DEAPRMPEAAPPVAPAPAAPTPAAPAPAPSWPLSSSVPSQKTYQGSYGFRLGFLHSGTAK
SVTCTYSPALNKMFCQLAKTCPVQLWVDSTPPPGTRVRAMAIYKQSQHMTEVVRRCPHHE
RCSDSDGLAPPQHLIRVEGNLRVEYLDDRNTFRHSVVVPYEPPEVGSDCTTIHYNYMCNS
SCMGGMNRRPILTIITLEDSSGNLLGRNSFEVRVCACPGRDRRTEEENLRKKGEPHHELP
PGSTKRALPNNTSSSPQPKKKPLDGEYFTLQIRGRERFEMFRELNEALELKDAQAGKEPG
GSRAHSSHLKSKKGQSTSRHKKLMFKTEGPDSD

Here follow non-exhaustive lists of nucleic acid databases and protein databases, with their main features.

A	B	C
BAR	Database of plant genes and proteins	http://bar.utoronto.ca/
Bgee	Gene expression patterns	https://bgee.org/
FlyBase	Genome and proteome of the model insect *D. melanogaster*	https://flybase.org/
WormBase	Genome and proteome of the model nematode *C. elegans*	https://wormbase.org//#012-34-5
TAIR	Genome and proteome of the model plant *A. thaliana*	https://www.arabidopsis.org/
Ensembl	Genome browser of vertebrates, includes tools for identification of homolog	https://www.ensembl.org/index.html
GeneCards	Human gene function, genomics, transcription factor binding sites and protein products	https://www.genecards.org/
NCBI	Collection of databases for molecular biology and medicine providing many bioinformatics tools and services	https://www.ncbi.nlm.nih.gov/
GenBank	Annotated DNA sequences	https://www.ncbi.nlm.nih.gov/genbank/
Entrez	Gene sequences and structures	https://www.ncbi.nlm.nih.gov/Web/Search/entrezfs.html
PomBase	Genes and proteins of the model yeast *S. pombe*	https://www.pombase.org/
Xenbase	Genome and proteins of the model amphibian *X. laevis*	http://www.xenbase.org/entry/

List of nucleic acid databases

A	B	C
**Database**	**Features**	**Link**
Gene Ontology	Unified annotation of molecular function, biological processes, and cellular components of proteins	http://geneontology.org/
Human Protein Atlas	Information on human protein and their link with diseases	https://www.proteinatlas.org/
InterPro	Classification of proteins domains and functional sites	https://www.ebi.ac.uk/interpro/
KEGG	Protein function and biological pathways	https://www.genome.jp/kegg/

A	B	C
PDB	3-dimensional structures of proteins	http://www.rcsb.org/
Pfam	Information about protein families and domains, includes tools for identification of homology	http://pfam.xfam.org/
PHAROS	Centralizes literature for human proteins	https://pharos.nih.gov/
PRINTS	Protein fingerprints classification database	http://www.bioinf.man.ac.uk/dbbrowser/PRINTS/
PROSITE	Protein family, domains and functional sites	https://prosite.expasy.org/
SCOP	Structure-based classification of protein	https://scop.mrc-lmb.cam.ac.uk/
SUPERFAMILY	Protein structure and functions	https://supfam.org/
UniProt	General information on proteins, including sequence, structure, classification, function, subcellular localization and simple homology identification	https://www.uniprot.org/uniprot/

List of protein databases

2.1 **Protein domains classification** (optional)

Studying protein classification can be useful for evolutionary studies. Proteins are classified into different categories based on 3-dimensional structure, function, and evolutionary relationship. Retrieving the classification of a protein of interest and identifying the main protein domains often provides valuable insight on its diversity and evolutionary origin. Several classification systems are published and listed in the table above.

>Use a classification system (e.g. ***Pfam*** *or* ***Interpro****) to identify the main domains of a protein. Pfam presents also their occurrence in living organisms as a sunburst plot.*

According to Pfam 35.0, HsTP53 contains four main protein domains: P53 TAD (transactivating domain), TAD2, P53 DNA binding domain, and P53 tetramer. P63 and p73 also contain the P53 DNA binding domain and the P53 tetramer domain. The P53 TAD and TAD2 domains are absent in P63 and P73, but both include a single SAM_2 domain instead.

P53 (PF00870 in Pfam) is the main domain of the p53 protein, covering the amino acids 99 to 289. 1765 sequences of 382 species are present in Pfam, all in choano-organisms (metazoans and choanoflagellates), including 5 sequences in choanoflagellates and 13 sequences in the genome of *Homo sapiens* (https://pfam.xfam.org/family/PF00870#tabview=tab7).

P53 TAD and TAD2 are two transcription scaffold domains. 253 sequences containing the P53 TAD domain are present in Pfam, in bilaterians only. 81 sequences, from primates only, contain the domain TAD2. P53 tetramer serves for the oligomerization of the protein. 1392 sequences, in animals only, containing the domain p53 tetramer are present in the database. The SAM 2 (sterile alpha motif) domain is a putative protein interaction domain. More than 20000 sequences containing this domain, in more than 1400 species, are present in Pfam.

>Retrieve the classification of the protein from SCOP (https://scop.mrc-lmb.cam.ac.uk/).

The SCOP classification of p53 is as follows (accessed September 06, 2021):

- **Class b**: all beta-proteins. This class contains 178 folds.
- **Fold b.2**: common fold of b.2: Common fold of diphtheria toxin/transcription factors/cytochrome f. This fold contains 9 superfamilies.
- **Superfamily: b.2.5**: p53-like transcription factors. This superfamily contains 8 families.
- **Family: b.2.5.2**: p53 DNA-binding domain-like. 3 proteins belonging to this family are present in the database.
- **Protein p53** tumor suppressor, DNA-binding domain. The p53 proteins of 2 species are present in SCOP: *Homo sapiens* and *Mus musculus*.

>Retrieve the occurrence of the domains of the protein P53 from Pfam. Click "sequences" on the rioght to display the sunburst plot of the domain in living organisms.

The plot shows the distribution of the 1,765 sequences containing the P53 binding domain across 382 species. Every bar on the periphery represents one single species, containing one or several p53 paralogues in their genome

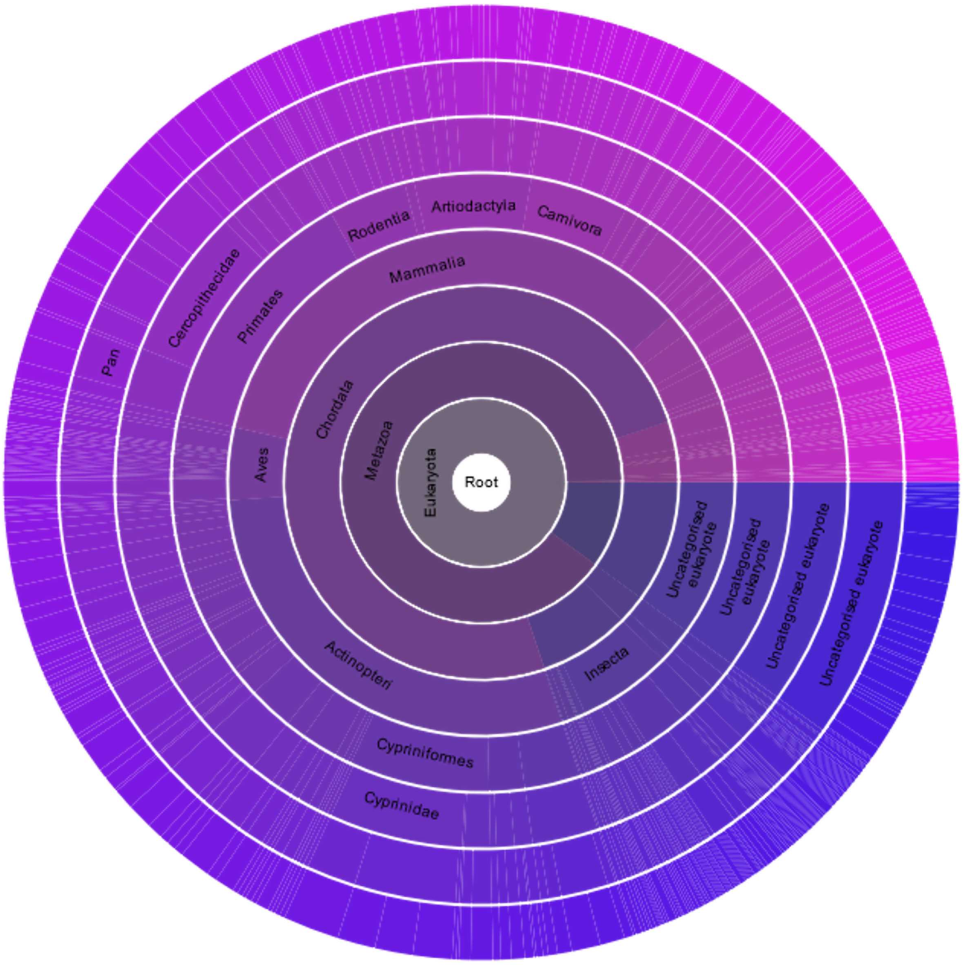

Sunburst plot of the distribution of the P53 protein domain (PF00870) in living organisms according to Pfam. This domain is present in virtually all animals, and some of their close relatives, such as choanoflagellates, and suggests that it appeared before the divergence between animals and these protists.

3 Identification of homologues

Studying the evolution of a family of genes or proteins requires the identification of homologues, *i.e.*, genes or protein with shared ancestry. Homologues include orthologues, that are present in different species and result from speciation events; and paralogues, that are present in the same genome and result from gene duplications.

Bioinformatic tools can be used to identify gene or protein homology based on sequence similarity, in the genomes of any species (see the list below). Here is a list of tools that can be used to identify sequence homology.

A	B	C
BLAST	Protein or DNA homology search from NCBI	https://blast.ncbi.nlm.nih.gov/Blast.cgi
BLAT	Sequence homology search in animal genomes	https://genome.ucsc.edu/cgi-bin/hgBlat

A	B	C
Ensembl	Genome browser of vertebrates, includes tools for identification of homology	https://m.ensembl.org/index.html
FASTA	Sequence search against protein databases and sequence alignment	https://www.ebi.ac.uk/Tools/sss/fasta/
HMMER	DNA and protein homology search	http://hmmer.org/
Pfam	Protein families and domains, includes tools for identification of homology	http://pfam.xfam.org/
SSAHA	DNA sequence search and alignment	https://www.sanger.ac.uk/tool/ssaha/
UniProt	General information on proteins, including tools for identification of similartity	https://www.uniprot.org/uniprot/

List of bioinformatic tools for the identification of gene and protein homologues

In our example, we are studying the evolution of TP53 in animals. Vertebrates have three TP53 paralogues (TP53, TP63 and TP73). Our study is based on dos Santos *et al*, Plos One, 2016.

>*Using **BLAST*** (https://blast.ncbi.nlm.nih.gov/Blast.cgi), *paste the sequence of your gene of interest (in our example, human TP53) in the Fasta format to identify homologues in the genomes of other species covering the diversity of animals (e.g. all animals and choanoflagellates), for example.*

In our example, we chose the cnidarian *Hydra vulgaris*, the fruit fly *Drosophila melanogaster, three other insects (Bombus terrestris, Apis mellifera* and *Aedes aegyptus),* the urochordate *Ciona intestinalis,* and the teleost fish *Danio rerio,* the coelacanth *Latimeria chalumnae,* the amphibian *Xenopus tropicalis,* the lizard *Anolis carolinensis,* the bird *Gallus gallus,* and the mammals *Bos taurus* and *H. sapiens.*

>*Select the homologous sequences based on E.value and significant homology (typically >30% identity). For vertebrate species, select one sequence for every paralogue (p53, p63 and p73).*

>*Download all the sequences in the Fasta format in a Fasta file.*

>*(Optional): Calculate the identity matrix of the sequences using alignment tools (e.g., CLUSTALW 2.1).*

1: M.brevicolis_A9V4M3	100.00	21.40	23.18	21.54	17.61	18.75	24.16	20.00	25.26	24.86	22.17	20.45	21.38	20.81	20.84
2: M.brevicolis_A9UZX3	21.40	100.00	24.66	24.37	19.44	21.32	28.18	23.89	29.02	28.98	26.22	23.42	23.38	24.62	23.11
3: H.sapiens_E9PFT5.1/13-155	23.18	24.66	100.00	100.00	100.00	100.00	100.00	100.00	100.00	100.00	100.00	100.00	49.68	61.79	54.67
4: H.sapiens_J3KP33.1/99-289	21.54	24.37	100.00	100.00	100.00	92.28	94.02	96.38	100.00	94.51	94.02	96.79	39.88	43.77	42.55
5: H.sapiens_E7EMR6.1/99-165	17.61	19.44	100.00	100.00	100.00	100.00	100.00	100.00	100.00	100.00	100.00	100.00	30.91	36.97	35.76
6: H.sapiens_E7EQX7.1/99-270	18.75	21.32	100.00	92.28	100.00	100.00	82.54	91.06	85.62	82.54	82.54	92.28	38.87	43.93	42.44
7: H.sapiens_A0A087WT22.1/1-130	24.16	28.18	100.00	94.02	100.00	82.54	100.00	93.05	100.00	94.51	93.05	93.05	48.11	47.59	47.59
8: H.sapiens_A0A0U1RQC9.1/60-250	20.00	23.89	100.00	96.38	100.00	91.06	93.05	100.00	100.00	95.05	89.32	92.94	39.60	41.97	40.35
9: H.sapiens_E7ESS1.1/1-157	25.26	29.02	100.00	100.00	100.00	85.62	100.00	100.00	100.00	100.00	100.00	100.00	51.78	52.76	52.76
10: H.sapiens_A0A087WXZ1.1/1-130	24.86	28.98	100.00	94.51	100.00	82.54	94.51	95.05	100.00	100.00	95.05	95.05	49.44	49.45	49.45
11: H.sapiens_A0A087X1Q1.1/1-130	22.17	26.22	100.00	94.02	100.00	82.54	93.05	89.32	100.00	95.05	100.00	100.00	44.30	44.98	45.41
12: H.sapiens_P04637.4/99-289	20.45	23.42	100.00	96.79	100.00	92.28	93.05	92.94	100.00	95.05	100.00	100.00	38.50	42.11	41.44
13: H.sapiens_Q9H3D4.1/167-359	21.38	23.38	49.68	39.88	30.91	38.87	48.11	39.60	51.78	49.44	44.30	38.50	100.00	59.66	57.19
14: H.sapiens_A0A0C4DFW9.1/68-260	20.81	24.62	61.79	43.77	36.97	43.93	47.59	41.97	52.76	49.45	44.98	42.11	59.66	100.00	84.73
15: H.sapiens_O15350.1/117-309	20.84	23.11	54.67	42.55	35.76	42.44	47.59	40.35	52.76	49.45	45.41	41.44	57.19	84.73	100.00

Percent identity matrix of the 7 p53 sequences of Homo sapiens and the 2 p53 sequences of the choanoflagellate *Monosiga brevicollis*. The matrix was realised using ClustalW 2.1.

According to the matrix, the 13 human p53 paralogues share 36% to 100% identity, and the two paralogues of *Monosiga brevicollis* share 21.4% identity. Human and *Monosiga* orthologues share 17% to 25% identity. Hence, all human paralogues are more similar to each other than to any of the *Monosiga* orthologues.

4 Multiple sequence alignment

Phylogenetic analysis requires identifying homologous bases or amino acid residues between the homologous sequences. Homology is inferred by a sequence alignment. The sequences are put in every row one after the other to arrange every homologous base or amino acid in the same column. Alignment of the homologous residues necessitates adding gaps, indicated by the symbol "-" and corresponding to insertions or deletions (indels), into the sequences.

>Use an alignment tool (e.g. MAFFT, https://www.ebi.ac.uk/Tools/msa/mafft/) to align the sequences. Paste the alignment in the Fasta format and submit. Retrieve the alignment and save it in a new Fasta file.

Here is a list of tools for nucleic acid or amino acid sequence alignment.

A	B	C
**Software**	**Features**	**Link**
BAli-Phy	Multiple sequence alignment of nucleotide and amino acid sequences and phylogenetic analysis using BI	http://www.bali-phy.org
CLUSTAL Omega*	Speed-oriented multiple sequence alignment for nucleotide or amino acid data, suitable for large datasets	https://www.ebi.ac.uk/Tools/msa/clustalo/
CLUSTALW*	Multiple sequence alignment for nucleotide or amino acid data	https://www.genome.jp/tools-bin/clustalw
CONTRAlign (ProbCons)	Accuracy-oriented multiple sequence alignment for amino acid data	http://contra.stanford.edu/contralign/
Kalign*	Multiple sequence alignment for nucleotide or amino acid data, suitable for large datasets	https://www.ebi.ac.uk/Tools/msa/kalign/
MAFFT*	Accuracy-oriented multiple sequence alignment for nucleotide or amino acid data	https://mafft.cbrc.jp/alignment/server/
MUSCLE*	Multiple sequence alignment for nucleotide or amino acid data	https://www.ebi.ac.uk/Tools/msa/muscle/
PASTA	Speed-oriented multiple sequence alignment for nucleotide or amino acid data, designed for very large datasets	https://bioinformaticshome.com/tools/msa/descriptions/PASTA.html
PRANK/ WebPRANK*	Speed-oriented multiple sequence alignment for nucleotide or amino acid data, should be preferred for close sequences and large datasets	http://wasabiapp.org/software/prank/ https://www.ebi.ac.uk/goldman-srv/webprank/
SATé	Software package for multiple sequence alignments and phylogenetic inference	https://phylo.bio.ku.edu/software/sate/sate.html
T-COFFEE*	Accuracy-oriented multiple sequence alignment of nucleotide and amino acid sequences	http://tcoffee.crg.cat/
UPP	Speed-oriented multiple sequence alignment of nucleotide and amino acid sequences, designed for very large data sets	https://github.com/smirarab/sepp.

List of programs for sequence alignment (* indicates a web interface)

5 Alignment trimming

It is recommended to check the alignment and, when necessary, to improve it manually or using alignment trimming tools. Trimming is the selection of phylogenetically informative sites in the alignment. Poorly aligned positions and highly variable regions are not phylogenetically informative, because these positions might not be homologous or subject to saturation. They should be excluded to maximize the phylogenetic signal of the alignment.

>*Use one of the tools below to compute the completeness of your alignment and exclude the poorly aligned regions (regions of the alignment with low scores).*

>*Alternatively, you can also directly download the sequences into the Guidance 2 server (http://guidance.tau.ac.il/) and proceed to the alignment using MAFFT. Open the color-coded MSA to identify poorly aligned and highly variable regions. You can delete them manually from the alignment or remove unreliable columns below a certain cutoff.*

>*The new MSA, hereafter renamed sub-MSA, will be used for the phylogenetic analysis. Save the sub-MSA in the Fasta format.*

Here is a selection of tools to quantify the completeness of alignments and selection of the phylogenetic informative regions of the alignment.

A	B	C
**Software**	**Features**	**Link**
AliStat	Quantification of alignment completeness for alignment refinement	https://github.com/thomaskf/AliStat
BMGE	Selection of informative regions on multiple sequence alignments	https://gitlab.pasteur.fr/GIPhy/BMGE
GBlocks	Selection of informative regions on multiple sequence alignments	http://molevol.cmima.csic.es/castresana/Gblocks.html
Guidance 2*	Selection of informative regions on multiple sequence alignments	http://guidance.tau.ac.il/
Noisy	Selection of informative regions on multiple sequence alignments	http://www.bioinf.uni-leipzig.de/Software/noisy/
trimAl	Selection of informative regions on multiple sequence alignments	http://trimal.cgenomics.org/

List of programs for sequence alignment trimming (* indicates a web interface)

6 Assessing phylogenetic assumptions (for more advanced users)

Phylogenetic models rely on simplifying assumptions stating for example that all sites in the alignment evolved under the same tree, that mutation rates have remained constant, and that substitutions are reversible. If the phylogenetic data violate these assumptions, the phylogeny and evolutionary analyses can be biased. Once the alignment is performed and the sites selected for phylogenetic inference, it is recommended to assess

those phylogenetic assumptions when possible. Tests for some of these assumptions have been included in IQ-TREE. You can also use the package MOTMOT, written in the R language.

Phylogenetic analysis

7 **Phylogenetic inference**

The evolutionary history of genes, proteins or species is generally presented as a phylogenetic tree, a graphical illustration of the evolutionary relationships between the sequences or taxa. Several methods for phylogenetic inference exist: Maximum Parsimony (MP), the distance-based methods and the probabilistic methods, nowadays the most widely used for molecular data.

>*Choose one or several phylogenetic methods to reconstruct the evolutionary history of your gene, protein or species of interest. See the sub-steps below for the specificities of each method.*

It can be interesting to combine several approaches and compare the results (e.g. Maximum Likelihood, Neighbor Joining and Bayesian Inference). However, confirming phylogenetic relationships with several methods does not necessarily mean that the tree is biologically correct. Several methods may give a same wrong result if they assume the same wrong assumptions and molecular evolution models.

Still, we propose to reconstruct the phylogeny of the TP53 family using a distance method: Neighbor Joining (NJ) with MEGA 11, and a probabilistic method: Maximum Likelihood (ML) using IQ-TREE 2.

Here is a list of tools for phylogenetic reconstruction using diverse methods.

A	B	C
**Software**	**Features**	**Link**
APE	R-written package for molecular phylogenetics using distance-based methods	http://ape-package.ird.fr
BAli-Phy	Phylogenetic inference using BI	http://www.bali-phy.org
BayesTraits	Phylogenetic inference and other evolutionary analyses using BI	http://www.evolution.reading.ac.uk/BayesTraitsV4.0.0/BayesTraitsV4.0.0.html
FastMe	Phylogenetic inference using distance methods	http ://www.atgc-montpellier.fr/fastme/
GARLI	Phylogenetic inference using ML	http ://evomics.org/resources/software/molecular-evolution-software/garli/
HYPHY*	Phylogenetic inference using ML and distance methods	https ://www.hyphy.org/
IQ-TREE	Phylogenetic inference using ML, including model selection and a very fast bootstrapping method	http ://www.iqtree.org/
MEGA	Sequence alignment, model selection, phylogenetic analysis using distance methods, MP and ML, and other evolutionary analyses	https://www.megasoftware.net/
MrBayes	Phylogenetic inference using BI and diverse evolutionary analyses including ancestral states reconstruction and time calibration	http://nbisweden.github.io/MrBayes/

A	B	C
PAML	phylogenetic inference using ML, estimation of selection strength, ancestral states reconstruction and other evolutionary analyses	http://abacus.gene.ucl.ac.uk/software/paml.html
PAUP	Phylogenetic inference using MP and ML	http://paup.phylosolutions.com/
PHYLIP	Phylogenetic inference using MP, distance methods and ML	https ://evolution.genetics.washington.edu/phylip.html
PhyloBayes	Phylogenetic inference with protein data using BI using a specific probabilistic model	http ://www.atgc-montpellier.fr/phylobayes/
PhyML	Phylogenetic inference using ML, ancestral states reconstruction and various evolutionary analyses	http://atgc.lirmm.fr/phyml/
PyCogent	Phylogenetic inference, tree drawing, various evolutionary analyses including partition models and ancestral states reconstruction	https ://github.com/pycogent/pycogent
RAxML	Phylogenetic inference using ML	https://cme.h-its.org/exelixis/web/software/raxml/
SeaView	Sequence alignment and phylogenetic inference using MP, NJ and ML	http://doua.prabi.fr/software/seaview
SplitsTree	Phylogenetic inference for unrooted trees and phylogenetic networks	https ://uni-tuebingen.de/fakultaeten/mathematisch-naturwissenschaftliche-fakultaet/fachbereiche/informatik/lehrstuehle/algorithms-in-bioinformatics/software/splitstree/

List of programs and packages for phylogenetic analysis using distance methods, maximum parsimony, maximum likelihood and Bayesian inference

7.1 **Selection of the molecular evolution model** (for probabilistic methods and distance methods)

Prior to phylogenetic analysis, probabilistic methods and distance methods require selection of the model of molecular evolution that best describes the data. Nucleotide or amino acid substitution models exist. They differ in the number of parameters considered, like substitution rates and base/aminoacid frequencies.

The main nucleotide substitution models are, from the simplest to the most complex: JC69, K80, F81, HKY85, TN93, GTR. The main amino acid substitution models include JTT, WAG, LG and Dayhoff. These substitution models can be associated with models of substitution rate heterogeneity between sites, such as the Gamma distribution (G) and the proportion of invariant nucleotide or amino acid sites (I). The FreeRate model (R), a more complex model of rate heterogeneity is included in ModelFinder, PhyML and IQ-TREE. The GHOST model, for alignments with variation in mutation rate, is also implemented in IQ-TREE.

The likelihood of the different models should be computed using appropriate program, such as **ModelTest** and **jModelTest** for nucleotide sequences, and **ProtTest** for amino acid sequences. **ModelFinder**, implemented in IQ-TREE, is a designed for alignments of nucleotides, codons or amino acid data. **PartitionFinder 2** can be used with nucleotide and amino acid data. Model test selectors are also included in programs such as MEGA and PhyML (**SMS**).

For the substitution model, these tools calculate the Bayesian information criterion (**BIC**) and the Akaike information criterion (**AIC**). A model with lower AIC or BIC is considered more accurate. The model optimizing BIC or AIC (*i.e.,* with the lowest score) should be selected.

>Here, we are studying protein sequences. Use ProtTest 3.4.2 to calculate the log-likelihood of a panel of 56 amino acid substitution models, and select the most relevant based on the BIC or AIC score. Select the model with the lowest score.

*>Alternatively, you can use the model selectors included in IQTREE or MEGA. For example, with the IQTREE web server (http://iqtree.cibiv.univie.ac.at/), open the "*Model Selection" *panel, download the sub-MSA, select "*protein sequences*", choose a selection criterion (AIC or BIC) and proceed to the analysis. With MEGA, download the sub-MSA, and select "*Find best DNA/protein models*" in the "*Model*" panel.*

>You will use this model to compute the phylogenetic tree of your protein and for further evolutionary analyses.

Here is a selection of tools that can be used for molecular evolution model selection.

A	B	C
**Software**	**Features**	**Link**
ModelFinder	Fast model selection with a model of rate heterogeneity between sites (nucleotide, amino acids or codons)	Implemented in IQ-TREE
ModelTest / jModelTest	Nucleotide substitution model selection	http://evomics.org/resources/software/molecular-evolution-software/modeltest/
PartitionFinder 2	Molecular evolution model selection (nucleotide or amino acids)	http://www.robertlanfear.com/partitionfinder/
ProtTest	Aminoacid substitution model selection	https://github.com/ddarriba/prottest3
SMS	Molecular evolution model selection included in PhyML (nucleotide or aminoacid)	http://www.atgc-montpellier.fr/sms/

List of programs for molecular evolution model selection

7.2 Option 1: Maximum parsimony (MP)

Maximum parsimony is a classical and simple method, that calculates the minimum number of evolutionary steps, including nucleotide insertions, deletions or substitutions, between species.

However, this method ignores hidden mutations and does not consider branch lengths, potentially leading to long branch attraction, an incorrect clustering of unrelated taxa. Furthermore, it does not consider the possibility of hidden mutations, making it not relevant for distant taxa. While MP is still used for morphological data, it is rarely used for molecular data.

PAUP, **MEGA**, **SeaView** and **Phylip** can be used for phylogenetic analysis using MP.

Phylogenetic analysis using MP with MEGA 11:
*>To reconstruct the phylogenetic tree of p53 sequences using MP, import the sub-MSA in MEGA. Then, in the "*Phylogeny *panel", choose a phylogenetic analysis using parsimony. Select the bootstrap method with at least 1000 replicates and execute the analysis.*

7.3 Option 2: Distance-based methods

Distance-based methods create a matrix of molecular distances based on the number of differences between the sequences, to reconstruct the phylogenetic tree. These methods ignore hidden mutations and are also subject to long branch attraction. Distance-based methods include the Unweighted Pair Group Method with Arithmetic mean (**UPGMA**), Neighbor Joining (**NJ**), and Minimum Evolution (**ME**).

FastME, **PAUP**, **MEGA**, **FastTree** or **Phylip** can be used for distance-based methods.

Phylogenetic analysis using NJ with MEGA 11:
>*To reconstruct the phylogenetic tree of p53 sequences using NJ, import the sub-MSA in MEGA. Then, in the "*Phylogeny" *panel, choose a phylogenetic analysis using NJ. Select the appropriate substitution model (e.g JTT+G) and the bootstrap method with at least 1000 replicates. Execute the analysis.*

7.4 Option 3: Probabilistic methods (requires selection of the molecular evolution model, see below)

The strength of probabilistic methods is the use of specified models of molecular evolution. Probabilistic methods consider different mutation rates between sites to avoid mutation saturation. Nowadays, most studies of phylogenetic reconstruction use probabilistic methods.

They include Maximum Likelihood (**ML**, described below in the section Option 3-1) and Bayesian Inference (**BI**, described below in the section Option 3-2). ML calculates the probability of observing the data (in this case, the sequence alignment) under different explicit models of molecular evolution. ML aims to identify the best fit model by exploring multiple combinations of model parameters. Inversely, BI evaluates the probability of each substitution model given the data.

7.5 Option 3-1: Maximum Likelihood (ML)

Many programs for ML-based phylogenetic analysis exist. For beginners, we recommend **SeaView** or **MEGA**, which include several tools for sequence alignment, phylogenetic inference including probabilistic methods, and a tree editor. **IQ-TREE**, that includes ModelFinder and a very fast bootstrapping method (UFBOOT2), is reported to be both fast and accurate. IQ-TREE also includes a web version. **PhyML** is accurate, easy of use and, like **PAUP** and **MEGA**, includes many common models of molecular evolution. PhyML also includes a web interface. **RAxML** and particularly **FastTree** are fast and well suited for large datasets (up to 1 million sequences with FastTree). They use only a specific model of rate heterogeneity (CAT), in addition to the Gamma law and the proportion of invariant sites. Like **Garli**, their choice of nucleotide evolution model is limited to GTR. **PAUP** is slower than other programs, and uses nucleotide data only.

>*Choose a program relevant with the type and size of your dataset* (see Table in Step 6).

For ML-based phylogenetic analysis with IQ-TREE 2:
>*Download the sub-MSA, select the appropriate sequence type (DNA or protein) and the appropriate substitution model (e.g JTT+G). In the panel "*branch support analysis*", select the Ultrafast Bootstrap analysis with at least 1000 replicates. For single branch tests, you can also select the SH-aLRT test. Execute the analysis.*

7.6 Option 3-2: Bayesian Inference (BI)

The most recent method for phylogenetic reconstruction uses Bayesian Inference (BI), which calculates the probability of the molecular evolution model given the data. The main software used for BI-based phylogenetics are **MrBayes** and **BEAST**, that use the Markov Chain Monte Carlo (MCMC) algorithm. **PhyloBayes** is a Bayesian MCMC sampler for phylogenetic reconstruction with protein data using a

specific probabilistic model, well adapted for large datasets and phylogenomics. **Bali-Phy** can also be used for phylogenetic analysis using BI.

8 Tree rooting

The root of a phylogenetic tree is the hypothetical last common ancestor of all the taxa present in the tree. Phylogenetic trees can be unrooted or rooted. The latter corresponds to the identification of ancestral and derived states, aiming at studying the direction of the evolution of the sequences.

Diverse rooting methods exist. The most common requires outgroups (taxa that do not belong to the studied group but are closely related) in the analysis. Typically, two outgroups are selected, one being more closely related to the ingroup than the other, allowing for a proper identification of the states of characters. Alternative methods include the Midpoint rooting, which places the root at the mid-point of the longest branches, and the molecular clock rooting, which assumes that the evolution speed is constant between the sequences.

>In our example, we include the P53 homologues from the choanoflagellate *Monosiga brevicollis* and the Cnidarian *Hydra vulgaris. Designate the P53 of* M. brevicollis *as the outgroup when drawing the phylogenetic tree with graphical programs.*

*>Alternatively, select "*Midpoint rooting*" when drawing the phylogenetic tree with graphical programs.*

9 Tree drawing

Once the phylogenetic tree has been computed, it can be exported using *e.g.* Newick file format and visualized using a graphical software such as **FigTree**, **ETE Toolkit** or **ITOL**. **MEGA** and **SeaView** also include visualization tools. Using different sets of options, several types of phylogenetic trees can be drawn (rooted or not, cladogram or phylogram), and branch support values (bootstrap values or posterior probabilities) can be displayed.

With Mega 11:
>Click file > export current tree (Newick), select Bootstrap and branch length, retrieve the phylogenetic tree in the Newick format and save it with nwk as filename extention.

With IQ-TREE 2:
>Paste the phylogenetic tree in the Newick format to Notepad, and save it using nwk as filename extension.

>Use a program (e.g. FigTree) in the list below and open the nwk files. You can also directly paste the phylogeny in the Newick format in the graphical tools.

>Many options of tree drawing are available. For example, you can display the bootstrap values, the posterior probabilities, or the SH-aLRT values, collapse clades below a certain bootstrap threshold (e.g. 50), and highlight or add color to the clades.

A	B	C
**Software**	**Features**	**Link**
ETE Toolkit	Visualization and analysis of phylogenetic trees	http://etetoolkit.org/
FigTree	Graphic software for phylogenetic trees	http://tree.bio.ed.ac.uk/software/figtree/

A	B	C
ITOL*	Visualization and annotation of phylogenetic trees	https://itol.embl.de/
MEGA	Sequence alignment, model selection, phylogenetic analysis, includes tree visualization and annotation tools	https://www.megasoftware.net/
SeaView	Sequence alignment and phylogenetic inference, includes tree visualization and annotation tools	http://doua.prabi.fr/software/seaview

List of tools for graphical visualization and annotation of phylogenetic trees

In our example, we used **ITOL** for the graphical representation of the phylogenetic tree of the P53 family. Both methods reveal four major clades containing respectively the p53 of insects and the p53, p63, and p73 of all vertebrates. The p53, p63, and p73 of vertebrates are more closely related to each other than to any other p53. Furthermore, the p63 and p73 of vertebrates are more closely related to each other than to vertebrate p53. This indicates that two duplication events in the p53 family preceded the origin of vertebrates. First, the p53 family and the p63/p73 cluster diverged. The second one caused the p63 and p73 families to diverge. The p53 of insects are clustered together. This indicates that insects diverged from the other bilaterians before these two duplications.

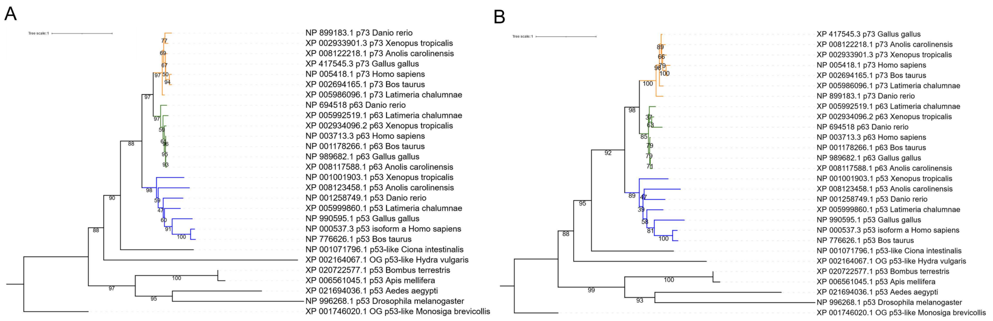

Phylogenetic trees of p53 domain-containing proteins of metazoans using Neighbour Joining (A) and Maximum Likelihood (B). The trees were realized according to the model JTT+G, as calculated by ModelFinder using AIC. The numbers indicate the bootstrap values as calculated by the standard bootstrapping method and UFBoot2, respectively. The phylogenetic trees were inferred using MEGA 11 and IQ-TREE 2, respectively, and the figures were generated using ITOL. Green branches represent the p63 family, orange branches represent the p73 family and blue branches represent the p53 family.

Evolutionary analyses using phylogenetic trees

10 Reconstruct the evolution of the gene or protein

Sequence alignments and phylogenetic trees can be used to reconstruct diverse aspects of the evolutionary history of genes, proteins and species, as well as the study the genetic structures within populations. In this last section, we provide a brief and non-exhaustive overview of evolutionary studies that can be performed using bioinformatic tools.

10.1 Phylogenetic calibration

Phylogenetic calibration consists in estimating the age of speciation or duplication events (the nodes in the phylogenetic tree), using events with a known age, for example fossil data and other geological data (that can only give minimal ages) as calibration points. Alternatively, mutation rates can be used to calculate the divergence time between two sequences.

Databases such as **TimeTree**, compute the estimated divergence time between species. **Mesquite** also provides tools to calibrate phylogenetic trees in geological times using fossil data. **Ohnologs** can be used to estimate the divergence time between homologues resulting from whole genome duplications in vertebrates.

To calibrate the phylogenetic tree of P53 with Mega 11 and TimeTree:
>*Download the alignment file in the Fasta format and the tree file in the Newick format in MEGA. In the* Compute *panel, select "*Compute Timetree*".*

>*In the* Specify outgroup *section, define* Monosiga brevicollis *as the outgroup by moving it to the* Outgroups *panel. In the section* Calibrate nodes, *select "*Internal nodes constraints*".*

>*In TimeTree (http://www.timetree.org/), enter the names of species to retrieve their estimated divergence time. For example,* Homo *and* Drosophila *diverged between 630 and 830 million years ago, with 694 million years as median time.*

>*In MEGA 11, click "*add new calibration point*" and select the node in the phylogenetic tree, or enter the names of the two taxa, and define the speciation age with a minimum, maximum or fixed time (for example, 694 million years between* Homo *and* Drosophila*). Use TimeTree to define several calibration points between different species in the tree before and after the duplication events.*

>*Click "*Launch the analysis"*, and retrieve and save the calibrated tree. You can export it with the divergence times. Use the graphical programs to add color, highlight clades, etc...*

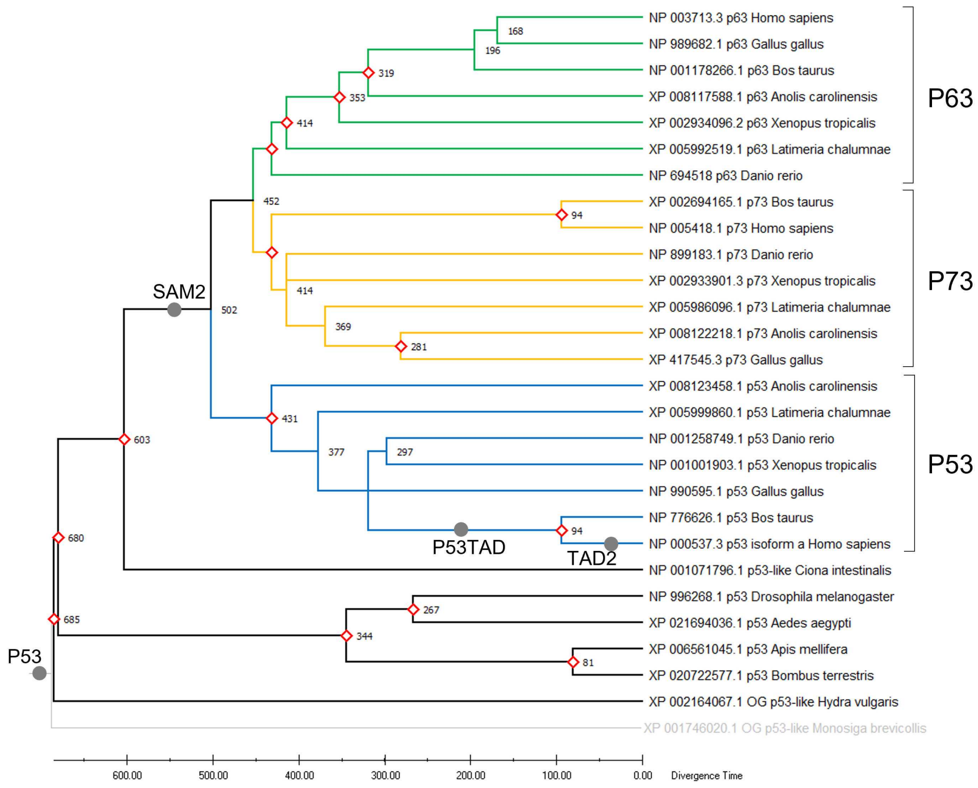

Time-calibrated phylogenetic tree of p53 domain-containing proteins of metazoans. The tree was realized according to the model JTT+G as calculated by ModelFinder using AIC. The phylogenetic tree and the figure were realized using MEGA 11. Time calibration was performed using TimeTree. The values on the branches and the scale indicate the divergence time in million years. Green branches represent the p63 family, orange branches represent the p73 family and blue branches represent the p53 family. Grey spots on the branches indicate the origin of the different protein domains during the evolution of the TP53 family.

A	B	C
**Software**	**Features**	**Link**
BayesTraits	Evolutionary analyses using Bayesian inference	http://www.evolution.rdg.ac.uk/BayesTraitsV3.0.5/BayesTraitsV3.0.5.html
BEAST	Diverse evolutionary analyses using Bayesian inference, including phylogenetic analysis, calibration and molecular clock	http://www.beast.community
Mega	Sequence alignment, model selection, phylogenetic analysis (parsimony, distance methods)	https://www.megasoftware.net/
Mesquite	Comparative analyses and statistics	http://www.mesquiteproject.org/
MrBayes	Bayesian phylogenetic inference, ancestral states reconstruction, phylogenetic calibration…	http://nbisweden.github.io/MrBayes/
Ohnologs	Database of vertebrate ohnologues, resulting from whole genome duplications	http://ohnologs.curie.fr/
Timetree	Tree calibration	http://www.timetree.org/

List of programs and databases that can be used for time-calibration of phylogenetic trees

using diverse methods

10.2 Reconstruction of ancestral states

Retracing the functional evolution of genes, proteins, or biological traits often requires the reconstitution of ancestral states. Ancestral states can be inferred from a phylogenetic tree using MP, ML, or BI; and requires the aligned sequences and the model of molecular evolution that has been used for the phylogenetic analysis when using probabilistic and distance methods.

Reconstruction of ancestral states with Mega 11:
>Import the alignment file in the Fasta format, and the tree file in the Newick format in MEGA. In the Ancestors *panel click "*Infer ancestral sequences*" and select the method (MP or ML). In ML, select the appropriate substitution model. Launch the analysis. The software displays the reconstructed ancestral state at every site of the sequence at every node.*

A	B	C
**Software**	**Features**	**Link**
BayesTraits	Evolutionary analyses using Bayesian inference	http://www.evolution.rdg.ac.uk/BayesTraitsV3.0.5.html
BEAST	Diverse evolutionary analyses using BI, including phylogenetic analysis, calibration and molecular clock	http://www.beast.community
Mega	Sequence alignment, model selection, phylogenetic analysis (parsimony, distance methods)	https://www.megasoftware.net/
Mesquite	Comparative analyses and statistics	http://www.mesquiteproject.org/
MrBayes	Bayesian phylogenetic inference, ancestral states reconstruction, phylogenetic calibration…	http://nbisweden.github.io/MrBayes/
PAML	Maximum likelihood phylogenetic inference, estimation of selection strength, ancestral states reconstruction and other analyses	http://abacus.gene.ucl.ac.uk/software/paml.html
RASP	Ancestral states reconstruction	http://mnh.scu.edu.cn/soft/blog/RASP/index.html

List of programs for ancestral states reconstruction

10.3 Measure of selection strength

The type and strength of selection on protein coding genes may be of interest. It is calculated by evaluating the ratio of the number of non-synonymous substitutions (substitutions changing the protein sequence) per non-synonymous site (dN), and the number of synonymous substitutions (substitutions with no effect on the protein sequence due to the redundancy of the genetic code) per synonymous site (dS). If dN/dS > 1, then the non-synonymous substitutions are higher than expected and the gene is under positive selection. If dN/dS<1, the gene is under purifying selection and if dN/dS=1, the selection is neutral. It is recommended not to use the dN/dS ratio for closely related species. The ratio can be calculated using **PAML**, **MEGA**, **Bio++** and **HyPhy**.

Measure of selection strength with Mega 11:

>Import the alignment file in the Fasta format, and the tree file in the Newick format in MEGA. In the Selection *panel click "*Infer ancestral sequences*".*

10.4 Study of co-evolution

Co-evolution refers to the genetic and/or morphological changes between different species in interaction. It is widely used in evolutionary ecology and parasitology to study the evolution of hosts and parasites. Co-evolutionary events include co-speciation, host change, duplication and loss of interaction. The evolution of the parasite is partly driven by the evolution of the host, which is considered independent from the evolution of the parasite. The co-evolutionary history can be presented as a co-phylogeny with the two entities.

Some programs for studying co-evolution, including **Jane**, **CoRe-PA** and **TreeMap** [170] (**Table 8**), are based on the hypothesis that the evolution of the parasite is driven by the evolution of the host. Others, such as **Copycat** [162], reconciliate the two phylogenies under the hypothesis that the situation is symmetric and evaluate the significance of co-evolution under a statistical framework. Co-evolution of genes or proteins can also be studied using these tools. However, one should keep in mind that in this context, the co-evolution between the two entities is symmetric.

In our second test-case study, we used ML to reconstruct the evolution of cyclins and CDKs, two families of proteins involved in cell cycle control and closely interacting. We used **Jane** and **TreeMap** to reconstruct (cophylogenies between the two families.

>With Jane and TreeMap, a single nexus file containing the phylogenies of cyclins and CDKs, and their associations is needed. Create a nexus file (starting with #NEXUS). This file should contain the two trees in Newick format, in the sections BEGIN HOST and BEGIN PARASITE, and the associations in the section BEGIN DISTRIBUTION. This section should mention every association between Cyclins and CDK following the pattern "Host: Parasite,". All three sections should end with "ENDBLOCK;". The names of the taxa in the three files should be identical. Cyclins interacting with several CDKs and vice versa should be repeated.

>Import this file to Jane and launch the analysis in the Solve Mode. The costs of coevolutionary events can be set. The stats mode can be used to compute the cost range of the solutions. With TreeMap, import the nexus file and launch the analysis in "Solve the tanglegram". We optain a coevolutionary scenario that represents the best way to associate the two trees. You can test the significance of the reconstruction in "estimate significance" or perform a heuristic test.

In both figures, the clustering of cyclins and CDKs indicate an interaction (the cyclin can bind the CDK and activate it). Red spots indicate significant events of coevolution between the two families of proteins. Co-speciation (hollow red circle), duplications (solid red circle), duplications with host switch (yellow circle), loss of interaction (dashed lines), failures to diverge (jagged lines) are indicated on the figure.

A	B	C
**Software**	**Features**	**Link**
Copycat	Co-evolution studies	http://www.cophylogenetics.com/
Core-PA	Co-evolution studies	http://pacosy.informatik.uni-leipzig.de/49-1-CoRe-PA.html
Jane	Co-evolution studies	https://www.cs.hmc.edu/~hadas/jane/
TreeMap	Co-evolution studies	https://sites.google.com/site/cophylogeny/treemap

List of programs to study coevolution

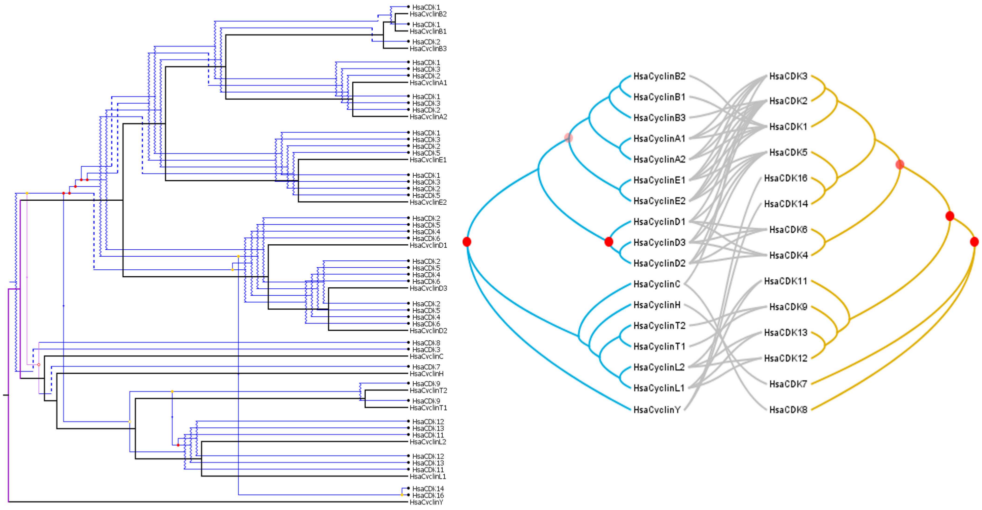

Two co-evolutionary scenarios of the associations between, and co-evolution of human cyclins and CDKs

10.5 Genome evolution

Evolutionary events, such as mutations, insertions, deletions, gene or whole genome duplications, genome reorganization, and genetic exchanges can be identified using phylogenetic trees in complement with genomics tools and databases. Here is a list of databases tools to study diverse aspects of genome evolution, including genome browsers of diverse lineages and tools for comparative genomics and evolutionary genomics:

A	B	C
**Software**	**Features**	**Link**
BAR	Database of plant genes and proteins	http://bar.utoronto.ca/
CAFE	Gene family evolution	https://github.com/hahnlab/CAFE5
CoGE	Comparative genomics analyses	https://genomevolution.org/coge/
Ensembl	Genome browser of vertebrates, includes tools for identification of homology	https://www.ensembl.org/index.html
Entrez	Gene sequences and structures	https://www.ncbi.nlm.nih.gov/Web/Search/entrezfs.html
FlyBase	Genome and proteins of the model insect *D. melanogaster*	https://flybase.org/
GenBank	Annotated DNA sequences	https://www.ncbi.nlm.nih.gov/genbank/
HGT-Finder	Horizontal gene transfer finding	http://cys.bios.niu.edu/HGTFinder/HGTFinder.tar.gz
Ohnologs	Database of vertebrate ohnologues, resulting from whole genome duplications	http://ohnologs.curie.fr/

A	B	C
PomBase	Genes and proteins of the model yeast *S. pombe*	https://www.pombase.org/
TAIR	Genome and proteins of the model plant *A. thaliana*	https://www.arabidopsis.org/
WormBase	Genome and proteins of the model nematode *C. elegans*	https://wormbase.org//#012-34-5
Xenbase	Genome and proteins of the model amphibian *X. laevis*	http://www.xenbase.org/entry/

List of programs and databases to study genome evolution

10.6 Phylogenetic comparative analysis

Evolutionary biology often employs the so-called phylogenetic comparative methods to study the adaptive significance of biological traits. These methods aim at identifying biological characters, in terms of morphology, physiology, or ecology, that result from a shared ancestry. Comparative analyses can be done for quantitative or qualitative variables. **Mesquite** is a very appropriate tool for comparative analysis and to compute statistics on phylogenetic trees. **BayesTraits** can also be used.

10.7 Population genetics

Genetic diversity can also be explored at the population level by analyzing polymorphism between members of the same species. Bioinformatic tools are designed to study allele diversity within a population, including single nucleotide polymorphisms (SNPs), indels, microsatellites or transposable elements. Mathematical models have been developed to describe polymorphism. Several programs are suitable for population genetics studies.

A	B	C
Software	Features	Link
DNAsp	Analysis of DNA polymorphism	http://www.ub.edu/dnasp/
Genepop	Population genetics analyses	https://genepop.curtin.edu.au/
SNiplay	SNP detection and other population genetics analyses	https://sniplay.southgreen.fr/cgi-bin/home.cgi
Arlequin	Population genetics analyses	http://cmpg.unibe.ch/software/arlequin35/

List of programs and databases for population genetics

10.8 Study of protein structure and function evolution

Studying the functional evolution of proteins can require structure alignments, that can be realized by appropriate programs such as **PyMol**, and the mean distance in ångström between homologous residues can be calculated.

Protein structures are described by databases such as the Protein Data Bank (**PDB**). The PDB provides the

3-dimensional structures of proteins and their interacting ligands established by X-ray crystallography, electron microscopy, or NMR spectroscopy, which can be retrieved as pdb files. The PDB also displays a 3D visualization tool, programs for 3D analyses such as pairwise structure alignment and pairwise symmetry, and cross links to other protein databases. Annotation for protein families based on fingerprints, *i.e.*, conserved 3-dimensional motifs specific for a protein family, are gathered in the database **PRINTS**. PRINTS includes a 3D visualization software and search tools for protein sequence homology and pairwise or multiple sequence alignments.

I-TASSER, **HHPred** of the HH suite and **Alpha fold** can be used to predict the 3-dimensional structure of proteins from their amino-acid sequences. **FoRSA** is able to identify a protein fold from its amino acid sequence or a protein sequence in the proteome of a species from a crystal structure.

Here is a list of tools that can be used for analyses on protein structures in an evolutionary framework:

A	B	C
**Software**	**Features**	**Link**
PyMol	3D visualization of molecules	http://www.mesquiteproject.org/
PDB	3-dimensional structures of proteins	https://www.rcsb.org/
Prints	Protein fingerprints classification	http://www.bioinf.man.ac.uk/dbbrowser/PRINTS/
PyMol	3D visualization of molecules and diverse analyses on protein structures	https://pymol.org/2/
I-Tasser	Protein structure prediction	https://zhanglab.dcmb.med.umich.edu/I-TASSER/
Forsa	Protein structure prediction	http://www.bo-protscience.fr/forsa/
HHPred	Protein structure prediction	https://toolkit.tuebingen.mpg.de/tools/hhpred

List of programs for protein structure analyses
