# Supplementary material for: Roadmap to the study of gene and protein phylogeny and evolution—A practical guide
Source: PLoS One. 2023 Feb 24;18(2):e0279597. doi: 10.1371/journal.pone.0279597 (PMC9955684; doi:10.1371/journal.pone.0279597)
Supplement: Attachment — Submitted filename: Rebuttal_letter_PlosOne_Jacques et al.pdf [file pone.0279597.s006.pdf]

FACULTY OF MEDICINE
Lund University
Department of Laboratory Medicine
Translational Cancer Research

Lund, 07/07/2022

Editor
PlosOne

Dear editor,

We thank the reviewers for their helpful and insightful comments on our manuscript entitled "*Roadmap to the study of gene and protein evolution*" by Florian Jacques, Paulina Bolivar, Kristian Pietras, and Emma Hammarlund. We have revised the manuscript in accordance with most of their comments and concerns. We believe that their thorough review and our revision has resulted in a much-improved contribution. Below, we reply to all the comments one by one. Our replies are marked in green for when agree and red for when we disagree to some degree. We refer to new intervals of text by their row number in the manuscript.

Reviewer 1:

1. In the sequence alignment session, the authors already mentioned that ClustalW is suitable for large dataset, while MAFFT has the highest accuracy. Why hasn't MAFFT been used in the two examples instead of Clustal2.1, even though the dataset was small enough for MAFFT? How will it affect the result by using different alignment tool?

Yes, we agree and thank the reviewer for pointing it out. We used MAFFT in our first example and ClustalW in the second example, because they are two of the most user-friendly for beginners since they have a web interface. In our first example, ClustalW has been used only to generate the percent identity matrix. In the introduction we have now also clarified the overall aim to highlight the databases and tools that display a web-user interface. In our examples, the differences in results (topology tree and nodes robustness) with MAFFT or ClustalW is minor. Although a comparison of the different results could be interesting, we refrain from discussing it here since it is beyond the scope of this guide to the practical tools. More specifically, edits are made at

Row 69-70: 'The aim is to provide a practical guide for beginners and more advanced explorers into protein and gene evolution.'

Row 233-235: ' **PROBCONS** [42], **T-COFFEE** [41] and **MAFFT** [40] are described to have particularly high accuracy but also high calculation times [45]. They are suitable for small and intermediate datasets.'

2. Both two examples considered small datasets. For example, the reconstruction of the evolutionary history of p53 family has been done with only 11 taxa. It is, therefore, doable by

either manually typing the species to the search organism field or downloading protein sequences for each species from the online BLAST search result. However, in many cases, especially while studying about the evolutionary history of a gene, one needs a large and more diverse taxa set (i.e. https://doi.org/10.3389/fmicb.2021.739000). It would be appropriate to have an instruction for effectively batch downloading the required data. Or, in general, how to deal with retrieving large data?

Yes, this is a valid comment. We have now revised the text to clarify how to extract a large number of sequences from NCBI using different identifiers, and how to save them into a fasta file. Also, we now introduce the Batch Entrez tool. More specifically, edits are made at

Row 121-123: 'It is also possible to batch download a large number of sequences from NCBI, by entering their identifiers (accession numbers, gi numbers or GeneIDs) in Batch Entrez.'

Row 133-134: 'The "retrieve/ID mapping" tool of UniProt allows to batch download the information on a list of proteins using UniProt identifiers.'

3. The databases are intensively represented. The information, such as the gene or protein sequences, can be redundant between databases. Nevertheless, their identifiers are often different. It would be helpful to quickly outline the approaches to communicate between different resources, for example by using the ID mapping tool of UniProt (https://www.uniprot.org/uploadlists/).

Yes, we thank the reviewer also for pointing this out. We have revised the text to clarify how to communicate between the identifiers of several databases and introduced the ID mapping tool of Uniprot. More specifically, edits are made at

Row 130-132: 'The "retrieve/ID mapping" tool of UniProt allows to batch download the information on a list of proteins using UniProt identifiers.'

4. Similar to the issue with the databases, a large number of tools has been introduced. However, many of them are just superficially described, especially the tools for the phylogenetic analysis in table 6 and 7. Thus, this can puzzle the users for choosing the right approach for their study, which consequently defeats the purpose of this manuscript.

We understand the reviewers concern here. There is a delicate balance when condensing a complex field into a practical guide that is aimed also for beginners (as well as somewhat more advanced users). We have tried to go into some more depth while clarifying how to choose the paths forward. More specifically, we have added several tools and bioinformatic methods for sequence alignment and trimming, model selection and phylogenetic inference, as well as comments on their specificities, including models of molecular evolution and bootstrapping methods. We edited the protocol, so it is more comprehensive, more clear and more user-friendly for non-bioinformatic users (and also stated this in the introduction). We detailed more the specificities of databases and tools in Tables 1, 2, 3, 4, 5, 6, 7, 8, 9, 10, and their corresponding paragraphs to facilitate the choice of appropriate method for the protocol users.

We explained more in detail the different methods for sequence alignments, and added several programs that can be used, with their specificities and comments on how to choose between them, mostly based on the size of the dataset. Specifically, edits are made on:

FACULTY OF MEDICINE
Lund University

Department of Laboratory Medicine
Translational Cancer Research

Row 232-243: '**CLUSTALW** [34] and **MUSCLE** [36] are included in **MEGA** [44]. They display web interfaces, as well as **MAFFT** [40], **Kalign** [39], and **PRANKS** [37,55]. **PROBCONS** [42], **T-COFFEE** [41] and **MAFFT** [40] are described to have particularly high accuracy but also high calculation times [45]. They should be restricted to small and intermediate datasets. **CLUSTAL Omega** [35] and **Kalign** [39] are particularly fast, but less accurate [46]. They can be used for datasets of up to 4000 and 2000 sequences, respectively [45,46]. The performances of **MUSCLE** are intermediate [46]. **PRANK** is particularly accurate for large sets and closely related sequences. **Bali-Phy** [47] performs a bayesian co-estimation of alignment, phylogeny, and other parameters and is also argued to be very reliable. **PASTA** [48] and **UPP** [49], that uses a machine learning technique, are designed for very large datasets. **MAFFT** offers a wide range of methods, which can be accuracy-oriented, such as L-INS-i, G-INS-i and E-INS-i; or speed-oriented, such as FFT-NS-2, which can be used for up to 30 000 sequences.'

We also added information on the different phylogenetic methods. They are now in separate paragraphs. We added several programs that can be used for phylogenetic inference, with some of their specificities and comments on how to choose between them, mostly based on the size of the datasets, but also their diverse options (type of data, models implemented, branch support test). Specifically, edits are made on:

Row 332-341 : '**MEGA** [44] and **SeaView** [104] are known to be very user-friendly. They include sequence alignment tools and tree editors. **PhyML** [105] is accurate, easy of use and, like **PAUP** [103] and **MEGA** [44], includes all common models of molecular evolution. **RAxML** [106] and particularly **FastTree** [100] are fast and well suited for large datasets (up to 1 million sequences with FastTree). They use a specific model of rate heterogeneity, in addition to Gamma law and proportion of invariant sites. **IQ-TREE** [60,61], that includes ModelFinder [84] and a very fast bootstrapping method, is reported to be both fast and accurate [111]. **PAUP** is slower than other programs and uses nucleotide data only.'

Row 345-349: 'The main software used for BI-based phylogenetics is **MrBayes** [112] that uses the Markov Chain Monte Carlo (MCMC) algorithm (**Table 7**). **PhyloBayes** [113,114] is a bayesian MCMC sampler for phylogenetic reconstruction with protein data using a specific probabilistic model, well adapted for large datasets and phylogenomics. **Bali-Phy** [47] can also be used for phylogenetic analysis using Bayesian inference.'

To make the protocol more comprehensible and easier of use for beginner, we also added information on bioinformatic tools and how to use them, in the text and in the tables. More specifically, we added precisions on the tools used by the workflows.

Row 399-406: 'The Cyberinfrastructure for phylogenetic research (**CIPRES science gateway**) [129] is a public resource for phylogenetic analysis that includes most tools and software for sequence alignment, model selection, and phylogenetic inference, including BEAST, FastTree, GARLI, IQ-TREE, jModelTest, MAFFT, MrBayes, PAUP, PhyloBayes and RAxML. Other packages include **NGPhylogeny** [130], a web service for phylogenetic analysis from sequence

Lund University Cancer Center, Division of Translational Cancer Research, Department of Laboratory Medicine, Lund, Sweden. Mail: florian.jacques@med.lu.se

alignment to tree inference, and **Phylemon** [131], a suite of web tools for phylogenetics, phylogenomics, molecular evolution studies and hypothesis testing.'

Table 8: List of packages for phylogenetic analysis and evolutionary studies (*: tools that include web interface)

**Software**	**Features**	**Link**	**References**
Bio++	Software package for sequence analyses, phylogenetic analysis, molecular evolution studies and population genetics analyses	https://github.com/BioPP	[128,132]
CIPRES	Server providing a software package for diverse phylogenetic analyses, including BEAST, FastTree, GARLI, IQ-TREE, jModelTest, MAFFT, MrBayes, PAUP, PhyloBayes and RAxML	https://www.phylo.org/	[129]
HyPhy	Software package for evolutionary analyses including evolution model selection, phylogenetic inference using ML and distance methods and sequence evolution studies	https://www.hyphy.org/	[108]
NGPhylogeny *	Workflows that integrate numerous methods and tools for multiple sequence alignment (MAFFT), trimming (BMGE), tree inference (FastTree, FastME or PhyML) and newick display	https://ngphylogeny.fr	[130]
Phylemon2*	Web-tools for molecular evolution, phylogenetics, phylogenomics, molecular evolution studies and hypotheses testing.	http://phylemon.bioinfo.cipf.es/index.html	[131]

Row 507-513: 'Structure alignments can be realized to compare protein functions and evolution, and the mean distance in Å between homologous residues can be calculated. **I-TASSER** [159] and **HHPred** of the HH-suite software [160] can predict 3-dimentional structure for protein sequences using homology information. **FoRSA** [161] uses a structural alphabet known as Protein Blocks to identify a protein fold from its amino acid sequence, or to identify a protein sequence in the proteome of a species from a crystal structure by calculating a likelihood score.'

Table 10: List of programs for protein structure analyses

FACULTY OF MEDICINE
Lund University
Department of Laboratory Medicine
Translational Cancer Research

Software	Features	Link	References
FoRSA	Protein structure prediction using a structural alphabet	http://www.bo-protscience.fr/forsa/	[161]
HHPred	Protein structure prediction using homology information	https://toolkit.tuebingen.mpg.de/tools/hhpred	[160]
I-TASSER	Protein structure prediction and structure-based function annotation	https://zhanglab.dcmb.med.umich.edu/I-TASSER/	[159]
PyMOL	3D visualization of molecules and diverse analyses on protein structures	https://pymol.org/2/	[158]

FigTree should not belong to the list of nucleic acid databases (table 1).

We removed FigTree from Table 1

1. Link for UniProt in table 2 should not be directed to a specific protein (P04637) but be generalized.

We changed the link to the generalized web page.

3. Gblocks Server is no longer available (link in table 4).

The broken link was replaced with the valid one.

4. Links for BayesTraits, HGT-Finder in table 6 and Bio++ in table 7 do not work (tested on May 15th).

The links were replaced with valid ones.

5. Link for PyMol in table 8 is directed to Mesquite.

Lund University Cancer Center, Division of Translational Cancer Research, Department of Laboratory Medicine, Lund, Sweden. Mail: florian.jacques@med.lu.se

The link was replaced with a link to PyMol

6. The reference for some tools are missing in the main text and tables. Namely, ClustalOmega, Probcons (table 4), BayesTraits, FigTree, PAUL (table 6), and Bio++ Suite (table 7).

References were added to each of these tools.

Reviewer 2:

In general, I think there is merit in publishing a manuscript like the one submitted. However, I also think you could make it more comprehensive and more accurate, especially regarding the section on the protocol and phylogenetic analysis. Regarding the protocol, I think readers would need to have a more detail decision tree that offers them alternative paths, depending on what their objectives and data are. You need to incorporate some measures of quality control at different steps in the protocol, and you need feedback loops that are followed in case the result of a quality control is that the preceding analysis did not yield the expected result (otherwise you will perpetuate errors made at the early steps). In this regard, your protocol is a little like that in Ciccarelli et al. (2006; Science 311, 1283-1287).

We are grateful that the reviewer also sees the merit in this practical guide to the study of gene and protein phylogeny and evolution for those willing to get into the field. This first comment is certainly also valid. Therefore, we have edited the text so that an extra step to assess phylogenetic assumptions after the alignment trimming, and several tools are present in the protocol. We added feedback loops in the protocol (Figure 1).

Row 88-91: 'Feedback loops illustrate the necessity to control the quality of the alignment, to assess phylogenetic assumptions and to test the robustness of the tree, and to go back to previous steps to redo the analysis if necessary.'

We also added several tools proposed by Reviewer 2. This makes the manuscript more comprehensive, especially for sequence alignment and trimming, model selection and phylogenetic analysis. We also edited the text to clarify the specificities of numerous tools and methods so users can select the best fit method depending on their data and objectives, depending on the size of their datasets. More specifically, we have edited at the following paragraphs:

Row 248-256 : 'Once the alignment is completed, it is necessary to select the positions and regions that will be used for the phylogenetic inference. Poorly aligned positions and highly variable regions are not phylogenetically informative, because these positions might not be homologous or subject to saturation. These positions should be manually or automatically excluded prior to the phylogenetic analysis. The resulting sub-MSA maximizes the phylogenetic signal of the alignment [51]. Alignment trimming can be done manually or using appropriate programs. The completeness of alignments can be quantified and phylogenetic informative regions of the alignment can be selected using appropriate tools, such as **Guidance 2** [52], **AliStat** [53], **Gblocks** [51,54], **trimAl** [55], **BMGE** [56] and **Noisy** [57] (**Table 5**).'

FACULTY OF MEDICINE
Lund University
Department of Laboratory Medicine
Translational Cancer Research

We also included more steps to the protocol, including a paragraph on the validation of phylogenetic assumptions, based on a new tool that has been implemented in IQ-TREE, and another paragraph on bootstrapping methods.

Row 261-269: ‘Most phylogenetic methods rely on simplifying assumptions stating for example that all sites in the alignment evolved under the same tree, that mutation rates have remained constant, and that substitutions are reversible. Once the alignment is performed and the sites selected for phylogenetic inference, a recent phylogenetic protocol recommends assessing those phylogenetic assumptions when possible [58]. If the phylogenetic data violate these assumptions, the phylogeny and evolutionary analyses can be biased with most common phylogenetic programs [59]. Several statistical methods have been developed. Recently, tests for all these assumptions have been included in IQ-TREE [60,61]. It is also possible to use the R package MOTMOT [62].’

I welcome that you cite a lot of databases, but could you move their citations from where they are to right after the name? Sometimes, a citation is at the end of the sentence and might be mistaken for a citation to something else (e.g., other databases or multiple sequence alignment).

Yes, thanks for pointing this out. Now, we have edited through all the manuscript the text to consistently have citations right after the name of every database and for software every time they appear in a paragraph.

Regarding the phylogenetic analysis (L194-L364), I compliment you on a valiant attempt to cover a large and complex field. However, I do not think you succeeded because I found gaping holes:

1. You seem unaware of previous phylogenetic protocols, one of which appeared recently in NAR Genomics & Bioinformatics (2, lqaa041).

Thanks for pointing this paper out to us. Now, we have edited figure 1 and the text according to this recently published protocol, and cite this work. We have edited the text on:

Row 264-265: ‘a recent phylogenetic protocol recommends assessing those phylogenetic assumptions when possible [58]’

Row 370-373: ‘For model-based methods, a recent phylogenetic protocol recommends to test the goodness of fit between tree, model and data using a parametric bootstrap [58,128]. Bayesian inference method calculates posterior probabilities, which measure branch support instead of bootstrap values.’

2. Because the manuscript does not present something novel but summarizes bioinformatics tools and resources, chiefly databases, you need to be comprehensive. Unfortunately, you were not comprehensive. This applies to both multiple sequence alignment methods and phylogenetic methods.

Lund University Cancer Center, Division of Translational Cancer Research, Department of Laboratory Medicine, Lund, Sweden. Mail: florian.jacques@med.lu.se

We have edited the text to highlight that the main aim of this work is to provide a roadmap for the study of gene and protein evolution for the broad scientific community and scientist that are new to the field. Our intention is indeed to be comprehensive, but also simplify the decision-making process for the reader while choosing the most appropriate tools for their scientific endeavor. Therefore, we believe it is important to have a balance between being exhaustive and practical enough in a way that will also incorporate beginners. This paper does not aim to present all tools that exist for MSA or phylogenetic analysis but rather a set of tools which are diverse, widely used in the scientific community and also user friendly. Specially for beginners in the field, it is easier to navigate a list of software that is actively maintained by the scientific community, and for which getting support (in the form of tutorials, documentations, publications, online support groups) is easier. Still, we added several tools and methods for sequence alignment, trimming, model selection and phylogenetic inference (see responses below). We have edited text:

Row 5: 'a practical guide'

Row 69-70: 'The aim is to provide a practical guide for beginners and more advanced explorers into protein and gene phylogeny and evolution.'

3. Currently, I am comparing the accuracy of 20 multiple sequence alignment methods (i.e., Clustal Omega, CONTRAlign, DIALIGN-TX, Fsa, GramAlign, KAlign, MAFTT (default), MAFFT (EINSI), MAFFT (GINSI), MAFFT (LINSI), MSAProbs, Muscle, Pasta, PicXAA, Poa, Prank, ProbAlign, ProbCons, T-Coffee (fast), and T-Coffee (regressive)). You considered only a few of these.

Thanks for pointing out also these methods. Related to our response in the previous point, we recognize that there are several other tools that have been developed. We have therefore added a majority of these methods in the text and in Table 4.

Thus, we have edited the following:

Row 226-243: 'The progressive approach aligns progressively from the closest to the most distant sequences. It is used by **CLUSTALW** [34], **CLUSTAL Omega** [35], **MUSCLE** [36], **PRANK** [37,55], **Kalign** [39] and **MAFFT** [40]. Consistency-based methods calculate the best alignment after different pairwise alignments. They are used by **T-COFFEE** [41] and **PROBCONS** [42] and its successor **CONTRAlign** [43], the latter for amino acid sequences only (**Table 4**). **CLUSTALW** [34] and **MUSCLE** [36] are included in **MEGA** [44]. They display web interfaces, as well as **MAFFT** [40], **Kalign** [39], and **PRANKS** [37,55]. **PROBCONS** [42], **T-COFFEE** [41] and **MAFFT** are described to have particularly high accuracy but also high calculation times [45]. They should be restricted to small and intermediate datasets. **CLUSTAL Omega** [35] and **Kalign** [39] are particularly fast, but less accurate [46]. They can be used for datasets of up to 4000 and 2000 sequences, respectively [45,46]. The performances of Muscle are intermediate [46]. **PRANK** is particularly accurate for large sets and closely related sequences. **Bali-Phy** [47] performs a bayesian co-estimation of alignment, phylogeny, and other parameters and is also argued to be very reliable. **PASTA** [48] and **UPP** [49], that uses a machine learning technique, are designed for very large datasets. **MAFFT** offers a wide range of methods, which can be accuracy-oriented, such as L-INS-i, G-INS-I and E-INS-i; or speed-oriented, such as FFT-NS-2, which can be used for up to 30 000 sequences.'

Table 4: List of programs for multiple sequence alignment (*: tools that include web interface)

**Software**	**Features**	**Link**	**References**
BAli-Phy	Multiple sequence alignment of nucleotide and amino acid sequences and phylogenetic analysis using a bayesian approach	http://www.bali-phy.org	[47]
CLUSTAL Omega*	Speed-oriented multiple sequence alignment for nucleotide or amino acid data, suitable for large datasets	https://www.ebi.ac.uk/Tools/msa/clustalo/	[35]
CLUSTALW*	Multiple sequence alignment for nucleotide or amino acid data	https://www.genome.jp/tools-bin/clustalw	[34]
CONTRAlign (ProbCons)	Accuracy-oriented multiple sequence alignment for amino acid data	http://contra.stanford.edu/contralign/	[42,43]
Kalign*	Multiple sequence alignment for nucleotide or amino acid data, suitable for large datasets	https://www.ebi.ac.uk/Tools/msa/kalign/	[39]
MAFFT*	Accuracy-oriented multiple sequence alignment for nucleotide or amino acid data	https://mafft.cbrc.jp/alignment/server/	[40]
MUSCLE*	Multiple sequence alignment for nucleotide or amino acid data	https://www.ebi.ac.uk/Tools/msa/muscle/	[36]
PASTA	Speed-oriented multiple sequence alignment for nucleotide or amino acid data, designed for very large datasets	https ://bioinformaticshome.com/tools/msa/descriptions/PASTA.html	[48]
PRANK/ WebPRANK*	Speed-oriented multiple sequence alignment for nucleotide or amino acid data, should be preferred for close sequences and large datasets	http://wasabiapp.org/software/prank/ https://www.ebi.ac.uk/goldman-srv/webprank/	[37,38]
SATé	Software package for multiple sequence alignments and phylogenetic inference	https ://phylo.bio.ku.edu/software/sate/sate.html	[50]
T-COFFEE*	Accuracy-oriented multiple sequence alignment of nucleotide and amino acid sequences	http://tcoffee.crg.cat/	[41]

UPP	Speed-oriented multiple sequence alignment of nucleotide and amino acid sequences, designed for very large data sets	https://github.com/smirarab/sepp.	[49]

However, several of the tools mentioned by reviewer #2 are not maintained, or have not been updated in several years. Sometimes they are not accessible (links in the publications are broken, do not exist and so one needs to contact the authors to get the source code). We recognize the value of these tools and their contribution to the field, but we believe including them in our manuscript may only create confusion. For those reasons, we have not included Probalign, PicXAA, FSA, GramAlign, and MSAProbs.

4. You mention one method for trimming sites from multiple sequence alignment (GBlocks; in Table 4). There is a suite of other and more suitable methods available (see NAR Genomics & Bioinformatics 2, lqaa024; see also citations 13-21 in that paper).

Yes, we thank the reviewer for this thought. We added a full paragraph on alignment trimming and mention several tools from these publications.

Row 248-256: ‘Once the alignment is completed, it is necessary to select the positions and regions that will be used for the phylogenetic inference. Poorly aligned positions and highly variable regions are not phylogenetically informative, because these positions might not be homologous or subject to saturation. These positions should be manually or automatically excluded prior to the phylogenetic analysis. The resulting sub-MSA maximizes the phylogenetic signal of the alignment [51]. Alignment trimming can be done manually or using appropriate programs. The completeness of alignments can be quantified and phylogenetic informative regions of the alignment can be selected using appropriate tools, such as **Guidance 2** [52], **AliStat** [53], **Gblocks** [51,54], **trimAl** [55], **BMGE** [56] and **Noisy** [57] (**Table 5**).’

5. You mention three model-selection methods but overlooked other more flexible methods (Nature Methods 14, 587-589; Mol. Biol. Evol. 29, 1695-1701; Syst. Biol. 63, 726-742; Mol. Biol. Evol. 34, 772-773; Syst. Biol. 69, 249-264).

Yes, we thank the reviewer also for pointing this out. We included several new tools in the text and in Table 4 (see response above). More specifically, the edits are made in

Row 279-281: ‘More recently, the GHOST model for alignments with variation in mutation rate was introduced and implemented in IQ-TREE.’

Row 288-292: ‘**PartitionFinder 2** [82] can be used with nucleotide and amino acid data. Model test selectors are also included in programs such as MEGA [44] and PhyML (**SMS**) [83]. **ModelFinder** [84] is a model selection method for alignments or nucleotides, codons or amino acids implemented in IQ-TREE [60,61], that includes a flexible model of rate-heterogeneity between sites.’

Table 6: List of programs for molecular evolution model selection

FACULTY OF MEDICINE
Lund University
Department of Laboratory Medicine
Translational Cancer Research

**Software**	**Features**	**Link**	**References**
ModelFinder	Fast model selection with a model of rate heterogeneity between sites (nucleotide, amino acids or codons)	Implemented in IQ-TREE	[84]
ModelTest / jModelTest	Nucleotide substitution model selection	http://evomics.org/resources/software/molecular-evolution-software/modeltest/	[80]
PartitionFinder 2	Molecular evolution model selection (nucleotide or amino acids)	http://www.robertlanfear.com/partitionfinder/	[82]
ProtTest	Aminoacid substitution model selection	https://github.com/ddarriba/prottest3	[81]
SMS	Molecular evolution model selection included in PhyML (nucleotide or aminoacid)	http://www.atgc-montpellier.fr/sms/	[83]

6. Your understanding of the relationship between log-likelihood and the AIC and BIC is wrong (L232-L233), suggesting confusion. You should read Briefings in Bioinformatics (21, 533-565).

We have now edited this sentence to make it more accurate and clear.

Row 283-284: 'For every substitution model, these tools calculate the Bayesian information criterion (**BIC**) [78] and the Akaike information criterion (**AIC**) [79] from the log-likelihood scores.'

7. Your understanding of what the non-parametric bootstrap scores indicate is wrong (see Science 310, 1911-1912).

We acknowledge that the text can be much clearer. Therefore, we have added a full paragraph with information, precision and references on bootstrapping method and other tests of tree robustness. See edits on

Row 355-373: 'Once the phylogenetic tree is obtained, it is recommended to estimate the robustness of the nodes. Most programs of phylogenetic analysis use the non-parametric bootstrapping method [121]. Bootstrapping is an estimate of error used to assess the repeatability of the clade and the how consistently the data support the nodes [121,122]. The characters (e.g., nucleotides or amino acids) are randomly resampled with replacement and a new phylogeny is calculated for each replicate. A bootstrap value is calculated for every node,

indicating the proportion of replicate phylogenies that recovered the node from the initial tree. A bootstrap value of 100% means that the node is supported by all informative characters, while low values mean that only few characters support the node. A bootstrap value above 95% is usually considered very good and a bootstrap value below 75% is generally considered a poor support for the clade. 1000 replicates are often used in phylogenetic analysis. Since bootstrapping can be time consuming, fast approximation methods for phylogenetic bootstrap have been proposed and are implemented in programs such as RAxML or IQ-TREE [123–125]. Other tests of branch support robustness exist, such as the Shimodaira-Hasegawa test [126] and the approximate likelihood ratio test [127], both implemented in PhyML along with the bootstrapping method. FastTree includes only the Shimodaira-Hasegawa test. For model-based methods, a recent phylogenetic protocol recommends to test the goodness of fit between tree, model and data using a parametric bootstrap, rather than non-parametric bootstrap [58,128]. Bayesian inference method calculates posterior probabilities, which measure branch support instead of bootstrap values.'

8. You mention several distance matrix-based phylogenetic methods but do not cite papers describing them.

To clarify, we have complemented citations in the text. Edits are made on

Row 314-315: 'Distance methods include the Unweighted Pair Group Method with Arithmetic mean (**UPGMA**) [91], Neighbor Joining (**NJ**) [92], and Minimum Evolution (**ME**) [93].'

9. Your list of phylogenetic methods is incomplete and misses many important programs (e.g., IQTREE (Mol. Biol. Evol. 32, 268-274), IQTREE2 (Mol. Biol. Evol. 37, 1530-1534), PHYLIP (Felsenstein, 2005), PyCogent (Genome Biology 8, R171), FastME v2.0 (Mol. Biol. Evol. 32, 2798-2800), LSD (Syst. Biol. 65, 82-97) Garli (Syst. Biol. 63, 812-818), PhyloBayes 3 (Bioinformatics 25, 2286-2288), SplitsTree (Mol. Biol. Evol. 23, 254-267)).

Yes, the reviewer is correct that not all programs are presented. This is, as mentioned above, a deliberation to balance the overview with a guide that is accessible also to novices to the field. For phylogenetic analysis tools we have the same criteria as for the MSA (not include tools which are not maintained or are difficult to access). Therefore, we added most of these tools to be more comprehensive, with comments on their specificities. This concerns particularly ML methods and Bayesian inference. (see also responses to comments 2-4, and 9). More specifically, the following is edited:

Row 329-339: 'Programs for ML phylogenetic analysis include **MEGA** [44], **SeaView** [104], **PhyML** [105], **RAxML** [106], **FastTree** [100], **PAML** [107]**, PAUP** [99,103]**, IQ-TREE** [60,61]**, HYPHY** [108], **PHYLIP** [101] and **GARLI** [109,110] (**Table 7**). All of them can be used with nucleotide or amino acid data. **MEGA** [44] and **SeaView** [104] are known to be very user-friendly. They include sequence alignment tools and tree editors. **PhyML** [105] is accurate, easy of use and, like **PAUP** [103] and **MEGA** [44], includes all common models of molecular evolution. **RAxML** [106] and particularly **FastTree** [100] are fast and well suited for large datasets (up to 1 million sequences with FastTree). They use a specific model of rate heterogeneity, in addition to Gamma law and proportion of invariant sites. **IQ-TREE** [60,61], that includes ModelFinder [84] and a very fast bootstrapping method, is reported to be both

fast and accurate [111]. **PAUP** [99,103] is slower than other programs, and uses nucleotide data only.'

Row 343-349: 'The most recent method for phylogenetic reconstruction uses Bayesian inference, that calculates the probability of the molecular evolution model given the data. The main software used for BI-based phylogenetics is **MrBayes** [112] that uses the Markov Chain Monte Carlo (MCMC) algorithm (**Table 7**). **PhyloBayes** [113,114] is a bayesian MCMC sampler for phylogenetic reconstruction with protein data using a specific probabilistic model, well adapted for large datasets and phylogenomics. **Bali-Phy** [47] can also be used for phylogenetic analysis using Bayesian inference.'

Table 7: List of programs for phylogenetic analysis using distance methods, maximum parsimony, maximum lilekihood (ML) and bayesian inference (*: tools that include web interface)

Software	Features	Link	References
APE	R-written package for molecular phylogenetics	http://ape-package.ird.fr	[98]
BAli-Phy	Sequence alignment and phylogenetic inference using a bayesian approach	http://www.bali-phy.org	[47]
BayesTraits	Phylogenetic inference and other evolutionary analyses using Bayesian inference	http://www.evolution.reading.ac.uk/BayesTraitsV4.0.0/BayesTraitsV4.0.0.html	[115]
ETE Toolkit	Visualization and analysis of phylogenetic trees	http://etetoolkit.org/	[116]
FastMe	Fast phylogenetic inference using distance methods.	http ://www.atgc-montpellier.fr/fastme/	[94]
FastTree	Phylogenetic inference using ML for nucleotide (GTR and JC models) and amino acid (JTT and WAG models), and Shimodaira-Hasegawa test. Suitable for very large datasets.	http://www.microbesonline.org/fasttree/	[100]
FigTree	Graphic software for phylogenetic trees	http://tree.bio.ed.ac.uk/software/figtree/	[117]
GARLI	Phylogenetic inference using ML for nucleotide (GTR model), aminoacid (most models) or codon data, with Gamma law and proportion of invariant sites.	http ://evomics.org/resources/software/molecular-evolution-software/garli/	[110]
HYPHY*	Diverse evolutionary analyses including evolution model selection, phylogenetic inference using ML and distance methods and sequence evolution studies	https ://www.hyphy.org/	[108]

IQ-TREE	ML phylogenetic inference, including model selection and ultrafast bootstrapping method. Includes the GHOST evolution model and tests for phylogenetic assumptions.	http ://www.iqtree.org/	[60,61]
ITOL	Visualization and annotation of phylogenetic trees	https://itol.embl.de/	[118]
MEGA	Sequence alignment, model selection, phylogenetic analysis (parsimony, distance methods). Includes all common nucleotide and amino acid evolution models, Gamma law and proportion of invariant sites, and Bootstrapping method.	https://www.megasoftware.net/	[44]
MrBayes	Bayesian phylogenetic inference, ancestral states reconstruction, phylogenetic calibration and other evolutionary analyses	http://nbisweden.github.io/MrBayes/	[112]
PAML	Maximum likelihood phylogenetic inference, estimation of selection strength, ancestral states reconstruction and other analyses	http://abacus.gene.ucl.ac.uk/software/paml.html	[107]
PAUP	Phylogenetic inference using maximum parsimony and ML on nucleotide sequences (all ModelTest models), with Gamma law and proportion of invariant sites and Bootstrapping method.	http://paup.phylosolutions.com/	[99]
PHYLIP	Phylogenetic inference using parsimony, distance methods and ML	https ://evolution.genetics.washington.edu/phylip.html	[101]
PhyloBayes	Phylogenetic inference using Bayesian inference on proteins using a specific probabilistic model	http ://www.atgc-montpellier.fr/phylobayes/	[113]
PhyML*	Phylogenetic inference using ML, ancestral states reconstruction and various evolutionary analyses. Includes all common DNA and protein evolution models and diverse branch support methods (Bootstrap, Shimodaira-Hasegawa, aLTR…).	https://github.com/stephaneguindon/phyml<br><br>Web interface : http://atgc.lirmm.fr/phyml/	[105]
PyCogent	Phylogenetic inference and phylogeny drawing, various evolutionary analyses including partition models and ancestral states reconstruction	https ://github.com/pycogent/pycogent	[95]
RAxML	Phylogenetic inference using ML with nucleotide (GTR) or amino acid data (all common models) with Gamma law or	https://cme.h-its.org/exelixis/web/software/raxml/	[106]

	CAT and proportion of invariant sites. Suitable for large datasets.		
SeaView	Sequence alignment and phylogenetic inference using maximum parsimony, NJ and ML	http://doua.prabi.fr/software/seaview	[104]
SplitsTree	Phylogenetic inference, in particular unrooted trees, or phylogenetic networks	https ://uni-tuebingen.de/fakultaeten/mathematisch-naturwissenschaftliche-fakultaet/fachbereiche/informatik/lehrstuehle/algorithms-in-bioinformatics/software/splitstree/	[97]

10. You really need to ensure that software and methods referred to are cited properly, preferentially every time and with version numbers included.

Yes, we have now been more careful on this. See response above (comments 8 and 10). In essence, we have edited the text to include the historical papers describing the different distance methods, as well as all software and methods.

11. Your figures are unclear, and the colours used are not consistent with a colour palette fit for colourblind people.

We thank the reviewer for pointing this out. Although most colors in our figures are defined by others (webtools and logos), we have changed the red color for yellow in Figure 3.

SPECIFIC COMMENTS

We thank the reviewer for the detailed comments below. To all, we have edited sentences to clarify and increase accuracy as suggested by the reviewers. Specific replies follow below (row numbers refer to those in the originally submitted MS).

L47-L48. Why start with “Although … community”?

Thanks. We removed “Although … community”.

L61. “allowed” > “has allowed”

Lund University Cancer Center, Division of Translational Cancer Research, Department of Laboratory Medicine, Lund, Sweden. Mail: florian.jacques@med.lu.se

Yes, thanks. We replace « allowed » with « has allowed ».

L61-L62. “nucleic acid and protein” > “nucleotides and amino acids” [DNA is a sequence of nucleotides and protein is a sequences of amino acids; therefore, it is not correct to write DNA sequences or protein sequences]

True. We replaced « nucleic acids » and « protein » with « nucleotide and amino acids ».

L62. “In addition, this data is” > “Typically, these data are” [datum is singular of data]

Yes, we changed « is » to « are ».

L66-L67. “navigate these resources can be puzzling” > “navigate through these resources can be challenging”

We changed « puzzling » for « challenging ».

L81. Delete “considerable accompanying”

We deleted « considerable accompanying ».

L84-L86. The figure is not a fully developed protocol like those in NAR Genomics & Bioinformatics 2, lqaa041 [2020].

We have updated the figure and added steps, in accordance with this protocol. We added alignment trimming, validation of phylogenetic assumptions, and bootstrapping as specific tools.

L89. “DNA sequences” > “nucleotide sequences (DNA)” [I am pedantic here, but DNA is a sequence (of nucleotides) so writing DNA sequences is the same as saying a sequence of a sequence of nucleotides]

We changed « DNA sequences » to « nucleotide sequences ».

L89. “and their protein translations” > “and, where applicable, their translations into protein”

We changed « and their protein translations » to « and, where applicable, their trabslations into protein ».

L103. FigTree is not a database!!! It is a phylogenetic tool, so it should be in a different table.

The FigTree is moved to the appropriate table.

L119. “protein sequences” > “amino-acid sequences” [I use a hyphen because amino and acid form a compound adjective]

FACULTY OF MEDICINE
Lund University
Department of Laboratory Medicine
Translational Cancer Research

We changed « protein sequences » into « amino-acid sequences ».

L123. See above.

We changed « protein sequences » into « amino-acid sequences ».

L148. “sequences alignment” > “pairwise sequence alignment” OR “multiple sequence alignment”

We changed “sequences alignment” to “pairwise sequence alignment or multiple sequence alignment”.

L159. “evolutionary relationships” > “evolutionary relationships (see below)”

We added « see below ».

L167 “under FASTA” > “in FASTA”

We changed « under FASTA » to « in FASTA ».

L177. “, i.e., … ancestry” > “(i.e., … ancestry)” [I prefer “that is,” in the sentence and “i.e.,” inside parentheses]

Parentheses are added.

L177-L179. This sentence should be so that the terms homology, orthology, paralogy and xenology are clear and unambiguous.

We rephrased the paragraph to explain more clearly the concepts of homology, orthology, paralogy and xenology.

L184. Include “(define/explain the E-value)”

Thanks, we added a definition of the E-value.

L185. “amino acid sequences” > “amino-acid sequences”

We added the hyphen.

L186. Explain “HH”

We introduced the HHsuite.

Lund University Cancer Center, Division of Translational Cancer Research, Department of Laboratory Medicine, Lund, Sweden. Mail: florian.jacques@med.lu.se

L196. “proteins within” > “proteins, even within”

We changed “proteins within” into “proteins, even within”.

L197. “data, (“ > “data (“

The comma is removed.

L198. “), includes” > “) includes”

The comma is removed.

L202. Delete “sequences”

We deleted « sequences ».

L203. “one after the other” > “, one after the other,”

Two commas were added.

L213-L214. Not “distantly related”; “closely related” [I spoke to Ary and Nick about this] Edited from “distantly related” to “closely related”.

L227. You list the two least realistic models of rate heterogeneity across sites. More realistic models of rate heterogeneity across sites are described in Kalyaanamoorthy et al. (Nature Methods 14, 587-589) and Crotty et al. (Syst. Biol. 69, 249-264).

We introduced these models and added the references.

L232-L233. This sentence is incorrect. The AIC and BIC are computed from the log-likelihood scores; not the other way around, as you write.

We rewrote the sentence to clarify.

Row 279-280: ‘For every substitution model, these tools calculate the Bayesian information criterion (**BIC**) [76] and the Akaike information criterion (**AIC**) [77] from the log-likelihood scores.’

L235-L239. Your description of the reason for doing a non-parametric bootstrap analysis is misleading and is not supported by relevant literature (e.g., Mol. Biol. Evol. 9, 366-369; Mol. Biol. Evol. 30, 1188-1195; Mol. Biol. Evol. 35, 518-522).

We added a paragraph to explain the bootstrapping and other methods to test tree robustness. We added the references.

L247-L250. Include citations after each method or program mentioned, please.

FACULTY OF MEDICINE
Lund University
Department of Laboratory Medicine
Translational Cancer Research

We added the citation immediately after every method and program described.

Lund University Cancer Center, Division of Translational Cancer Research, Department of Laboratory Medicine, Lund, Sweden. Mail: florian.jacques@med.lu.se
