# Supplementary material for: Roadmap to the study of gene and protein phylogeny and evolution—A practical guide
Source: PLoS One. 2023 Feb 24;18(2):e0279597. doi: 10.1371/journal.pone.0279597 (PMC9955684; doi:10.1371/journal.pone.0279597)
Supplement: Attachment — Submitted filename: Response to Reviewers.pdf [file pone.0279597.s007.pdf]

Point-by-point Response to Reviewers

We thank the reviewers for their helpful and insightful comments. We have revised the manuscript and responded to all comments and concerns of Reviewer #2. We believe that this review and our revision has resulted in a much-improved contribution. Below, we reply to all the comments one by one. Our replies are marked in green for when agree and red for when we disagree to some degree. We refer to new intervals of text by their row number in the manuscript.

Reviewer #1: The authors have adequately addressed the most important issues in this revised manuscript. I have no further comment.

Reviewer #2: GENERAL COMMENTS

In general, I still think there is merit in publishing a manuscript like the one submitted. However, I also think the manuscript can improved further. Several aspects of the revision suggest you were in a hurry to finish it, leading, in some cases, to confusion rather than clarity.

If you want the manuscript to become the paper that educators recommend as an introduction to the study of gene and protein phylogeny and evolution, then you may need to invest a bit more of your time on achieving an accurate, easy-to-read presentation of the topics. There is also evidence that you do not understand some of the aspects that you cover. Those have to be addressed so that you present a sound and reliable protocol, with references to critical databases, alignment tools, and phylogenetic methods.

In the following, I list some of the major issues:
1. An example of the above-mentioned misunderstanding appears in Figure 1: The feedback loops should be distinguished from the forward arrows (use different colour or dashed arrows). There are no arrows between "Select phylogenetic method" and the two boxes below it with methods.

Response: We changed the feedback loops to dashed arrows in Figure 1. We added an arrow between "select phylogenetic method" and the methods below.

2. Distance methods are model-based, so they should be listed with the maximum-likelihood and Bayesian methods.

Response: We added the box "selection of molecular evolution model" under the distance methods.

3. You write "Test of robustness", which is misleading. Robustness refers to the quality or condition of being strong and in good condition. A phylogenetic estimate can be robust, yet it may be incorrect. We would like the estimates to be accurate and the methods to be precise and accurate. As for the accuracy, we may test the goodness of fit between the tree, the model of sequence evolution, and the data. A good fit means that the tree and model of sequence evolution provide a good explanation of the data. Yet, we will never know whether that explanation is the correct (others may exist). A poor fit means that something is not right

about the tree, the model of sequence evolution, or both. This is fundamental and it should be clear from your manuscript (which it isn't).

Response: We changed "test of robustness" to "test of reliability" on Figure 1 and in the text. We explain more in detail what a good fit means and we stress in the manuscript that a good fit doesn`t mean that the tree is correct but is just an estimate of goodness of fit between the tree, the model and the data.:

L455-457: "A good fit means that the tree and the model of sequence evolution provide a good explanation of the data but doesn't indicate if the tree in correct or not.".

4. The sections on alignment and phylogenetic analysis (L212-L395) have been improved a lot. However, you appear to ignore the new phylogenetic protocol [1], which you cited in the Introduction. I think it would serve you better if you described the different steps and procedures in the context of the new phylogenetic protocol [1] and/or Figure 1. You know the details and order of actions but newcomers to the field do not and would need a clear framework. The new protocol provides that framework.

Response: We detailed more the new phylogenetic protocol and added information on the two new steps from this protocol: "assessing phylogenetic assumptions" and "test of goodness of fit".

L295-305 "Phylogenetic models rely on simplifying assumptions stating, for example, that all sites in the alignment evolved under the same tree (treelikeness), that mutation rates have remained constant over time (i.e., time-homogeneity), and that substitutions are reversible and, therefore, also stationary (for details on these assumptions, see [72]). If the phylogenetic data violate these assumptions, the phylogeny and evolutionary analyses can be biased [73–75]. Once the alignment is performed and the sites selected for phylogenetic inference, a recent phylogenetic protocol recommends assessing those phylogenetic assumptions when possible [1]. Statistical methods allowing to test stationarity, homogeneity under certain markovian conditions, and treelikeness have been developed and included in IQ-TREE and IQ-TREE2 [76,77]. Homo2.1 [78] is designed for the analysis of compositional heterogeneity in sequence alignments. It is also possible to use the R package MOTMOT [79]."

L453-467: "It is possible that the inferred optimal model of sequence evolution used for the phylogenetic analysis is inadequate. Once a phylogenetic tree has been inferred, it is recommended to test the goodness of fit (*i.e.,* the adequacy) between the tree, the model and the data [1]. A good fit means that the tree and the model of sequence evolution provide a good explanation of the data but doesn't indicate if the tree in correct or not. The goodness of fit can be tested using a parametric bootstrap [149]. Parametric bootstrap consists in using the optimal tree and the optimal model as an input to simulate sequence evolution to generate pseudo-data with a Monte Carlo simulation. Sequence generating data, such as **SeqGen** (https://github.com/rambaut/Seq-Gen/releases/tag/1.3.4, [150]) can be used. The goodness of fit is calculated from the difference between the unconstrained and constrained (*i.e.*, assuming the optimal tree and the optimal model) log-likelihoods of the real data and the pseudo-data. If the fit is poor, it can be good to check the alignment and the selected set of sites and/or the sequence evolution model (feedback loops on Fig.1). Several methods have been developed to test the adequacy of the data [151]. The Goldman-Cox (GC) test can be used with several

phylogenetic programs such as PAUP. Most Bayesian phylogenetic programs employ the posterior predictive (PP) test."

We stress the importance of testing phylogenetic assumptions prior to phylogenetic analysis and the goodness of fit. Our Figure 1 and our manuscript is now based on this new protocol.

5. Regarding multiple sequence alignment, I think you need to consider and cite some of the excellent papers by Morrison (e.g., Is Sequence Alignment and Art or Science; Syst. Bot. 40, 14-26 [2015]). You gloss over the challenges and pitfalls, some of which are illustrated in Golubchik et al. (Mol. Biol. Evol. 24, 2433–2442 [2007]), thereby belittling the challenge it is to obtain a trustworthy multiple sequence alignment. Apart from the progressive and consistency-based approach (mentioned on L224-L227), there are other ways to obtain a multiple sequence alignment. You should at least mention some of these other strategies and cite the relevant literature (or reviews of that literature). Further, the consistency-based approach is not well described (L226-L227).

Response: We added a paragraph to discuss more in detail the challenge of obtaining accurate MSA where we cite Morrison`s paper and Golubchik`s work.

L258-277: "In practice, finding the accurate multiple sequence alignment can be challenging for several reasons. For example, computer programs are not based explicitly on the hypothesis of homology between aligned residues. They consider homology exclusively as similarity, and do not consider the other conditions for homology such as congruence and conjunction [61]. Furthermore, one should keep in mind that the alignment with the best score is not necessarily biologically correct. It is also difficult to get a good alignment for sequence that have diverged significantly and sharing low identity. In this case, for protein-coding sequences, amino-acid data should be preferred over nucleotide data, since it is possible, for example, to consider the biochemical similarity of substitutions of amino acids. Alignment software require defining a gap-opening penalty and a gap-extension penalty, but these values are arbitrary. It is common that different sequences in the alignment do not have the same length, for biological or experimental reasons. It is recommended to keep end gaps unpenalized [62]. Furthermore, indels are reported to affect the accuracy of MSA programs. It is recommended to use several MSA programs for sequences that contain indels [63]. MAFFT is reported to be the most accurate program in the case of sequence with non-overlapping deletions and in the case of alternatively spliced gene products [63]. Another difficulty concerns the case of repeated sequences with different numbers of repeats. Here, a domain of one sequence can be homologous to several domains of another sequence. Single nucleotides or small sequences can also be repeated like in the case of microsatellites, as well as entire protein domains. In the last case, it is recommended to excise the repeated domains [62]."

We describe more in detail consistency-based methods, and we cite the other MSA methods:

L242-244: "Other approaches include the iterative refinement method, which is also included in **MAFFT** and **Muscle** [51], the genetic algorithms, used by SAGA [52], and the methods using hidden Markov models, such as SAM [53].".

6. You should mention the minimum reporting standard for multiple sequence alignments (it

was presented in Ref. 53) when introducing trimming of multiple sequence alignment. The reason for doing so is given in Ref. 53.

Response: We added a mention to this minimum reporting standard:

L287-288: “A minimum reporting standard has been developed to quantify the alignment completeness, and implemented in **AliStat** [66].”

7. The section entitled “Test of robustness of phylogenetic trees” needs to be rewritten. For a start, choosing to use the word “robustness” is not good. Robustness usually refers to how accurate methods are when the assumptions of the methods are violated by the data (see Yang 2014; Molecular Evolution). Other words like accuracy, consistency and precision are more appropriate. In this context, note that:

- Accuracy means ‘the quality or state of being correct or precise’
- Consistency means ‘the quality of achieving a level of performance which does not vary greatly in quality over time’
- Precision means ‘the quality, condition, or fact of being exact and accurate’

Your description of bootstrapping is incorrect; it is not “an estimate of error” (L349).

Response: We rewrote the part on non-parametric boostrap:

L411-437“Once the phylogenetic tree is obtained, it is recommended to estimate the reliability of the clades. Most programs of phylogenetic analysis use the non-parametric bootstrapping method [142]. It is a resampling technique used to assess the repeatability of the clade and the how consistently the data support the nodes [143]. The sites in the alignment (e.g., nucleotides or amino acids) are randomly resampled with replacement and a new phylogeny is calculated for each replicate [142]. A bootstrap value is calculated for every internal branch, indicating the proportion of replicate phylogenies that recovered the clade. A bootstrap value of 100% means that the node is supported by all informative characters, while low values mean that only few characters support the node. Bootstrapping gives a measure of the consistency of the estimate, but it is not a measure of the accuracy of the tree [144]. The number of replicates that are necessary to obtain a good accuracy of the bootstrap value depends on the bootstrap value. For example, for a 1% confidence interval on a bootstrap value of 95, 2000 replicates are necessary [143].

Since bootstrapping can be time consuming, UFBoot and UFBoot2, fast approximation methods for phylogenetic bootstrap, have been developed and are implemented in programs such as RAxML or IQ-TREE [130,145,146]. They are also less biased than other non-parametric bootstrapping methods and robust against moderate model violations. While other bootstrapping methods tend to underestimate the probabilities of the clade of being correct, the bootstrap values from UFBoot and UFBoot2 truly reflect the probability of the clade of being correct, simplifying the interpretation of Bootstrap values (a bootstrap value of 95% indicate a probability of 95% to be correct) [145]).

With ML-based phylogenetic inference, the Shimodaira-Hasegawa test, and its improved version AU [147], are designed to evaluate alternative phylogenetic hypotheses, and test if a tree is better supported than another one. It can be used with PAUP, PhyML, FastTree and IQ-TREE. The approximate likelihood ratio test [148] is another test of phylogenetic relationship,

implemented in PhyML along with the bootstrapping method. Bayesian inference method calculates posterior probabilities, which measure branch support instead of bootstrap values.".

We changed the title to "Test of reliability of phylogenetic trees". We replaced "nodes" with "branches". We also added a few comments on the interpretation of bootstrap, and how UFBoot provides a less biased bootstrap estimate, that facilitates the interpretation of bootstrap values. We changed "sites" into "characters". We explain more in detail the non-parametric bootstrap.

In my previous review, I drew your attention to the correct interpretation of bootstrapping, but you ignored that, which was unwise. Instead, you cite Hedges [120], which focuses on the number of bootstrap replicates needed gain an accurate estimate of the bootstrap P value, and not on the interpretation. You also need to note that bootstrap P values are both data and method dependent. It is the sites in the alignment, not "characters" (L350), which are sampled randomly with replacement, and the correct citation is missing: "each replicate" > "each replicate [Felsenstein 1985; Evolution 39, 783-791]".

Response: We added a reference to Felsenstein`s paper on non-parametric bootstrap.

Bootstrapping, as described by Felsenstein and implemented by many since then, is a non-parametric bootstrap approach. Using it gives us an insight into the consistency of the data (i.e., do we have enough sites in the data to consistently infer the same tree). It does not tell us whether the inferred tree is accurate.

Response: We added a comment to stress that non-parametric boostrap is not a measure of the accuracy of the tree "Bootstrapping gives a measure of the consistency of the estimate, but it is not a measure of the accuracy of the tree."

You write that a "bootstrap value is calculated for every node" (L352). This is incorrect. The value is computed for every internal edge/branch/split that separates the sequences.

You write "from the initial tree" (L353). This is incorrect. It can be done for any tree, even the consensus tree (IQ-TREE often list the values for the ML tree and the consensus tree; and these trees may not be identical).

Response: We corrected the sentence into "A bootstrap value is calculated for every internal branch, indicating the proportion of replicate phylogenies that recovered the clade."

You write "A bootstrap value …for the clade". Who did you quote? There has been some disagreement on the interpretation of bootstrap P values. New methods like those from von Haeleser's group [121, 122] have clarified the matter considerably.

We added a comment on the interpretation of boostraps values and how the UFBoot method facilitates their interpretation. L424-431: "Since bootstrapping can be time consuming, UFBoot and UFBoot2, fast approximation methods for phylogenetic bootstrap, have been developed and are implemented in programs such as RAxML or IQ-TREE [130,145,146]. They are also less biased than other non-parametric bootstrapping methods and robust against moderate model violations. While other bootstrapping methods tend to underestimate the probabilities of the clade of being correct, the bootstrap values from UFBoot and UFBoot2 truly reflect the

probability of the clade of being correct, simplifying the interpretation of Bootstrap values (a bootstrap value of 95% indicate a probability of 95% to be correct) [145]).”

You write “1000 replicates …” (L356). This should be spelled out, and you should probably mention how that fits with Hedges [120].

Response: We added comments on the number of bootstrap according to Hedges, “The number of replicates that are necessary to obtain a good accuracy of the bootstrap value depends on the bootstrap value. For example, for a +-1 confidence interval on a bootstrap value of 95, 2000 replicates are necessary”.

You write “Other branch support methods exist” (L359) and then refer to the Shimodaira-Hasegawa test [124]. The bootstrap method is not a “branch support method”, so it is not correct to say “Other branch …”. Moreover, the Shimodaira-Hasegawa test is not a branch support test either. It is a test designed to compare the ML tree to other trees.

Response: We explain more in details the SH test and rewrote that part of the section:

L432-437: “With ML-based phylogenetic inference, the Shimodaira-Hasegawa test, and its improved version AU [147], are designed to evaluate alternative phylogenetic hypotheses, and test if a tree is better supported than another one. It can be used with PAUP, PhyML, FastTree and IQ-TREE. The approximate likelihood ratio test [148] is another test of phylogenetic relationship, implemented in PhyML along with the bootstrapping method. Bayesian inference method calculates posterior probabilities, which measure branch support instead of bootstrap values.”

8. I have not included any specific comments on the two case studies, mainly because I have already used far more time on this review than I had available. That said, the two sections do need some revision.
9. None of the figures are as clear as is necessary for publication. In fact, I can’t read them.

The Bibliography is clearly set using a French application and do not appear to comply with PLoS One’s style. Please ensure that the Bibliography complies with the journal’s formatting style.

We modified the bibliography according to PLoS One`s style.

SPECIFIC COMMENTS
L25: “Here a” > “Here we present a”
Response: We changed “Here a” into “Here we present a”.

L26: “nucleic acid” > “DNA”
Response: We changed “nucleic acid” into “DNA”.

L27: Delete “is presented”
Response: We deleted “in presented”.

L33: “proto cells to modern organisms” > “primitive cells to modern cells and multicellular organisms,”
Response: We changed “proto cells to modern organisms” into “primitive cells to modern cells and multicellular organisms,”.

L36: “sequence composition of” > “information content in”
Response: We changed “nucleic acid” into “DNA”.

L38-L40: Poor sentence structure

L41: “mutations that” > “mutations (including substitutions) that”
Response: We changed “mutations that” into “mutations (including substitutions) that”.

L43: “all” > “both”
Response: We changed “all” into “both”.

L45: “past decades” > “past four decades”
Response: We added “four”.

L48: “taxa” > “taxa, genes or genomic components (e.g., transposable elements)”
Response: We added “, genes or genomic components (e.g., transposable elements)” in the sentence.

L52: “the others” > “other species”
Response: We changed “the others” into “other species”.

L53: “infection diseases” > “infectious diseases”
Response: We changed “infection” into “infectious”.

L59: Delete “both”
Response: We deleted “both”.

L65: Delete “the”
Response: We deleted “the”.

L66: “and of” > “as well as”
Response: We changed “and of” into “as well as”.

L67: “processes, following” > “processes. In so doing, we follow”
Response: We changed “processes, following” into “processes. In so doing, we follow”.

L68: “used by many and maintained” > “maintained and used by many”
Response: We changed “used by many and maintained” into “maintained and used by many”.

L75: Delete “peer-reviewed”
Response: We did not delete “peer-reviewed” because this exact sentence has to be included in the manuscript for protocols, according to the editor`s instructions for authors.

L80: Delete “digitalized

Response: We deleted “digitalized”.

L82: “nucleic acid or amino acid sequence” > DNA or amino-acid sequences”
Response: We changed “nucleic acid or amino acid sequence” into “DNA or amino-acid sequences”.

L88: Perhaps state that the protocol is based on that in [1].
Response: We added a reference to the new protocol (ref [1]) in the figure`s caption.

L89: “Nucleic acid” > “DNA”
Response: We changed “nucleic acid” into “DNA”.

L95: ”browser focusing on chordates that contains” > ”browser that focuses on chordates and contains”
Response: We changed “browser focusing on chordates that contains” into “browser that focuses on chordates and contains”.

L97: “This” > “The”
Response: We changed “This” into “The”.

L99” Delete “Overall,”
Response: We deleted “Overall”.

L105: “rendered” > “provided”
Response: We changed “rendered” into “provided”.

L114: “provides several” > “provides access to several”
Response: We changed “provides several” into “provides access to several”.

L116: “under” > “in the”
Response: We changed “under” into “in the”.

L117: “nucleotide sequences” > “nucleotide”
Response: We changed “nucleotide sequences” into “nucleotide”.

L118: Delete “pasted to fasta file”
Response: We deleted “pasted to fasta file”.

L129: “format and the” > “format. The”
Response: We changed “format and the” into “format. The”.

L129: “interlinks” > “links”
Response: We changed “interlinks” into “links”.

L130: “allows to batch download the” > “facilitates batch downloads of”
Response: We changed “allows to batch download the” into “facilitates batch downloads of”.

L132: “databases” > “databases (such as …)”
Response: We changed “nucleic acid” into “DNA”.

L136: “The KEGG focuses” > “The KEGG database focuses”
Response: We changed “nucleic acid” into “DNA”.

L148-L156: You alternate between “3-dimensional” and “3D”. Be consistent
Response: We wrote “3-dimensional” in the whole manuscript to homogenize.

L149: Delete “the”
Response: We deleted “the”.

L153: Place “i.e., … family” between parentheses
Response: We added parentheses.

L159: You write “based on …, or both”. Perhaps separate the databases accordingly.
Response: We agree the sentence was unclear. All three databases use structural similarity, functionality, and evolutionary relationship. We changed the sentence to make it more understandable:
L162-163: “Proteins are classified into different categories based on structural similarity, functionality and evolutionary relationship (**Table 2**).”

L163: You write “based on the presence or absence of … strands”. I think you need to elaborate a bit further on this. There is SCOPe classification system to consider but you also need to consider the work by Sun et al. (Current Protocols in Protein Science (2004) 17.1.1-17.1.189)

Response: We added precisions on the structural classification.
L166-169: “Most proteins belong to one of the five main structural classes (α, β, α/β, α+β, multi-domain), defined respectively by the presence in the structure of α-helices, β-strands, both α-helices and β-strands, segregated α-helices and β-strands, or none of these characteristics.”
We added mention to two other classification systems:
L172-179: “Similarly, the Class, Architecture, Topology, Homology database (**CATH**) proposes a five-level classification of protein domains. The first three levels, namely class, architecture and topology, are based on structural homology. The last two, homologous superfamily and family, are based on sequence, structure and functional data, and sequence identity, respectively [29]. The Families of Structurally Similar Proteins (**FSSP**) provides a classification of proteins of the PDB based on a structure comparison algorithm, that calculate a structural similarity score between protein chains. These similarity scores are used to create a classification of protein structures [30,31].”

L164: “class” > “classification”
Response: We kept class instead of classification because we are talking about the protein “class” in the SCOPe system, not the protein classification.

L177: “[16], that combines” > “[16]. It combines”
Response: We changed “[16], that combines” into “[16]. It combines”.

L186: Delete “whole”
Response: We deleted “whole”.

L188: “hemoglobine” > “haemoglobin”
L189: “hemoglobine” > “haemoglobin”
L190: “myoglobine” > “myoglobin”
Response: We changed “hemoglobin” haemoglobin” and “myoglobine” into “myoglobin”.

L197: Include a citation to the E-value. You might know it, but you are targeting beginners who can be assumed to be ignorant.
Response: We added a citation of Kerfeld and Scott, 2011, for the E-value.

L206: “retrieving from” > “retrieving sequences from”
Response: We changed “retrieving from” into “retrieving sequences from”.

L213: “related to each other” > “related to each other in an evolutionary sense” [they can be related to each other on other senses]
Response: We changed “related to each other” into “related to each other in an evolutionary sense”.

L220: “row one” > row of a matrix, one”
Response: We changed “row one” into “row of a matrix, one”.

L230-L231: Poor sentence structure and you probably mean “user interface”, rather than “web interfaces”
Response: We changed “web interface” into “user interface” and rewrote the sentence.

L233: “calculation times” > “execution times”
Response: We changed “calculation times” into “execution times”.

L236-L237: You need a reference to support this statement, and you then need to cite Golubchik et al. (Mol. Biol. Evol. 24, 2433–2442 [2007]), who reported the opposite for PRANK.
Response: We remastered the sentence on the specificity of Prank: L252: “**PRANK** is meant for closely related sequences [57].”

L238: “that” > “which”
Response: We changed “that” into “which”.

L242: “web interface” > “user interface”
Response: We changed “web interface” into “user interface”.

L253: “quantified and phylogenetic informative” > “quantified using AliStat [53] and phylogenetically informative”
Response: We changed “quantified and phylogenetic informative” into “quantified using AliStat [53] and phylogenetically informative”.

L258: “stating for example that” > “stating, for example, that”
Response: We changed “stating for example that” into “stating, for example, that”.

L259: “constant” > “constant over time (i.e., time-homogeneity)”
Response: We changed “constant” into “constant over time (i.e., time-homogeneity)”.

L260: “reversible” > “reversible and, therefore, also stationary (for details on these assumptions, see Jayaswal et al. (Syst. Biol. 63, 726-742 [2014]) and papers cited therein)”
Response: We changed “reversible” into “reversible and, therefore, also stationary (for details on these assumptions, see Jayaswal et al. (Syst. Biol. 63, 726-742 [2014]) and papers cited therein)”.

L261: You write “can be biased [58]”. It would be more appropriate to cite Syst. Biol. (53, 623-637; 53, 638-643) because these papers used data generated by simulation (hence, the truth is known).
Response: We added a citation of Syst. Biol. (53, 623-637; 53, 638-643).

L262: “recent phylogenetic protocols recommend … possible [1]” > “a recent phylogenetic protocol [1] recommends … possible”
Response: We changed “recent phylogenetic protocols recommend … possible [1]” into “a recent phylogenetic protocol [1] recommends … possible”.

L264: “developped" > “developed”
Response: We changed “developped” into “developed”.

L264: You write “tests for all these assumptions”. This is not true. Some have been included in IQ-TREE and IQ-TREE2. I agree that the matched-pairs tests of homogeneity (Bioinformatics 22, 1225-1231) are of relevance, but only for some Markovian conditions. One of these is implemented in Homo 2.1 (https://github.com/lsjermiin/Homo.v2.1).
Response: We changed “tests for all these assumptions” into “Statistical methods allowing to test stationarity, homogeneity under certain markovian conditions, and treelikeness”.

L266: “Selection of the molecular evolution model” > “Selection of the optimal model of sequence evolution”
Response: We changed “Selection of the molecular evolution model” into “Selection of the optimal model of sequence evolution”

L267: Several papers and book chapters have described model selection in general terms. I think it would be wise to cite some of these (e.g., Front. Genet. 6, 319 [2015; Meth. Mol. Biol. 1525, 379-420 [2017]; DOI 10.1007/978-1-4939-6622-6_15).
Response: We added references to those papers.

L268: “Nucleotide or amino acid substitution exist” > “Several models of nucleotide or amino-acid substitution exist [REF]”. The statement requires some citations.
Response: We changed “Nucleotide or amino acid substitution exist” into “Several models of nucleotide or amino-acid substitution exist” and added reference to the phylogenetic handbook (Yang, Oxford University Press, 2014).

L270: you cite Yang 2006; you should cite is more recent book from 2014.
Response: We added a reference to this book.

L273: You write “Each model … IQ-TREE [75]”. The set of models described here differ from the substitution models described in L271-L274. They are homotachous models of rate-heterogeneity across sites, so name them appropriately. You forgot to include the

PDF/FreeRate model proposed by Yang (Genetics 139, 993-1005 [1995]) and now included in ModelFinder, IQ-TREE and PhyML.
Response: We rewrote the section to better separate the substitution models and the rate-heterogeneity models. We explain more in detail what rate-heterogeneity refers to. We also added reference to the FreeRate model and the relevant literature.

L331-338: "These substitution models can be associated with models of substitution rate-heterogeneity between sites. Mutation rates and selective pressure may vary among sites, due to different roles in the structure and function of the gene or protein. The most common rate heterogeneity model are the Gamma distribution (G) and the proportion of invariant nucleotide or amino acid sites (I). Every substitution model can be associated with G, I or both. The FreeRate model, a more complex model of rate heterogeneity [94], is included in ModelFinder, PhyML and IQ-TREE. More recently, the GHOST model for alignments with variation in mutation rate was introduced and implemented in IQ-TREE [95]."

L279: "every substitution model" > "model of sequence evolution (i.e., combination of substitution model and rate-heterogeneity across sites model)"
Response: We changed "every substitution model" into "model of sequence evolution (i.e., combination of substitution model and rate-heterogeneity across sites model)".

L281: "optimizing" > "minimising"
Response: We changed "optimizing" into "minimising".

L284: Move "PartitionFinder 2 …[81]" to L288. The logic here is that ModelFinder is for a single partition and it supersedes ModelTest, jModelTest and ProtTest. PartitionFinder 2 is for multiple partitions.
Response: We moved the mention of PartitionFinder2 to the end of the section.

L293: Replace the sentence with "Phylogenetic trees may consider the topology and the branch lengths (phylograms) or just the topology (cladograms)"
Response: We replaced the sentence with "Phylogenetic trees may consider the topology and the branch lengths (phylograms) or just the topology (cladograms)".

L294: "tree building methods" > "tree-building methods" [compound adjective]
Response: We changed "tree building methods" into "tree-building methods".

L295: "distance" > "distances"
Response: We changed "distance" into "distances".

L295-L206: "number of differences" > "numbers and types of differences"
Response: We changed "number of differences" into "numbers and types of differences".

L296: "to reconstruct" > "and they use this matrix to reconstruct"
Response: We changed "to reconstruct" into "and they use this matrix to reconstruct".

L296-L297: The sentence is wrong. Distance methods are also character-based methods.

We left the distinction between distance methods and character-based methods, since this classification is common in reviews and books of the field (e.g. De Bruijn 2014, Muntjal el al 2019…).

L299: “classic” > “classical”
Response: We changed “classic” into “classical”.

L303: Include citation after “long branch attraction”
Response: We added a reference to the review by Bergsten, 2005.

L309: “mean” > “Mean”
Response: We changed “mean” into “Mean”.

L310: “[91].” > “[91] tree-inference methods.”
Response: We added “tree-inference methods.”

L312: “amino acid data” > “amino-acid data” [compound adjective]
Response: We changed “amino acid data” into “amino-acid data”.

L315: “but also” > “and”
Response: We changed “but also” into “and”.

L316: Delete “,”
Response: We deleted this comma.

L317: “in R language that” > “in the R language. It”
Response: We changed “but also” into “and”.

L320: “ML” > “maximum likelihood [ML]”. I think this is the first time that you typed ML, so it has to be defined, even though you and I both know what it refers to
Response: We left ML because the term has been defined earlier in the text (L361).

L322: “ML methods” > “Maximum-likelihood methods”. When starting a sentence, it is good practice to spell out the abbreviation (or number)
Response: We changed “ML methods” into “Maximum-likelihood methods”.

L322: “probability” > “likelihood”
Response: We changed “probability” into “Maximum-likelihood methods”.

L323: “ML aims” > “Maximum likelihood aims”
Response: We changed “ML” into “Maximum likelihood”.

L323: “combinations of model” > “combinations of trees and model”
Response: We changed “combinations of model” into “combinations of trees and model”.

L327: “amino acid data” > “amino-acid data”
Response: We changed “amino acid data” into “amino-acid data”.

L328: “is accurate” > “is reported as being accurate”
Response: We changed “is accurate” into “is reported as being accurate”.

L329: “all common models of molecular evolution” > “many common models of substitution” or “many common models of sequence evolution” [choose the most accurate statement”

Response: We changed “all common models of molecular evolution” into “many common models of sequence evolution”.

L331: “rate heterogeneity … invariant sites” > “rate-heterogeneity across sites (…)”. Here, you replace “…” with the model of that you refer to

Response: We changed the sentence and cite the method mentioned and relevant publication: L396: “They use CAT, a specific model of rate heterogeneity, faster than G [129], in addition to Gamma law and proportion of invariant sites.”

L333: “that” > “which”

Response: We changed “that” into “which”.

L333: “method” > “method [REF]”. Here you need to replace “REF” with the relevant citation to the method implemented in IQ-TREE and IQ-TREE 2

Response: We added a reference to the paper by Hoang et al, 2017, describing UFBoot2, and mention the tool in the text.

L336: “recent method” > “recently-developed method”

Response: We changed “recent method” into “recently-developed method”.

L336: “inference, that” > “inference (BI). It”

Response: We changed “inference, that” into “inference (BI). It”.

L337: “calculates the probability” > “calculates the posterior probability”

Response: We changed “calculates the probability” into “calculates the posterior probability”.

L337: “the molecular evolution model given” > “the tree and model of sequence evolution, given”

Response: We changed “the molecular evolution model given” into “the tree and model of sequence evolution, given”.

L338: “ [109] that” > “ [109]. It”

Response: We changed “[109] that” into “[109]. It”.

L362: “inbcludes” > “includes”

Response: We corrected the typo.

L365: “taxa” > “sequences”

Response: We changed “taxa” into “sequences”.

L366: “addressed question” > “question asked”

Response: We changed “addressed question” into “question asked”.

L370: “taxa” > “sequences”

Response: We changed “taxa” into “sequences”.

L370: “studied ingroup” > “ingroup of interest”
Response: We changed “studied ingroup” into “ingroup of interest”.

L373: “identified, … viruses,” > “included”
Response: We changed “identified, … viruses,” into “included”.

L375: “of the longest branches” > “between the most dissimilar sequences in the tree”
Response: We changed “of the longest branches” into “between the most dissimilar sequences in the tree”.

L378: “exported using … a graphical” > “visualised using a graphical”
Response: We changed “exported using … a graphical” into “visualised using a graphical”.

L394: You should include Geneious (https://www.geneious.com/) in the table and mention it in the text.
Response: We added Geneious in the table and in the text.

L397: “allows” > “allow users”
Response: We changed “allows” into “allow users”.

L404: Here you should only write MP, ML and BI. Note that you need to define MP somewhere earlier on in the text
Response: We wrote MP, ML and BI. MP is defined earlier in the text.

L405: “molecular” > “sequence”
Response: We changed “molecular” into “sequence”.

L416: In this section, you use the term “mutations”; typically the term “substitutions” is used
Response: We changed “mutations” into “substitutions” in the whole section.

L417: “The strength” > “The type and strength”
Response: We changed “The strength” into “The type and strength”.

L417: “can be calculated. This” > “may be of interest. It”
Response: We changed “can be calculated. This” into “may be of interest. It”.

L418: “ration” > “ratio”
Response: We corrected the typo.

L421: “The ratio … 1, that” > “If dN/dS > 1, then the”
Response: We changed “The ratio … 1, that” into “If dN/dS > 1, then the”.

L426: “Phylogenetic calibration” > “Molecular dating of events”
Response: We changed “Phylogenetic calibration” into “Molecular dating of events”.

L427: “event using events” > “events, using other events”
Response: We changed “event using events” into “events, using other events”.

L440-L444: This can be stated more succinctly

Response: We rewrote the sentences in a more understandable way to introduce coevolution.

L450: Why do you refer to Table 8 here?
Response: We moved the reference to Table 8 to the appropriate sentence.

L457: “species” > “traits”
Response: We changed “species” into “traits”.

L462-L466: This needs to be stated better
We rewrote the sentence:
L554-557: “Phylogenetic trees, in complement with genomics tools and databases, can be used to study genome evolution; and identify evolutionary events such as be mutations, insertions, deletions, gene or genome duplications, genome re-organization, chromosomal rearrangements, polyploidization events or genetic exchanges.”

L477: “Population genetics often studies” > Population geneticists often study”
Response: We changed “genetics” into “geneticists”.

L483: “of” > “at”
We changed “of” into “at”.

L484: “studies, for a full review see” > “studies; for a full review, see”
We changed “studies, for a full review see” into “studies; for a full review, see”.

L493: “in Å” > “in ångström”
We changed “in Å” into “in ångström”.

L494: You write “can be calculated”. Which program?
We added “using PyMol”.

L495: “3-dimentional” > “3D”
To be more consistent, we wrote “3-dimentional” in the whole manuscript.

L496: “amino acid” > “amino-acid”
We changed “amino acid” into “amino-acid”.

L498: Here I think you need to include Alpha fold (Nature 596, 583–589 [2021])
We included Alpha fold in the text and the table.
